# Gender on the Brain: A Case Study of Science Communication in the New Media Environment

**DOI:** 10.1371/journal.pone.0110830

**Published:** 2014-10-29

**Authors:** Cliodhna O’Connor, Helene Joffe

**Affiliations:** Division of Psychology & Language Sciences, University College London, London, United Kingdom; Centre national de la recherche scientifique, France

## Abstract

Neuroscience research on sex difference is currently a controversial field, frequently accused of purveying a ‘neurosexism’ that functions to naturalise gender inequalities. However, there has been little empirical investigation of how information about neurobiological sex difference is interpreted within wider society. This paper presents a case study that tracks the journey of one high-profile study of neurobiological sex differences from its scientific publication through various layers of the public domain. A content analysis was performed to ascertain how the study was represented in five domains of communication: the original scientific article, a press release, the traditional news media, online reader comments and blog entries. Analysis suggested that scientific research on sex difference offers an opportunity to rehearse abiding cultural understandings of gender. In both scientific and popular contexts, traditional gender stereotypes were projected onto the novel scientific information, which was harnessed to demonstrate the factual truth and normative legitimacy of these beliefs. Though strains of misogyny were evident within the readers’ comments, most discussion of the study took pains to portray the sexes’ unique abilities as equal and ‘complementary’. However, this content often resembled a form of benevolent sexism, in which praise of women’s social-emotional skills compensated for their relegation from more esteemed trait-domains, such as rationality and productivity. The paper suggests that embedding these stereotype patterns in neuroscience may intensify their rhetorical potency by lending them the epistemic authority of science. It argues that the neuroscience of sex difference does not merely reflect, but can actively shape the gender norms of contemporary society.

## Introduction

On 2 December 2013, the well-known scientific journal PNAS published an early online edition of an article entitled “*Sex differences in the structural connectome of the human brain*”, which purported to reveal “*fundamental sex differences*” in the structural connectivity of male and female brains [1]. In the days following its release, this article provoked a flurry of coverage in the international print and electronic media. These discussions afford an illuminating example of how neuroscience research on sex differentiation is interpreted and employed in contemporary society. The current paper traces how the ideas introduced in the original PNAS article evolved as they moved from the scientific into the public sphere. It presents a content analysis of the study’s depiction in five different domains of communication: the original scientific article, a press release, the traditional news media, online reader comments and blog entries. In so doing, it seeks to elucidate how the science of sex difference can influence public understandings of gender, as well as furnish insight into the dynamics of science communication in the new media environment.

### Neuroscience and sex difference

The Ingalhalikar et al. [1] PNAS paper that sparked the current analysis reported an attempt to model the neural connectivity of the brains of 949 individuals using the technique of diffusion tensor imaging. Analysis detected significant differences between the connectivity patterns of males and females: briefly, males showed proportionally greater connectivity *within* each cerebral hemisphere and females greater connectivity *across* hemispheres. The authors suggested that this difference might underpin a range of sex differences in cognitive and behavioural abilities. The methodology and results of the study are elaborated in greater detail below.

The Ingalhalikar et al. [1] study emerged in the context of rising public attention to neuroscience, which is increasingly drawn into public debate about a wide range of social issues [2]–[5]. Social scientific analyses of this cultural trend have shown that neuroscientific concepts surface particularly frequently within efforts to articulate and explain intergroup differences [4]–[8]. These discussions frequently reconstitute social categories as biological ‘kinds’. The Ingalhalikar et al. [1] study is emblematic of this tradition, seeking to identify neurobiological variation between males and females in the hope of explaining differences in their psychological and behavioural characteristics. There are several sound reasons for screening neuroscientific data for sexual differentiation, chief among them remediating the historical underrepresentation of females in biomedical research, which has disadvantaged women in respect to disease understanding and treatment [9]–[12]. However, neuroscientific research on sexual dimorphism has recently elicited intense criticism from scholars in both natural and social sciences. These critics contend that the evidence-base for many claims of sex difference is plagued by bias and methodological weakness [13]–[18].

Fine [19] has coined the term ‘neurosexism’ to describe the socio-political assumptions often embedded in the science of sex difference. Fine [19] and other critics allege that much sex difference research ultimately functions to sanction and sustain traditional gender relations. They argue that as these scientific ideas percolate through lay society, they reinforce stereotypes, reify gender binaries, legitimise the differential treatment of men and women in educational and professional contexts, and make gender inequalities appear natural and inevitable [14], [26], [20]–[23]. These posited societal repercussions are lent empirical support by social psychological research, which indicates that exposure to biological explanations of gender differences fosters greater endorsement of gender stereotypes [24], [25], stereotype-consistent behaviour [26]–[28], sexist attitudes [29], acceptance of gender inequality and support for discriminatory practices [30]. This evidence suggests that the social stakes of advances in the science of sex difference are high.

### Science, values and identity

The contention that scientific research on sex differences can be influenced by and contribute to cultural biases contradicts an idealised view of science as necessarily a force for objectivity. Many empirical studies have shown that scientific research is an intrinsically social activity, which is shaped by identity, reputation, competition, politics and financial interests [31]–[33]. Furthermore, while internally science maintains elaborate systems of ‘checks and balances’ that deliberately (though not always successfully) try to expunge personal or cultural bias, no such restrictions limit its representation in the public sphere. Indeed, the mobilisation of prevailing values and beliefs may be the key mechanism that enables lay thinkers to make sense of abstract, unfamiliar scientific information. Social representations theory, a social psychological theory that investigates how scientific ideas assimilate into ‘common sense’, finds that when people engage with scientific information, the primary concern is not a veridical rendering of scientific ‘fact’, but developing a form of knowledge that coheres with a community’s cultural projects [34], [35]. Social representations or ‘lay theories’ of science selectively reconstitute scientific information according to the ideological and pragmatic imperatives of particular social contexts [36]–[38]. As a result, the introduction of scientific ideas into public discourse is no guarantee of an impartial, classically ‘rational’ debate; indeed, the apparent neutrality of scientific concepts may make them *more* potent vehicles for ideological projects, lending socio-emotional values an ontological solidity and rhetorical force.

Much of the socio-emotional meaning that is projected onto scientific information revolves around issues of identity [39], [40]. Research shows that humans have a deep-seated motivation to justify the social system in which they live, and their cognition is moulded by the desire to construe that system as good, just and legitimate [41]. This orientation shapes public reception of scientific information, which is often absorbed into efforts to preserve existing group hierarchies. For example, Joffe’s [42]–[45] research catalogues how the impetus to bolster intergroup divides drives social representations of health and environmental risks: these risks are consistently blamed on an outgroup’s deviant, irresponsible or repugnant behaviour, which reinforces the outgroup’s stigmatisation and symbolic distance from the self/ingroup. In the domain of gender, research has found that traditional gender stereotypes are superimposed upon representations of abstract scientific information, which serves to both habituate the unfamiliar scientific content and revitalise age-old cultural understandings by affording them fresh, scientific draping. For instance, in studies investigating lay accounts of the biology of conception, gametes were personified and ascribed the stereotypical attributes of gender categories, with the sperm described as stronger, harder and more dominant than the ovum [46], [47]. These effects were strongest for individuals with more conservative sex-role orientations, which supports the proposition that people reconstruct scientific information in line with their socio-political commitments.

### Science communication in the new media environment

Bangerter [47] presents evidence that the aforementioned saturation of biological accounts of fertilisation with everyday understandings of sex roles is a gradual process, which consolidates through repeated communicative exchanges. Understanding communication processes is therefore critical in understanding how social representations of scientific information develop. Traditionally, the mass media are conceptualised as the key vessel by which scientific information moves from the laboratory into the public sphere [48]–[50]. Ideas aired in the popular media have been the target of much prior criticism of ‘neurosexism’, with the logic that the narratives purveyed to a mass audience have the greatest potential for social harm. However, scrutiny of media accounts of neurobiological sex difference has thus far taken a largely anecdotal approach to the collection and analysis of media material. Debate about popular portrayals of sex difference would benefit from a more robust empirical foundation, which systematically documents the patterns visible in media responses to scientific claims of sex difference.

Additionally, a comprehensive account of how these ideas are transmitted through society requires attention to the shifting dynamics of the new media environment. Classical models of media influence present a rather simple process whereby information is produced by science and travels via the mass media into public consciousness. This notion of a linear, unidirectional flow of information is unsustainable in the new media environment, in which audiences do not merely ingest but actively *produce* media content. Recent years have seen a decline of science coverage in the traditional media, where dedicated science sections and reporters are increasingly rare [51]. Concurrently, there has been a major expansion of science content in social media, with scientists actively utilising social media platforms to publicise and critique research [52]–[55]. While the degree of public immersion in these online debates remains unclear, surveys indicate that the internet has become the default source laypeople consult when seeking information about science [56]. Though currently internet usage varies widely across socio-demographic divides, the importance of the new media for public communication of science will continue to grow as the ‘millennial’ generation ages and as internet access widens with economic development. Expanding media analysis to incorporate new media content is therefore critical in ensuring research on public engagement with science keeps pace with contemporary society.

As yet, there has been relatively little empirical research on representations of science in social media. The research that does exist has focused primarily on Twitter, employing computer algorithms to identify patterns in large volumes of individual tweets [57]–[59]. These studies provide an expansive overview of the distribution of communicative trends across time and populations. However, the automated nature of the computational analytic strategies typically deployed, together with the 140-character limit to contributions made via Twitter, mean that the insight offered into the meanings derived of scientific concepts is often relatively superficial.

Alternative new media platforms, which afford data that is richer in content, include blog posts and the comments that readers contribute to online news articles. Blog posts are typically produced by and for communities with a vested interest in the topic at hand, and selectively focus on the aspects of the topic that resonate with those interests. Much discussion of scientific issues in the so-called ‘blogosphere’ occurs within dedicated science blogs, where individuals with high levels of scientific expertise dissect scientific research itself and its portrayal in the mass media [52], [60]. In contrast, reader comments stem from a more ‘general’ population, recording individuals’ spontaneous responses to information encountered in news websites. Research on this material has indicated that comments contain a greater diversity of content than traditional media reports, and are more likely to include moral or social judgement [61]–[64]. As such, comments may be a useful proxy for readers’ immediate, subjective responses to the scientific ideas presented in news articles. Though such content is produced by an unrepresentative minority of the population and may attract those with the most extreme perspectives, this in itself may furnish a useful indicator of the *range* of opinion on a given issue [61]. Additionally, though only small numbers of people contribute comments, their audience is much wider: research indicates that many readers of online articles also peruse the appended comments, and that this material influences their appraisal of the issues covered in the main text [65]. This electronic content may therefore provide a naturalistic complement to more traditional indices of public opinion, such as surveys and interviews.

### The case study approach

Most studies of media coverage of science amass a diverse range of texts to discern the overarching trends in how a given scientific topic is represented. For example, several recent studies have undertaken broad overviews of press coverage of neuroscience, demonstrating that neuroscientific concepts are growing in prominence, applied to a wide variety of topics, and used to advance prevailing beliefs or ideologies [4], [66]–[68]. These expansive studies offer valuable insight into the stock of frames that media outlets deploy in approaching information from a given scientific field. However, when many different scientific discussions are collapsed into a single dataset, the detail of how *specific* scientific ideas are interpreted and applied in popular contexts recedes from view. Further, restricting analysis to material from a single media platform (e.g. newspapers) affords a rather static picture of social representations of science, which does not capture how dynamics shift as the information moves between different communicative contexts.

One way of preserving this nuance is to adopt a case study approach that tracks how one scientific study evolves as it moves from its original scientific report through various media contexts. A case study design seeks to furnish an in-depth, holistic account of a single phenomenon, often by triangulating multiple sources of data [69], [70]. It is particularly adept at capturing *process*; its narrow focus means it can document direct relations between events, which can be difficult to discern with composite data [71], [72]. While focusing on a single case impedes generalisability, in-depth understanding of the dynamics of one particular case can complement and enrich understanding of the average tendencies that traverse many cases [71]. Further, one instance is sufficient to falsify proposed universalities or provide ‘proof of concept’ that a given phenomenon is possible. For example, Brossard [73] uses an instance of scientific controversy to demonstrate the porous nature of the boundaries between science and the media, with scientists actively using the media to publicise and debate research. Seale [74] highlights the self-propagating nature of media information by tracking how a single statistic in a report on physician-assisted suicide was distorted by one media report and then recited by others as ‘fact’. A detailed investigation of one particular case can therefore be a potent means of exposing the naturalistic unfolding of the processes of science communication.

### The current study

The current paper presents a case study of how representations of the Ingalhalikar et al. [1] research evolved in the month following its publication. It recruits the technique of content analysis to track how the research was construed in five domains: the original scientific article, the press release issued by the researchers’ university, the traditional news media, reader comments on online news articles, and blogs discussing the research. Importantly, the analysis does not seek to establish whether interpretations of the research are scientifically *correct*, but rather to discern the social and personal *meanings* that were extracted from the scientific information. Neither does it seek to ascribe blame for instances of bias, error or distortion, instead adopting a non-judgemental research attitude that simply catalogues the ideas that materialised in the data, without arbitrating as to their normative legitimacy [75]. This pragmatic approach best serves the research goals, which are twofold: to illuminate the process by which novel scientific information about sex differences assimilates into prevailing ‘common sense’ understandings of gender, and to shed light on the dynamics of science communication in the new media environment.

## Methodology

### Data collection

#### 1. Original scientific article

The PNAS article in which Ingalhalikar et al. [1] originally reported their results was downloaded from the journal website.

#### 2. Press release

The press release produced by the institution in which the research was conducted (University of Pennsylvania) was retrieved from the university website.

#### 3. Traditional news articles

The Nexis English language news database was searched for articles printed in the month following the publication of the PNAS article (02/12/13–02/01/14), which contained the keywords “Ingalhalikar OR Gur OR Verma OR Philadelphia OR Pennsylvania” AND “brain” AND “sex OR gender OR women OR female” (the search term incorporated just three of the authors’ surnames, which pilot research indicated were the names most frequently mentioned, because including all authors produced many irrelevant results due to the commonness of certain surnames). Results were not restricted geographically but all were written in English. Duplicated and irrelevant articles were removed from the sample, as were transcripts of television or radio shows and blog entries. The final sample included 87 articles that had been published in print newspapers or magazines, in newswires, or on the websites of established news outlets (e.g. BBC, Washington Post).

#### 4. Blogs

The same keyword query that was used in collecting the traditional news articles was entered into the Google Blogs Advanced Search function to source blogs mentioning the study, which were posted between 2 December 2013 and 2 January 2014. Results were harvested on 6 February 2014. The search engine was programmed to order results by relevance to the keywords and the search was capped at 200 results. Thirty-eight results were removed due to broken links, duplication or irrelevance, leaving a final sample of 162 blogs.

#### 5. Readers’ comments

Each article recovered in the traditional media sample was checked to ascertain whether it had an online equivalent and if so, whether it had a reader comment function. As of 23 January 2014, the online versions of 32 of the original 87 articles had comments appended. All comments that had been posted (*n* = 4,062) were copied into a text file. As the sample was very large, a random number generator was used to select 10% of the comments attached to each article for analysis. (If 10% of the comments for a particular article did not result in an integer, the figure was rounded to the nearest whole number. However, if the total number of comments attached to particular article was less than five, one comment was randomly selected for inclusion in the final sample. This ensured that each contributing article was nominally represented in the sample, even if at a higher proportion than 10%.) Automated ‘spam’ messages and empty or indecipherable comments were discarded. The final sample contained 420 individual comments.

**Figure 1:**
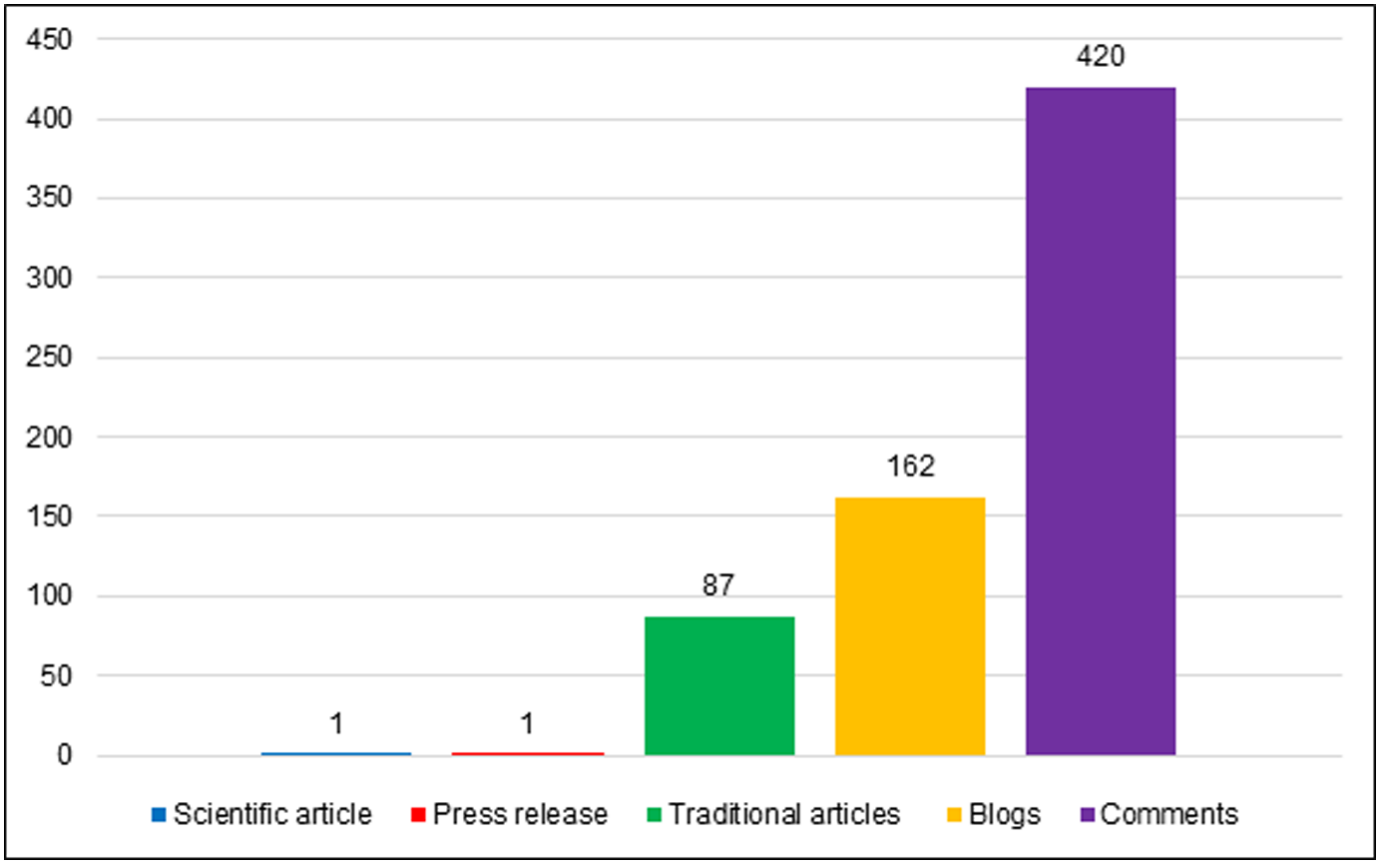
Sample size (number of data units) of each dataset.

Figure 1 demonstrates the number of data units in each corpus.

### Data analysis

All data were imported into the ATLAS.ti software programme for analysis. Data were analysed by means of content analysis, a technique for compressing large amounts of data into their analytically meaningful categories of content [76], [77]. Content analysis has previously proven a powerful tool in researching media representations of both science [50], [58], [78] and gender [79], [80]. All data were read through several times to develop a coding frame that captured the overarching features of the material. Each article, blog entry and comment was taken as a single data unit, to which multiple codes could be attached. To ensure comparability of the datasets, a common coding frame was applied to all five sources of data. The coding frame was sufficiently comprehensive that all data units could be coded with at least one code.

To evaluate the robustness of the coding frame, 20% of the data (the original article, the press release, 17 traditional articles, 32 blogs and 84 comments) was independently coded by an additional coder. These coding patterns were compared with those of the original coder using Cohen’s kappa analyses. The vast majority of codes showed good inter-coder reliability, with an average kappa value of .634 indicating ‘substantial’ agreement [81]. Codes with low reliability were modified or discarded.

After all data had been fully coded, frequency tables were produced indicating the proportion of articles or comments in which each code occurred. These frequency figures, which indicate how trends shifted as discussion moved across the different communicative contexts, are presented in the following section. It was not possible to statistically compare the code frequencies of the different datasets as the data did not meet the basic conditions for non-parametric analysis (because, for example, the original article and press release had only a single case, and the traditional articles and comments were not independent of each other). The relative frequency figures are therefore purely descriptive in nature. They supplement a qualitative account of the understandings and arguments contained within the respective code categories.

## Results

The forthcoming presentation of the results of the analysis is divided into six sections. It first presents a brief synopsis of the Ingalhalikar et al. [1] research. It then proceeds to detail how the different datasets treated (i) the suggested behavioural manifestations of the neuroconnectivity difference, (ii) the causality of the reported sex difference, (iii) the conceptual and linguistic framing of the ‘difference’ concept, (iv) the differential valuation of men and women, and (v) the findings’ relations to the gender politics of contemporary society.

The latter five sections will each commence with a graph depicting the relative prevalence of codes in the five datasets, followed by a qualitative account of the relevant material. When considering the proportions depicted in the frequency graphs, the unique contingencies of the five datasets should be kept in mind. As the original scientific article and press release had only one data unit, a code’s involvement in these data-sources can only be tabulated according to its presence (i.e. 100% prevalence) or absence (0% prevalence). In addition, the proportion figures for the comments data are typically lower than those for the blog or traditional media data, because individual comments were shorter and therefore contained fewer codes. Due to these discrepancies between the datasets, the code prevalence figures they reveal are not directly comparable. The graphs are therefore not intended to facilitate direct numerical comparisons, but to complement the qualitative analysis of the data by schematising how topics drifted in and out of focus between the various media contexts.

### Synopsis of the scientific article

The Ingalhalikar et al. [1] paper described a study conducted by 10 researchers from the University of Pennsylvania and the Children’s Hospital of Philadelphia. Using diffusion tensor imaging, a technique that facilitates the visualisation of anatomical connections between different areas of the brain, the research modelled the structural connectomes (maps of the neural connections that traverse the brain) of the brains of 949 individuals aged between 8–22 years. Statistical analysis detected significant differences between the connectivity patterns of male and female participants. Males showed greater within-hemispheric connectivity and females greater between-hemispheric connectivity in all regions studied except for the cerebellum (a region involved in motor control), where the pattern was reversed. Sex differences were least pronounced in the youngest participants, which the authors interpreted as evidence of a divergence in the developmental trajectory of male and female brains during adolescence. Though the research did not collect any cognitive or behavioural data, the authors suggested that males’ greater within-hemispheric connectivity would link perception to action, conferring “*an efficient system for coordinated action*”, while females’ greater inter-hemispheric connectivity “*would facilitate integration of the analytical and sequential reasoning modes of the left hemisphere with the spatial, intuitive processing of information of the right hemisphere*”. They also speculated that the neuroconnectivity differences might underlie several cognitive and behavioural sex differences that their research team had detected in previous studies, though they did not present any statistical tests of the relationship between the neuroimaging data and these behavioural measures. The authors characterised their data as revealing “*fundamental sex differences in the structural architecture of the human brain*”. This, they argued, explains the phenomenon of “*adaptive complementarity*”, whereby males and females are endowed with distinct cognitive skills that suit them to divergent behavioural and social functions.

Having summarised the key features of the source article, the paper now moves on to elaborate the meanings that were derived of this scientific information across the different datasets. Note that the quotes provided throughout this section are identified in terms of the dataset from which they derive (PR  =  Press Release; T  =  Traditional media; B  =  Blogs; C  =  Comments) and the number assigned to that data unit in the relevant dataset (as recorded in the Supporting Information Files S1–S3). All quotes are reprinted verbatim without correction of spelling or grammatical errors.

#### 1. What are the behavioural manifestations of the sex difference in neuroconnectivity?

**Figure 2:**
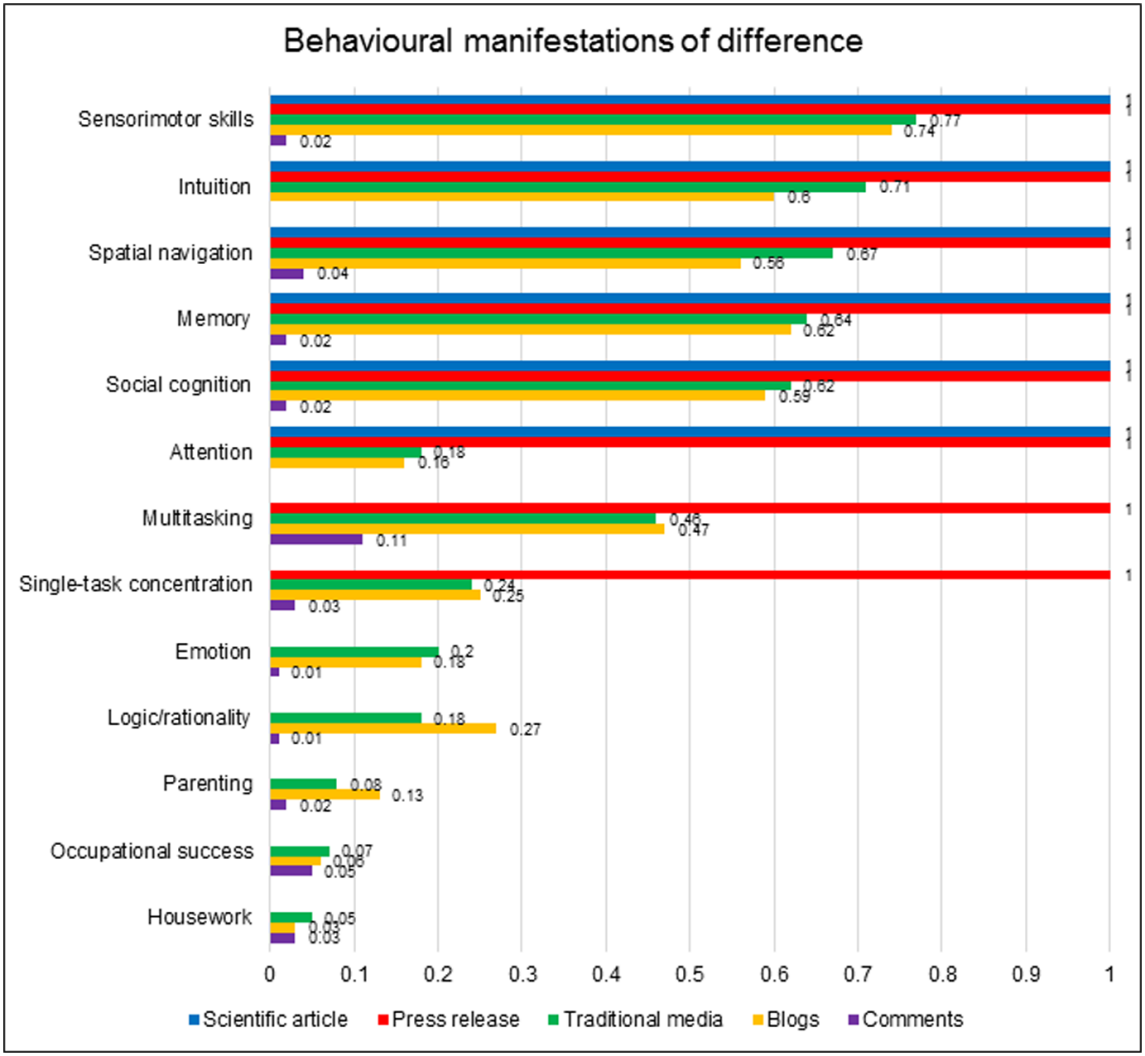
Prevalence of reference to the various behavioural domains across the datasets.

In both academic and popular contexts, a primary way in which the posited neural sex difference was made meaningful was via speculation about its functional effects. Could this difference between male and female brains explain sex differences in behaviour, emotion or cognition? Figure 2 displays the various behavioural domains that were suggested to show a sex difference that this research might explain. It records the proportion of data units from each dataset that mentioned the topics.

Though no behavioural data were directly reported in the Ingalhalikar et al. [1] paper, it mentioned that previous research had identified six functional domains – sensorimotor skills, spatial navigation, intuition, memory, social cognition and attention – as loci of sex differentiation, with men showing greater affinity for sensorimotor and spatial cognition and the remaining four functions domains of female superiority. All six functions were carried through to the press release, and most retained their presence in the majority of popular articles. The exception to this was attention, whose prevalence in blogs and traditional articles was much lower than the other five functions. This may reflect the design of the press release, which mentioned attention relatively late in the text; media coverage may have relied disproportionately on the earlier paragraphs.

Perhaps more interesting than the behavioural domains that were mentioned in both scientific and popular contexts are those that were introduced anew in the popular media, without precedent in the original research paper. These show the media spontaneously projecting existing gender scripts onto the novel scientific information. Particularly salient in this regard were the two faculties of ‘multitasking’ and ‘single-task concentration’. These were often positioned as antithetical talents, with women cast as more competent at the former and men the latter. Though neither function was explicitly mentioned in the PNAS article, both were introduced in the press release in a sentence that was frequently reproduced in popular articles:

*on average, men are more likely better at learning and performing a single task at hand, like cycling or navigating directions, whereas women have superior memory and social cognition skills, making them more equipped for multitasking and creating solutions that work for a group. [PR]*

The press release’s claim that the results underpinned a female affinity for multitasking developed into a major focus for subsequent media coverage, monopolising the headlines of several traditional and blog articles. The implication that this was the ‘take-home’ message of the research is interesting, given that the researchers did not test multitasking abilities or indeed mention the concept in the PNAS report. Nevertheless, media articles hailed the advent of scientific ‘proof’ of an aptitude that has long been obvious to women themselves.

*Women have known it for generations - and the proof has finally arrived. Scientists have found that the female brain is “hard-wired” to be better at multi-tasking. [T61]*

Multitasking was the behavioural faculty that received most attention in the comments data. Reading a news article that referenced multitasking prompted female commenters to contemplate their personal experience of balancing personal, professional and domestic responsibilities, while males made jokes about their own attempts to juggle different tasks. The comments also showed a persistent trend wherein certain commenters would react to the suggestion of female superiority in multitasking by reconstituting multitasking as a negative attribute. These comments contended that distributed attention ultimately results in substandard performance, and argued that single-minded concentration was the more valuable skill.

*You could argue that women are incapable of focusing on the job at hand---multi tasking often being a euphemism for never being able to complete anything. [C46∶10]*

Thus, despite multitasking’s absence from the original scientific paper, it was introduced in the press release and found major currency in the popular media and comments.

The data also revealed a number of behavioural domains that were introduced exclusively in the popular media contexts, independently of any reference in either the press release or original article. Particularly salient among these was the dialectical pair of emotion and rationality. The finding of sex difference in connectivity was interpreted with reference to notions of hemispheric lateralisation, which delegated emotion to the right and logic to the left hemisphere of the brain. Within this framework, women’s greater inter-hemispheric connectivity implied that their thought process was more integrative of emotion, whereas the structural independence of men’s hemispheres produced a compartmentalisation of emotional and rational thought. Via such interpretations, newspapers, blogs and comments absorbed the research into a polarity that positioned women as fully emotional beings, and men as purveyors of pure rationality.

*They are saying that women are more emotional thinkers on average and men tend to be more fact-based thinkers. [C64∶113]*

Finally, the popular media also departed from the scientific article and press release in relating the research to the social distribution of labour. Articles and comments periodically suggested that the posited brain difference may explain women’s supposedly better parenting skills and the gendered division of domestic chores. Some of the attention to parenting could be traced to a quote attributed to one of the study authors, Ragini Verma, in which she claimed that, “w*omen tend to be better than men at these kinds of skill which are linked with being good mothers*” *[T83]*. This quote was originally printed in Britain’s *Independent* newspaper (which carried the story on its front page) and was subsequently reproduced by several other news outlets. The focus on parenting in the popular press was therefore partly fuelled by interpretations offered by the researchers themselves. However, housework received no such leverage, and yet was mentioned in 5% traditional articles, 3% blogs and 3% comments. These data usually enlisted the research to claim that women are ‘wired’ to notice and remediate household disarray, with men laughingly dismissed as ineffective contributors to domestic labour.

*Whereas the male brain is more wired for navigating outdoor activities, such as hunting woolly mammoths, the female brain is wired to notice more sensory detail. Men are less likely to notice dust, which, women tell me, is a mix of fine particles that settle on furniture. [T11]*

In some corners of the data, men’s domestic failings were counterbalanced by their ‘breadwinning’ occupational role: 7% traditional articles, 6% blogs and 5% comments attributed sex differences in occupational achievement to neural inheritance. The traditional press resisted a presumption that men invariably triumphed in occupational domains, frequently arguing that women’s aptitude for multitasking and emotional intelligence suited them for leadership roles. In the comments, however, this shifted into a clear privileging of male occupational achievement. Particularly salient in the comments was the repeated appearance of provocative statements that women have a poor track record in ‘inventions’ or receipt of Nobel prizes, with the assumption that this reflected biologically-ordained inferiority.

*C’mon Ladies, much as I love you all lets face facts. Men invented piratically everything you use and enjoy. The Telephone, The Computer, The Jet Engine, The Train, the Motor Car, Etc Etc the list is endless. Without us you would still be scratching around in caves so lets have no more of this nonsense and concentrate on your hand bags [C51∶3]*

In summary, all sex-differentiated behavioural domains mentioned in the original article were carried through to the press release and popular media. However, the popular media expanded the scope of discussion by relating the research to behavioural domains that were not mentioned in the scientific article. The press release introduced the facilities of multi-tasking and single-task concentration, and these topics were enthusiastically adopted by popular articles and comments. Moreover, even without prompting by the press release, newspapers, blogs and comments acted autonomously to project prevailing understandings of gender differences – notably the emotion-rationality dualism and traditional role-divisions in domestic and occupational labour – onto their interpretations of the research findings.

#### 2. What causes sex difference?

**Figure 3:**
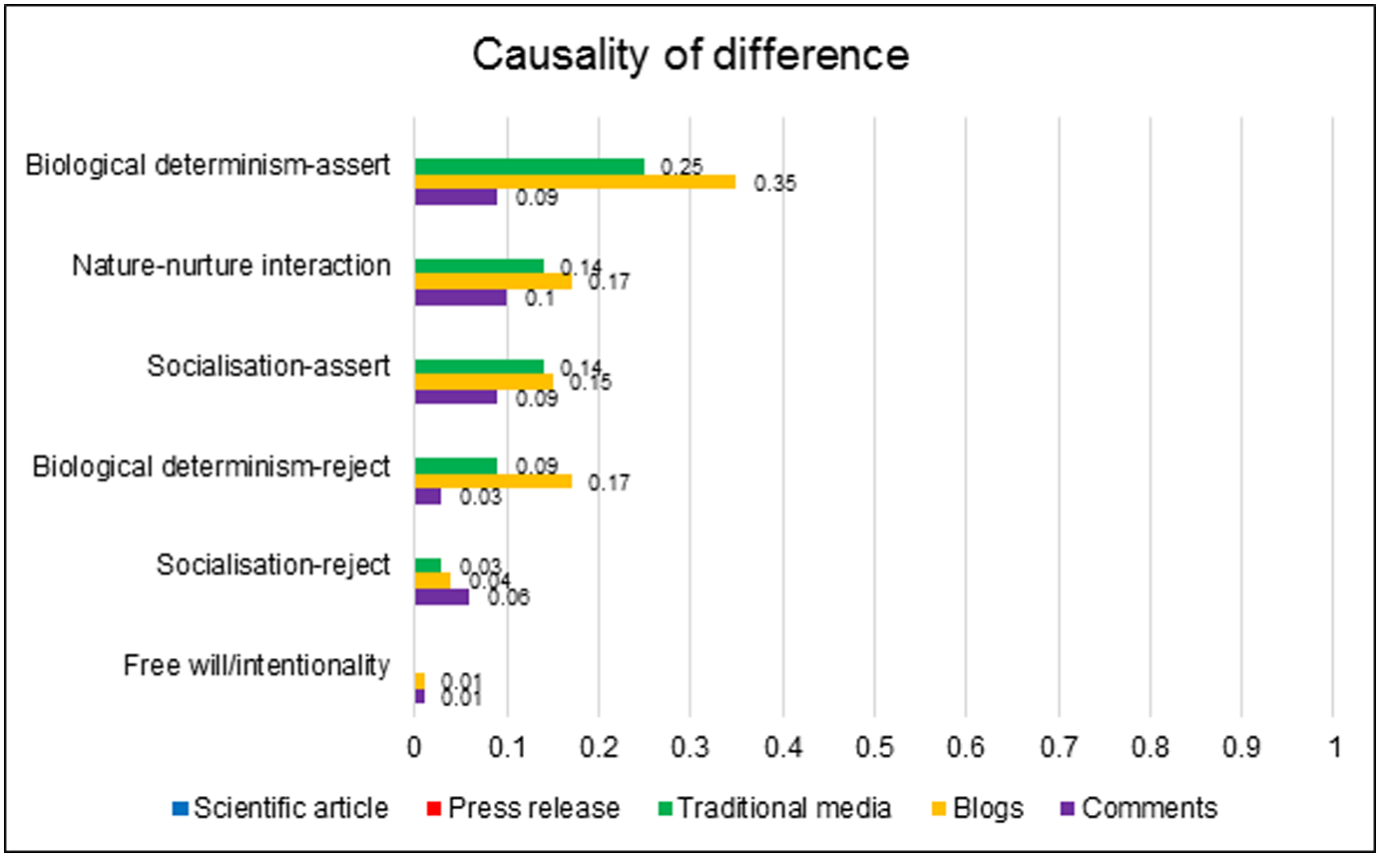
Prevalence of causal attributions for sex difference across the datasets.

No data were identified that denied the premise that differences existed between male and female brains. However, in the popular media there was considerable debate regarding what *caused* the anatomical differences identified by Ingalhalikar et al. [1]. Figure 3 illustrates the attributional patterns that were detected in the data.

Where a clear causal statement was produced, the attribution was usually to biology. Assertions of biological determination of sex differences occurred in one-quarter of traditional articles, over one-third of blogs and almost one-tenth of comments. The metaphor of ‘hard-wiring’ was frequently employed, conveying that sex-typed behaviour is natural and immutable.

*Scientists have found that the female brain is ‘hard-wired’ to be better at multi-tasking. Men’s brains, in comparison, are better at concentrating on single complex tasks - whether it be reading a map or cooking a meal. [T61]*

However, this stress on biological causality was far from absolute. In the traditional and blog data, reference to the causal power of socialisation also occurred in a sizeable minority of articles (14% and 15% respectively). Parenting, education and cultural expectations were among the social factors implicated in producing sex differences in behavioural and emotional tendencies.

*Males develop improved spatial skills not because of an innate superiority but because they are expected and encouraged to be strong at sport, which requires expertise at catching and throwing. Similarly, it is anticipated that girls will be more emotional and talkative, and so their verbal skills are emphasised by teachers and parents. [T19]*

Though attributions to social factors were relatively common in the traditional and blog articles, in frequency terms they were overshadowed by reference to biological causality. However, this imbalance disappeared in the comments data, which afforded equal emphasis to biology and socialisation. The comments were often embedded in a dialogical framework that positioned biological and cultural influence as conflicting explanations, and contained extended debates between commenters regarding the relative influence of each.

*I think it has a lot more to do with upbringing and the pushing of gender roles on children from an early age. If it were caused by something as rigid and factual as brain structure, why would there be so many exceptions to these rules? [C9∶2]*

*You suggest that the observed differences are the result of sexually dimorphic activities, yet what causes that divergence? It’s not simply cultural exposure. And even if it was, where, evolutionarily, did the cultural sexual dimorphism come from, except from differences in biology? [C1∶1]*

These aspects of the data demonstrate the continued relevance of the nature-nurture debate within lay society. Assertions of biological causality were accompanied by rejections of socialisation, and vice versa. However, it is also important to note the steady presence of statements conveying a belief that nature and nurture *interact* in the formation of sexual identities. Reference to a biology-socialisation interaction materialised in 14% traditional articles, 17% blogs and 10% comments. This sometimes included direct reference to the scientific concept of neuroplasticity, particularly within the more specialist science blogs. Though Ingalhalikar et al. [1] did not offer this interpretation in the PNAS article, their finding that connectivity differences were stronger in older than younger cohorts was often recruited in support of a nature-nurture interaction, cast as evidence that social experience imprints itself on the brain.

*Male and female brains showed few differences in connectivity up to the age of 13, but became more differentiated in 14- to 17-year-olds. So basically male and female brains start out the same, but social conditioning of behaviours leads to differences in the brain - because learning something changes the brain. [C64∶78]*

Thus, the data revealed media sensitivity to the *interactions* between various causal factors: though biology was positioned as the proximal cause of gender-typed behaviour, these biological characteristics could be conceptualised as socially formed.

#### 3. The framing of difference

**Figure 4:**
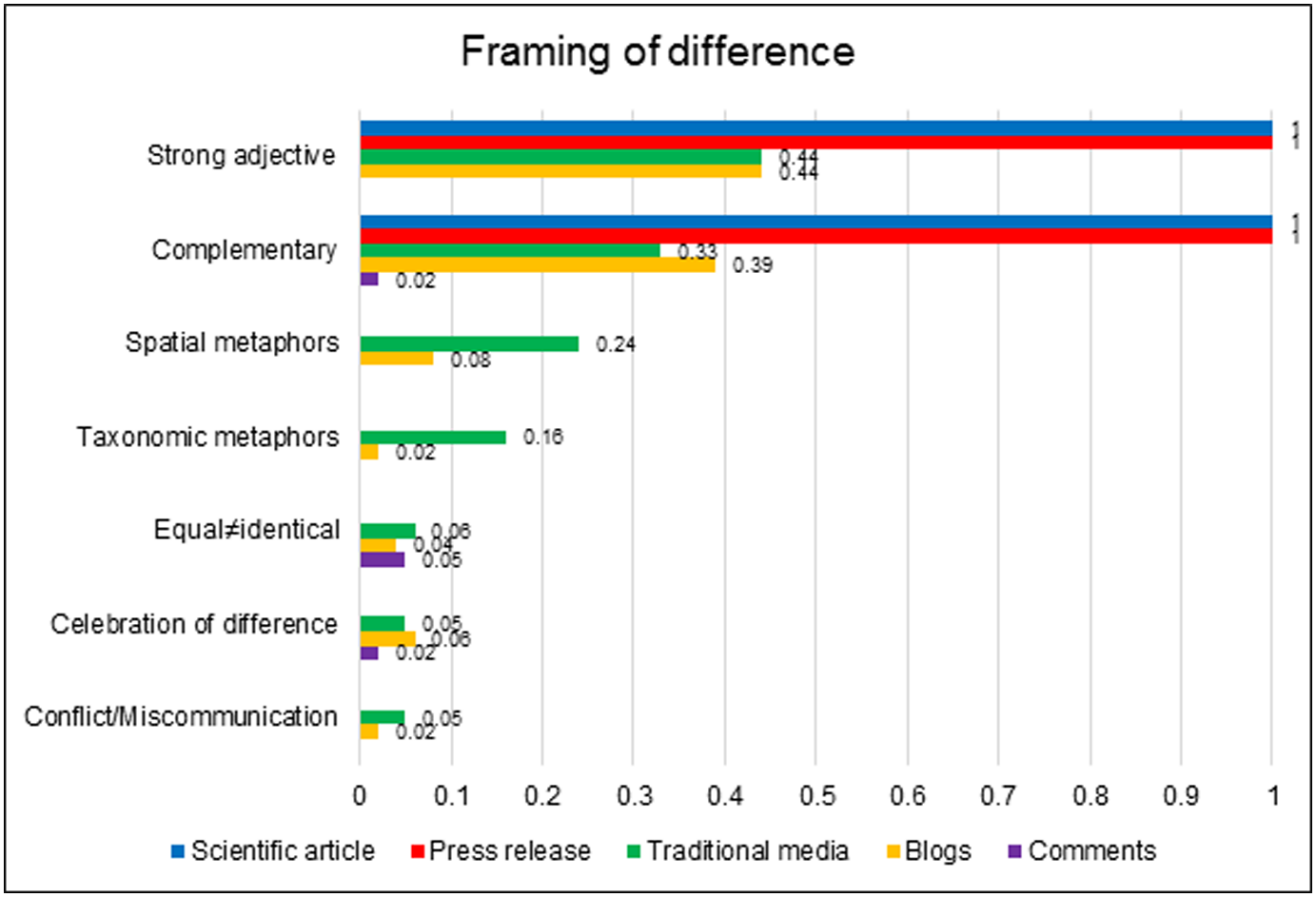
Prevalence of the various modes of framing difference across the datasets.

In much coverage of the Ingalhalikar et al. [1] research, it was apparent that the precise ways in which the sexes differed was secondary to the ‘proof’ that they *were* different. The unspecific concept of *difference* was meaningful in itself, independently of any explication of where exactly that difference lay. Figure 4 catalogues how the idea of difference was conceptually and linguistically framed in the data.

Firstly, a pattern that was prominent throughout the data was specific attention to the *extent* or strength of difference. In the scientific article and press release, and in almost half of traditional and blog articles, men and women’s brains were not merely ‘different’: they were ‘starkly’, ‘completely’ or ‘fundamentally’ different. These strong adjectives constituted the gap between the sexes as profound. This gap was further stressed through use of metaphor, which was a particularly salient feature of traditional media dialogue. Almost one-quarter of traditional articles employed metaphors that positioned men and women as spatially distant – ‘poles apart’ or ‘on different planets’, or via the oft-repeated cliché ‘men are from Mars, women from Venus’. Another metaphorical pattern, present in one-sixth of traditional articles, drew a taxonomic separation between the sexes, portraying them as ‘different species’.

*The differences between the genders were so profound that men and women might almost be separate species. [B16]*

In considering the study’s implications for interpersonal relations, the purported sex differences were generally portrayed as producing harmonious inter-sex relationships. This perspective was firmly instantiated in the original scientific article, which cast the observed connectivity differences as a demonstration of inter-sex ‘complementarity’. The characterisation of sex differences as complementary resurfaced in the press release and in over one-third of news articles and blogs - far exceeding the attention afforded to the prospect that sex differences could produce inter-sex conflict or miscommunication, which was mentioned in just 5% newspaper and 2% blog articles. The data posited that a *combination* of male and female brains produced a formidable team, with each sex’s unique talents compensating for the other’s weaknesses. Difference was thereby cast as a positive phenomenon that merited celebration.

*men and women are different, and we should celebrate our differences rather than pretend they are not so. [T11]*

Numerous writers pointed out that difference in specific skills did not connote difference in global worth, and explicitly dissociated the concepts of equality and sameness. Arguments that personal attributes can be different, but equally valued represented an attempt to reconcile the research with the principle of gender equality.

*We can be equal without having to be identical. [C19∶7]*

However, despite this nominal affirmation of the ideal of equality, parity of esteem was deployed rather selectively within discussion of male-female difference. The posited ‘equal but different’ dispositions positioned men and women in firmly traditional sex roles, with women the emotional, empathic carers and men the single-minded, rational breadwinners. Little data considered whether choices that transgressed these biologically-grounded role divisions might merit equal respect. Additionally, in a handful of blogs and comments, the concept of sexual complementarity was recruited into debate on same-sex unions. Several blogs written for religious or politically conservative audiences seized on the researchers’ use of the term ‘complementarity’ to cast homosexual relationships as intrinsically deficient, and unsuitable contexts for rearing children.

*Using science to help the world better understand how man and woman are equal and yet different, as opposed to equal and therefore interchangeable in role and function, has far reaching implications. Not least because it adds strength to the Catholic claim that the complementary differences between men and women, when combined together in love, are essential to the true definition of marriage. The different brains of men and women leading them to bring different gifts to this unique procreative union. This research is welcome then for it helps us better understand the different roles mothers and fathers play in the development of the young. [B143]*

Thus, most commentary constituted sex difference as profound and celebrated this as a positive dimension of human relationships. However, in certain corners of the data this legitimised the marginalisation of individuals or families who did not accord with traditional sex-role divides.

#### 4. Differential valuation of the two sexes

**Figure 5:**
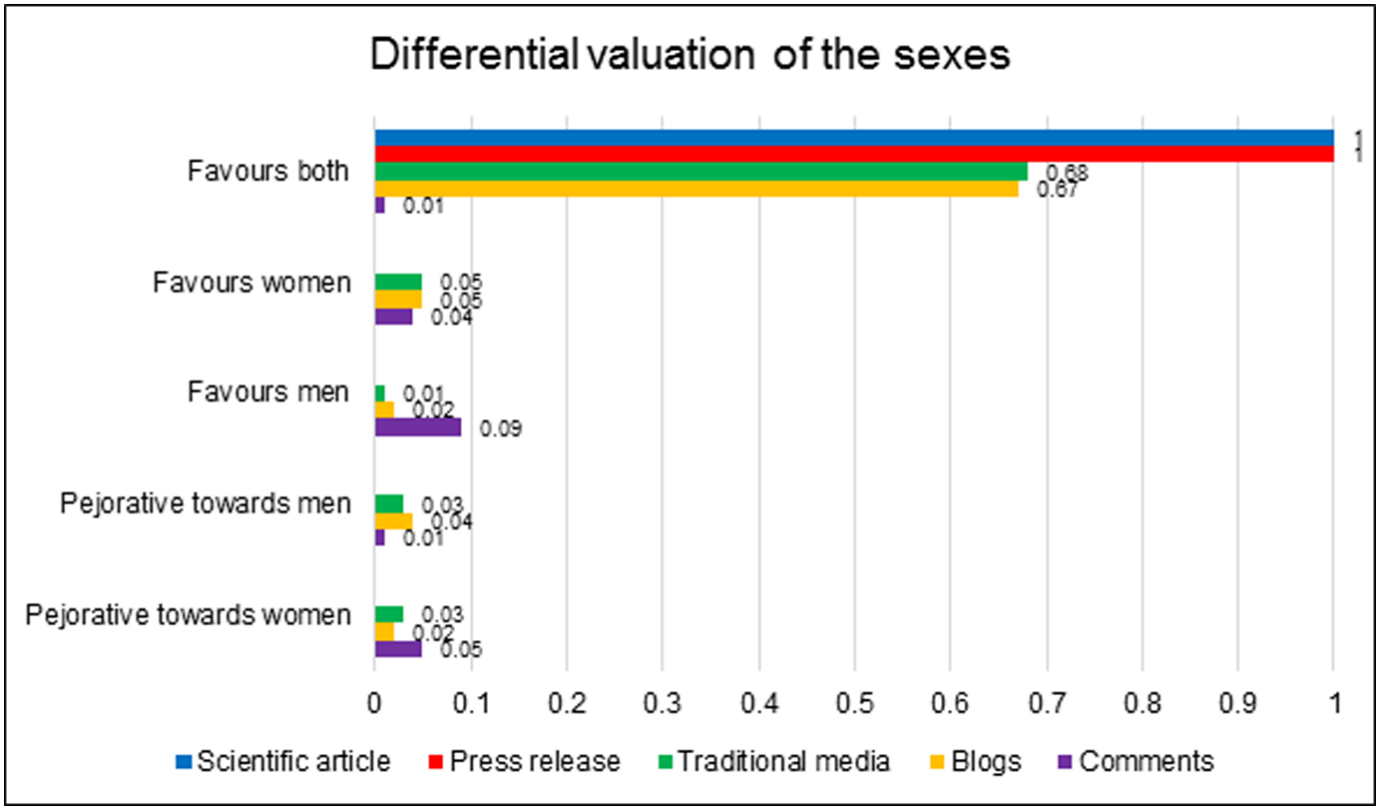
Prevalence of instances of differential valuation of the sexes across the datasets.

It might be expected that the proposition that male and female brains were different would prompt questions about which was ‘better’. Figure 5 collates the instances in which privilege was granted to one sex.

In accordance with the aforementioned principle that both sexes’ distinctive abilities were equally valuable, most of the data refrained from positioning one sex as superior. The most consistent, ‘default’ perspective was to portray the research as casting *both* sexes in a favourable light. The scientific article, press release, and most traditional and blog articles were careful to point out that men and women each have areas in which they excel.

*males were more inclined to excel at completing one single-focused job, while females were more apt to multi-task. Thus, the idea of males being superior navigators and directors, while women excel in the areas of social competency and memory-retention may actually be rooted in scientific principles. [B149]*

However, these dynamics shifted in the comments. Firstly, comments were more likely than the other datasets to express a preference for one sex over the other. This occurred in 13% comments, as opposed to 6% traditional articles and 7% blogs, while only 1% comments adopted the standard media perspective that the research complimented both sexes. Secondly, on the rare occasions when traditional and blog articles did privilege one sex, it was more likely to be women: 5% traditional and 5% blog articles favoured women, as opposed to 1% traditional and 2% blog articles who favoured men. However, when comments expressed a preference, it was usually to the advantage of the male sex: 9% of comments clearly privileged men, relative to the 4% who favoured women. Numerous commenters objected to the positing of female advantage in particular skills and left comments defending male superiority.

*the fact is that men are performing better than women in each and every field in reality especially in India inspite of the fact that women get all sorts of facilities and reservations in India. So men are much superior to women whether it is single- tasking or multi- tasking.These research do not mean anything in reality. they are just for time pass to make women feel good and proud. [C47∶1]*

Further reinforcing the more partisan nature of the comments was their inclusion of overtly pejorative statements towards one or the other sex. While overall prevalence of derogatory statements was fairly similar between the comments, blogs and traditional articles (around 6%), when broken down between insults levelled at men and women, the data reproduced the patterns visible for expressions of preference towards one sex. The pejorative statements present in the traditional and blog data were usually directed towards men, and were generally packaged in a light-hearted or ironic tone. In the comments, pejorative statements were almost entirely directed towards women, and the language was more hostile than the jokes that occurred at men’s expense.

*What about PMS, when a woman can become a complete and utter 2@? [C9∶3]*

In summary, while the vast majority of data was careful not to privilege one sex over the other, the comments were more prone to favouritism towards one sex, usually men.

#### 5. Gender politics

**Figure 6:**
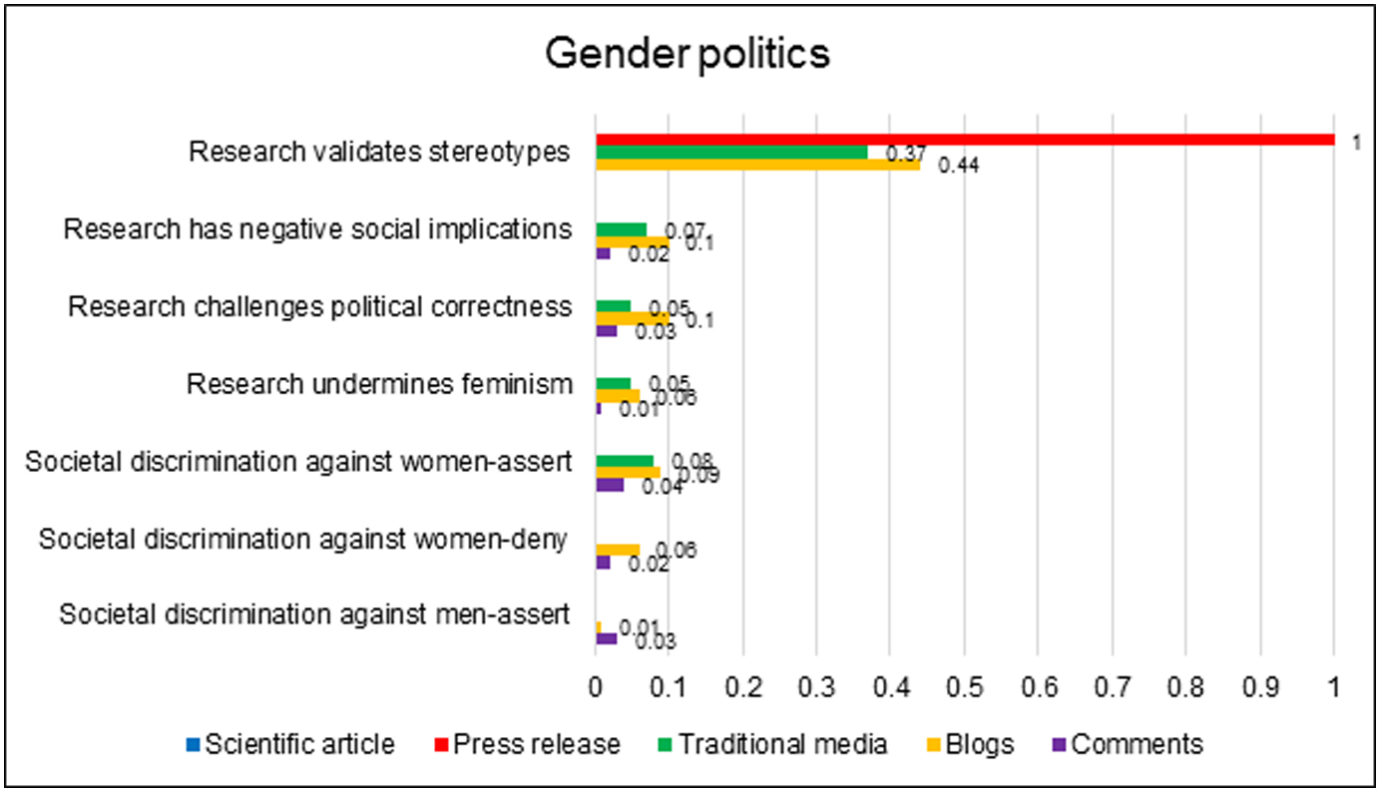
Prevalence of reference to various dimensions of gender politics across the datasets.

Notably, the research was not approached as a neutral, detached instance of scientific inquiry; it was made meaningful by embedding it in its wider societal context. Figure 6 schematises the ways in which the media related the research findings to the gender politics of contemporary society.

Throughout the data, the research was represented as a vindication of the factual truth and normative legitimacy of established gender stereotypes. Such statements occurred in the first sentence of the press release (“*A new brain connectivity study from Penn Medicine […] found striking differences in the neural wiring of men and women that’s lending credence to some commonly-held beliefs about their behavior” [PR]*) and in over one third of traditional and nearly half of blog articles. Though the original PNAS article contained no direct reference to stereotyping, journalists obtained quotes remarking on the data’s correspondence with cultural stereotypes from two of the researchers, Ragini Verma (*“I was surprised that it matched a lot of the stereotypes that we think we have in our heads” [T64]*) and Ruben Gur (*“‘As much as we hate stereotypes,’ Prof. Gur said, ‘a lot of them have some kernel of truth in them’” [T52]*). The depiction of stereotypes that thereby emerged marginalised the role of history, cultural institutions or individual bias: stereotypes simply originated in material fact.

*Science has now proved that the male brain and the female brain are wired differently. What are blithely called sexual stereotypes have a basis in neuroanatomy. [B51]*

In 10% blogs, 5% traditional articles and 3% comments, this validation of stereotypical sex differences was heralded as a welcome corrective to so-called ‘political correctness’. The latter term was usually used in a dismissive way to signify a socially powerful ideology, enforced by a ‘liberal elite’, which forbade the acknowledgement of any difference between the sexes. Its critics harnessed the epistemic authority of science to depict political correctness as a wilful denial of reality.

*Scientists are just beginning to trace the connections between genes, brains, and life trajectories. It is still politically fashionable to deny gender and population differences in cognition. But then, cold reality has always been a different kettle of fish than political correctness. [B41]*

As well as posing a challenge to political correctness, the research was also intermittently characterised as a repudiation of feminist ideals. When feminism was mentioned, it was usually in a markedly negative tone; indeed, only one blog and two comments made an explicitly supportive statement regarding feminism. Particularly in blogs, the language used when speaking of feminists was often derogatory, portraying them as deluded or irrational and dubbing them *“fembots” [B153]*, *“obnoxious whining feminist cranks” [B68]* and *“tedious bores” [B154]*. Numerous bloggers and commenters believed that feminist theory insisted that men and women are biologically identical, and expected that this research would therefore spark a *“feminist outcry” [T35]*. They welcomed the disruption they believed this research would pose to feminist agendas.

It is important, however, to also highlight the pockets of data that objected to these socially conservative interpretations of the research. A small but consistent strand of argument, which was mostly aired within blogs, expressed unease about the social implications of the research, particularly its potential to perpetuate gender stereotypes and inequalities. Critics worried that the PNAS article and its popular interpretations would function as a form of self-fulfilling prophecy, shaping expectations of gender-typical behaviour to which individuals and institutions would gradually adapt.

*Every “women are intrinsically worse at [numeracy/spatial skills/science/intense focus]” story contributes to the systematic discrimination against them in technical fields, and every “men are intrinsically worse at [communicating/emotional literacy/relationships]” story lowers the bar for acceptance of bad behaviour from men. [B47]*

These data displayed sensitivity to issues of gender inequality, with 9% blogs, 8% traditional articles and 4% comments mentioning historical or current discrimination against women. However, it should be noted that the positing of systematic discrimination against women was not uncontroversial: 6% blogs and 2% comments explicitly denied that women faced discrimination, while 3% of comments asserted that a disproportionate focus on remediating female disadvantage effectively amounted to discrimination against men.

*with the new double standard, only women are allowed to have superior abilities, not men. You see this constantly in films, television, the press, everywhere. [C87∶34]*

In summary, the research was not seen as arcane scientific information, but as a discovery with direct repercussions for gender identities and relations. It validated abiding sex stereotypes and was drawn into ongoing disputes between different cultural and ideological communities. It also catalysed debate about social issues external to the research itself, such as patterns of discrimination against men and women.

## Discussion

This analysis tracked the journey of one high-profile study of neurobiological sex differences from its scientific publication through various layers of the public domain. It adopted an innovative empirical approach, which combined multiple sources of scientific, traditional and new media data to capture how dialogue about the Ingalhalikar et al. [1] study unfolded in the month following its publication. The analysis showed that scientific research on sex difference is embedded within the wider terrain of gender politics, and illustrated how scientific claims can be absorbed into the social psychological processes that sustain gender stereotypes, norms and values. It also furnished an original insight into the dynamics of science communication in the contemporary media environment, demonstrating how media representations are diversified by the involvement of new media outlets, which broaden the range of agents who can impose their cultural agendas and conceptual frameworks onto the scientific information. Scientific information is thereby consolidated as a form of social knowledge that wields direct implications for understanding self, others and society.

### How do science, gender and media intersect in contemporary society?

The data as a whole illuminate the process by which meaning was progressively derived from the premise that male and female brains show anatomical differences. The dispassionate terminology with which the identified sex differences were interpreted in the original scientific article (e.g. “*coordinated action*”, “*integration of the analytical and sequential reasoning […] with the spatial, intuitive processing of information*”) was transformed in the press release into terms that resonated with abiding gender stereotypes (“*navigating direction*”, “*multitasking*”). Despite the absence of any cognitive or behavioural data in the Ingalhalikar et al. [1] paper, the traditional media rendered these behavioural phenomena the primary focus of the research study. The comments and blogs then set about contextualising these biologically-grounded behavioural differences in relation to personal and community experience. The journey of information from scientific journal through the various layers of public reception was characterised by the evolution of increasingly diversified, personalised and politicised meaning.

In discussing the saturation of scientific knowledge with personal and cultural meaning, it is important to avoid a framework that sets ‘pure’ science against a contaminated public sphere [35], [82], [83]. Sex difference research is initiated, funded and published in a society that deems it interesting and/or valuable, and the data produced are interpreted with reference to the gender dynamics of that society. While appraisal of the technical elements of Ingalhalikar et al.’s [1] research is outside the scope of this paper, it is worth noting that several aspects of their written account extrapolated beyond the information that their data strictly communicated. These include the description of sex differences as “*fundamental*”, the assertion that the connectivity differences underpin an inter-sex “*complementarity*”, and speculation about the functional effects of these neural differences, despite the lack of correlating behavioural data. Additionally, it is notable that some features of media coverage, which outwardly appear to depart from the original scientific information, were fuelled by quotes that the researchers themselves apparently provided to journalists (for example, regarding the results’ correspondence with traditional stereotypes or implications for parenting ability). Previous research has also implicated scientists’ informal communications with journalists in the interpretative leaps that characterise some media coverage [84]. This accords with Brossard’s [73] depiction of the porous boundaries between science and society: scientists are also citizens of a society, and the social currency of their research depends on its resonance with cultural categories and values.

The press release was a particularly important site for articulating the study’s relation to societal interests. Consistent with previous research [85]–[88], the analysis suggested that the press release was pivotal in shaping the foci and framing of subsequent media coverage, as it was often journalists’ sole source of information about the study. This meant that information that was lost between the scientific article and its press release rarely resurfaced in subsequent discussion of the study, while topics that were newly introduced in the press release (e.g. multitasking) could develop into focal points of media accounts of the research. Crucially, the very first sentence of the press release established that the core significance of the research was that it “*lend[s] credence to some commonly-held beliefs about [men and women’s] behavior*”. This construal of the research as a vindication of gender stereotypes became a dominant frame for much media commentary. For those involved in public communication of science, it may be important to know that the press release can be a ‘point of no return’ in the evolution of social representations of a research study.

However, despite the press release’s importance in cuing particular interpretations, it did not entirely constrain the range of meanings offered by the popular media. The data showed that in making sense of this new study, the media cultivated entirely original readings of the results, for example relating them to gendered divisions of labour. The data therefore provide a naturalistic analogue for previous experimental findings that prevailing gender stereotypes are spontaneously projected onto abstract scientific information [46], [47]. Scientific developments in the biology of sex provide an opportunity to rehearse abiding cultural understandings of gender identities, even if the research itself contributes no information about the dimension of identity in question.

As well as elaborating the characteristics of within-group identities, scientific research on sex difference resonates with the psychological impulse to consolidate the boundaries *between* social categories. In accordance with previous research showing that scientific knowledge can be deployed to fortify intergroup divides [42], the current data revealed enthusiastic reception for the premise that men and women are fundamentally different ‘kinds’ of person. Underlining the sheer fact of difference often took precedence over elucidating the precise ways in which that difference manifested, and the breadth of difference was accentuated through dramatic vocabulary and metaphors. This stress on categorical difference fuelled a strictly binary construal of gender, which marginalised individuals whose identity or behaviour might transgress this dichotomy.

Research in psychological essentialism indicates that such striving for discrete, impermeable category boundaries often accompanies the stigmatisation of one category, whose disfavoured traits are constituted as intrinsic, natural and inevitable [89], [90]. As such, it might be expected that the demonstration that male and female brains are different would spark aspersions about the inferiority of one brain ‘type’. Here, there were striking stylistic differences between the different data-sources. The traditional media typically oriented toward a tactful, diplomatic tone, carefully refraining from allusions to the superiority of one gender. On the rare occasions when the traditional media did privilege one gender, it was more likely to be women, reflecting sensitivity to a cultural context in which discrimination against women is more heavily proscribed. However, this preferential emphasis on female talents sometimes triggered a backlash in the comments, which would accuse the media of anti-male bias and attempt to devalue the alleged manifestations of female superiority (e.g. in casting multitasking as an inefficient, undisciplined strategy). As a source of data, comments were unadulterated by the political delicacy that constrained the traditional media and (to some extent) blogs, and exposed a latent misogyny that continues to mark public reception of scientific information about sex difference.

However, despite the relatively stronger presence of sexual animosity in the comments data, this still characterised only a small minority of comments. It is important to emphasise that across the data as a whole, the predominant message taken from the research was that neural sex differences made for complementary behavioural tendencies, with most data assiduously framing men and women’s unique abilities as equally valuable. In casting the sexes as ‘different but equal’, writers explicitly invoked egalitarian principles (even while simultaneously making disparaging remarks about feminism). While this attests to a widespread deference to the ideal of gender equality, such nominal endorsement of egalitarian values does not necessarily signify genuine parity of esteem. Social psychological research shows that despite widespread opprobrium of gender discrimination, sexist attitudes persist in contemporary society, albeit in more subtle forms. Modern sexism is primarily distinguished by its *benevolent* tone, manifesting, for example, in praise of stereotypically ‘feminine’ traits such as warmth or kindness [91], [92]. Though such ascriptions are superficially positive, they communicate restrictive role-norms and legitimise the devaluation of women’s ability in other, more socially valued trait-domains. In particular, women’s advantage in social-emotional traits often comes at the expense of their perceived competence or agency, which justifies their exclusion from socially powerful positions [93].

The characteristics ascribed to men and women in the Ingalhalikar et al. [1] paper and its media coverage tended to correspond with these patterns of stereotype content. Men were portrayed as logical, focused and physically competent actors, while women’s strengths lay in emotional intelligence, social skills and caring. A possible exception to this were the memory and attention skills that purportedly befitted women to multitasking. However, though the traditional media and blogs mostly construed multitasking as a cognitive asset, in the comments it was frequently dismissed as a fruitless or trivial facility. For certain lay populations, ‘multitasking’ connoted haphazard, disorganised thinking, which was contrasted with the control and efficiency of stereotypically masculine thought. As such, the way in which skills were distributed between male and female brains could legitimately fit the pattern of ‘complementary stereotyping’, in which celebration of a group’s performance in low-status domains compensates for their relegation from more socially- and materially-rewarded dimensions [94].

If neuroscience research on sex differences is mobilised to purvey complementary gender stereotypes, what implications might this have for wider society? Experimental social psychological research suggests that complementary stereotypes are effective mechanisms for obscuring gender inequality and inculcating acceptance of the systems that perpetuate it [95]. This would imply that as this media content circulates through society, the complementary stereotypes embedded within it may bolster gender inequalities. The rooting of complementary stereotypes in *biology* may further intensify the system justification effect: previous experiments suggest that essentialist representations of gender categories, which portray gender differences as natural and immutable, are efficient means of satisfying system justification motives [30], [96]. Moreover, the stereotypes promulgated by the current data can avail of the epistemological authority that science holds in contemporary society, as well as the persuasive nature of neuroscientific language and imagery specifically [97], [98]. Thus, this media content has several properties that, when synthesised, may cement the social psychological processes that perpetuate gender inequality.

However, while the above experimental literature on complementary stereotypes is informative in considering the social ramifications of this month of real-world media activity, it is also important not to be overly deterministic in extrapolating from effects produced in controlled laboratory conditions. In this data, it was notable that despite strong cues from the scientific article and press release, lay commentary did not seize on biology as the exclusive determinant of gender differences. This was particularly salient in the comments data, which afforded equal attention to biological and social factors in elaborating the reported neural sex differences. Such nuances are important in highlighting that in naturalistic environments, mere exposure to reports of biological sex difference does not invariably inculcate strong belief in biological determinism. As previous research has shown, lay populations can cultivate multifactorial narratives in which biology, behaviour and socialisation mutually influence each other [99]–[101]. In addition, a small but robust strand of data directly problematised the assumptions or agendas of sex difference research, positing that it may exacerbate stereotypes and prejudice. This resonates with the emerging empirical consensus that despite the traditional media’s enthusiastic uptake of neuroscientific frames, in everyday social contexts neuroscience often elicits ambivalence, and can be rejected, remodelled or ignored [102], [103]. These critical, multidimensional properties of lay representation mean that the social psychological effects of these scientific messages are unlikely to be monolithic.

### Reflections on the study design

This analysis is unique in its comparison of material published across five sources of scientific, traditional and new media. Its concentration on a single case of science communication limits the extent of extrapolation that is possible. However, the analysis compensated in depth for what it lacked in breadth. Juxtaposing the different datasets highlighted how the unique exigencies and affordances of each communicative context were imprinted on the content it generated. The traditional media drew heavily on the press release to communicate a rather standardised account of the research to a mass audience; blogs showed a more localised accommodation of the research to the various communities with which blogs were affiliated; readers’ comments documented how individuals related scientific information about sex difference to their personal experience of gender roles and relations. Collating multiple data-sources offered a comprehensive, holistic overview of the communicative processes triggered by a new scientific report, revealing dynamics that would certainly be missed by analyses constrained to one media domain.

In particular, the inclusion of reader comments considerably enriches conventional media analysis paradigms. A perennial challenge in media analysis involves determining the extent to which media content can function as a meaningful index of public opinion, given empirical evidence that media and audience representations of scientific issues often diverge [42], [104], [105]. While comments are obviously unrepresentative of the entire range of public response, as an initial inroad into the difficult question of audience reception they offer a convenient source of data. Their naturalistic quality is a major empirical advantage, offering a rare unmediated glimpse into spontaneous social responses.

If the empirical potential of online content is to be exploited in future research, the development of reliable, consistent procedures for data collection and analysis is critical. A particularly useful resource would be a means of distinguishing the socio-demographic characteristics of the individuals or groups who produce internet content. Previous research indicates that responses to scientific ideas segment across social identities: for example, Morton et al. [106] report that people prefer scientific articles that favour their own gender, with men particularly hostile to pro-female articles; while Brescoll and LaFrance [24] find that politically conservative news outlets emphasise biological causality of sex differences proportionally more than liberally-inclined publications. In this study, informal observation of the data intimated many instances where information was selectively embraced, adapted or discredited in line with prior identity commitments. However, the heterogeneity of the data involved and the anonymous nature of much internet commentary made it impossible to reliably categorise data units according to such variables as author’s gender, culture or political orientation. Innovation in this capacity would instigate real progress in this field, facilitating a genuinely social psychological understanding of internet material.

## Conclusion

Despite some scholars’ calls for a moratorium on sex difference research [14], it seems unlikely that science or society will lose interest in searching for sex in the brain. Indeed, both the National Institutes of Health and the European Commission’s Horizon 2020 funding programmes have recently introduced policies that mandate grantees to explicitly consider the sex/gender dimensions of their research. While these decisions are guided by the commendable aim of ensuring equitable distribution of scientific advances, a socially responsible science also requires sensitivity to the social contexts in which it will be mobilised, and the social effects it may incite therein. Empirical research that tracks the sociocultural ripple-effects generated by scientific knowledge about sex difference is therefore critical. Such data would also contribute to conceptual development in social psychology, documenting how social understandings of gender interact with new knowledge, institutions and modes of communication. The nexus of science, gender and media represents a rich terrain for future research.

## Supporting Information

File S1 Traditional media articles included in the analysis.
(PDF)

File S2 Blog posts included in the analysis.
(PDF)

File S3 Comments included in the analysis.
(PDF)
