# Supplementary material for: Gender on the Brain: A Case Study of Science Communication in the New Media Environment
Source: PLoS One. 2014 Oct 29;9(10):e110830. doi: 10.1371/journal.pone.0110830 (PMC4212998; doi:10.1371/journal.pone.0110830)
Supplement: File S1 — Traditional media articles included in the analysis. (PDF) [file pone.0110830.s001.pdf]

Traditional media articles included in the analysis

ID	DATE	ARTICLE TITLE	PUBLICATION
1	30/12/13	Sex-trapolation in the Latest Brain Science	Huffington Post
2	24/12/13	Men, women's brains wired differently	Arab Times-Kuwait
3	15/12/13	Gender differences are hard-wired	News-Journal (Daytona Beach, Florida)
4	14/12/13	Brains wired differently in men and women	Business Recorder
5	14/12/13	Time to ditch the "Venus and Mars" cliche	The New Zealand Herald
6	13/12/13	Let's talk about the gender differences that matter - in mental health	The Guardian (London)
7	13/12/13	Do Men And Women Have Different Brains?	National Public Radio
8	12/12/13	Women and men are wired differently	Daily News (South Africa)
9	12/12/13	Men's and women's brains: the truth!;<br>As research proves the sexes' brains ARE wired differently, why women's are cleverer ounce for ounce - and men can't read female feelings	Irish Daily Mail
10	12/12/13	CHICKEN OR EGG? NEW RESEARCH SHOWS MEN AND WOMEN'S BRAINS ARE WIRED DIFFERENTLY	States News Service
11	12/12/13	Man and women are different -- who woulda thought?	Telegram & Gazette (Massachusetts)
12	11/12/13	Why there is no such thing as the 'male brain'	BRW
13	11/12/13	Men and women wired differently, study uncovers	Townsville Sun (Australia)
14	10/12/13	Why men (still) don't do dishes	The Globe and Mail (Canada)
15	09/12/13	WHOSE BRAIN IS BEST?	The Sun (England)
16	09/12/13	Scientists get their brain wires crossed	The Western Mail
17	08/12/13	Gender plays role in brain's wiring;<br>Mapping study suggests there are biological ties to male, female be-haviors	The Baltimore Sun
18	08/12/13	Study: The Brains Of Men And Women Are Different...Very Different	Forbes Magazine

19	08/12/13	It's time for brain science finally to ditch the 'Venus and Mars' cliche: BACK TO FRONT?: SIDE TO SIDE?: Robin McKie, who was named science writer of the year at the British Journalism awards last week, explains why he is deeply sceptical of the latest reports on basic dif-ferences in the hard wiring of male and female brains: WHAT THE NEW STUDY SHOWS	The Observer (England)
20	08/12/13	-	Sunday Herald
21	07/12/13	Boris missed the point on IQ - gifted children are failed by the system	The Guardian (London)
22	07/12/13	It's the truth, women really do multi-task better	The Independent on Saturday (South Africa)
23	07/12/13	Scientists are from Mars, journalists are from Venus	The Irish Times
24	06/12/13	Research Roundup, Dec. 6	Daily Nebraskan: University of Nebraska-Lincoln
25	06/12/13	Who's the Boss; Scientists at the University of Pennsylvannia have stirred a hornet's nest.	DNA
26	06/12/13	It's official, my brain is not my own	The Herald (Glasgow)
27	06/12/13	Toy story; THE BIG READ Why should what children play with be segregated along gender lines? CLARE DWYER HOGG speaks to the parents fighting back	The Independent (London)
28	06/12/13	Difference between men and women: brain structure	Korea Times
29	06/12/13	Sexes' grey area cleared up; The difference between male and female brains could explain why men are better at map reading and why women are better at remem-bering a conversation, writes Steve Connor	The Mercury (South Africa)
30	06/12/13	Perhaps it's time to reconsider the value of some respectful stereo-types	The Mercury (South Africa)
31	06/12/13	Leave the joined-up thinking to us, chaps	The Northern Echo
32	05/12/13	So our wiring is different - that explains a lot of things	Belfast Telegraph
33	05/12/13	What? We're different? Well I never	Irish Independent
34	05/12/13	Time of the woman	The Mercury (South Africa)
35	05/12/13	Fix the TV and take out the garbage	National Post (f/k/a The Financial Post) (Canada)
36	05/12/13	Women wired to multitask	Pretoria News (South Africa)

37	05/12/13	Juliet Dunlop: Men and women driven differently	Scotland On Sunday
38	05/12/13	It's all in the wiring	Sherbrooke Record (Quebec)
39	05/12/13	Study unveils differences in men, women's neural wiring	The Statesman (AsiaNet)
40	05/12/13	Women better than men at multitasking: Study	The Statesman (AsiaNet)
41	05/12/13	It's official: Men, women are wired differently	The Times of India (TOI)
42	04/12/13	Paper Monitor: Battle of the sexes	BBC News
43	04/12/13	IIT Delhi alumnus discovers the difference between men and women brains	Daily Bhaskar
44	04/12/13	Women wired for success..; ...but men best at throwing	Daily Mirror
45	04/12/13	IIT-Delhi alumni creates first map of neural circuitry	The Economic Times
46	04/12/13	In brief: It is pop psychology, not science, that divides the sexes	The Guardian (London)
47	04/12/13	Multi-tasking: Women have better memory, attention	Hindustan Times
48	04/12/13	The value of gender stereotypes; NOTEBOOK	i
49	04/12/13	With motor skills legendary around several DVLA test centres, I'm a stereotypical woman. And no worse for it	The Independent (London)
50	04/12/13	OOPS! You mean men and women really are different?	Jewish World Review
51	04/12/13	SARAH VINE: Our batty brains will help women takeover the world	Daily Mail (London)
52	04/12/13	Men's brains go back to front, women's go side to side	National Post (f/k/a The Financial Post) (Canada)
53	04/12/13	Why we're hemispheres apart	Northern Territory News (Australia)
54	04/12/13	Women crap at parking: Official	The Register
55	04/12/13	Brain all about sex GREY AREA	Sydney MX (Australia)
56	04/12/13	Why men can't explain women	The Times (South Africa)
57	03/12/13	Men and women's brains are wired differently: study	AFP - RELAXNEWS (English International Version)

58	03/12/13	The Difference Between Men and Women's Brains Revealed?	The Blaze
59	03/12/13	Turns out men and women really do think differently, study finds;<br>Findings suggest there are major differences in the wiring of male and female brains; researchers surprised how much the findings con-firmed stereotypes	The Globe and Mail
60	03/12/13	Proof men and women are poles apart	The Daily Telegraph (London)
61	03/12/13	SORRY CHAPS, YOUR BRAINS AREN'T MADE FOR MULTI-TASKING	DAILY MAIL (London)
62	03/12/13	Is Equal Opportunity Threatened By New Findings That Female And Male Brains Are Different?	Forbes Magazine
63	03/12/13	How men's brains are wired differently than women's	Fox News Network
64	03/12/13	Women really are better at multitasking, study of brain wiring confirms	The Guardian (London)
65	03/12/13	So, men and women's brains are wired differently - but it's not that simple	The Guardian (London)
66	03/12/13	How brains differ between genders	The Herald (Glasgow)
67	03/12/13	Turns out men and women are different	Human Events Online
68	03/12/13	Revealed at last: differences between the brains of men and women;<br>SCIENCE	i
69	03/12/13	The difference between men and women is all in the head	Irish Independent
70	03/12/13	Yes, each sex really is from different planet	Metro (UK)
71	03/12/13	Why men are better navigators, and women have better memory	MINT
72	03/12/13	Men and women wired like different species - expert	The New Zealand Herald
73	03/12/13	Brains 'different between genders', study says	News Shopper
74	03/12/13	Men and women's brains are wired differently, study finds	PBS NewsHour
75	03/12/13	Penn study: How women's brains differ from men's	The Philadelphia Inquirer
76	03/12/13	'Men, women's brains wired differently'	Press TV - Iran
77	03/12/13	Brain wired differently in men and women	Samay Live
78	03/12/13	Brains Of Men And Women 'Wired Differently'	Sky News

79	03/12/13	LET'S TALK ABOUT CORTEX, BABY..;	The Sun (England)
80	03/12/13	Brains of men and women are poles apart; Differences in the way the brains of men and women are wired helps to explain why men are better at navigating while women can multi-task	The Daily Telegraph (London)
81	03/12/13	Male and female brains are planets apart in their wiring	The Times (London)
82	03/12/13	Why Men and Women's Brains Work Differently: It's All About the Wiring	Time (USA)
83	02/12/13	The hardwired difference between male and female brains could ex-plain why men are 'better at map reading';<br>And why women are 'better at remembering a conversation'	The Independent (London)
84	02/12/13	Brain Scans May Support Venus/Mars Divide Between Sexes	Web MD
85	02/12/13	BRAIN CONNECTIVITY STUDY REVEALS STRIKING DIFFERENCES BETWEEN MEN AND WOMEN	States News Service
86	02/12/13	Why men and women behave differently - Former IIT Delhi computer science student discovers gender differences in the human brain	The Telegraph (India)
87	02/12/13	It's true, scientists say: Brains of men and women wired differently	UPI
