# Supplementary material for: Gender on the Brain: A Case Study of Science Communication in the New Media Environment
Source: PLoS One. 2014 Oct 29;9(10):e110830. doi: 10.1371/journal.pone.0110830 (PMC4212998; doi:10.1371/journal.pone.0110830)
Supplement: File S2 — Blog posts included in the analysis. (PDF) [file pone.0110830.s002.pdf]

Blog posts included in the analysis

ID	DATE	ARTICLE TITLE	BLOG TITLE	URL
1	03/12/13	Health: Contrasting Brain Wiring In Men And Women May Explain Gender Differences	CBS Philly	http://philadelphia.cbslocal.com/2013/12/03/study-different-brain-wiring-in-men-and-women-may-explain-differences/
2	03/12/13	Brain Connectivity Varies Between Men and Women	Psychology Today	http://www.psychologytoday.com/blog/the-athletes-way/201312/brain-connectivity-varies-between-men-and-women
3	11/12/13	Be Wary of Studies That Claim Men and Women's Brains Are Wired Differently	New Republic	http://www.newrepublic.com/article/115893/gender-brain-studies-claim-men-and-women-are-wired-differently
4	05/12/13	Brain scans shows male and female brains are wired differently	Wavuti	http://www.wavuti.com/4/post/2013/12/brain-scans-shows-male-and-female-brains-are-wired-differently.html
5	17/12/13	New Brain Study Illustrates Gender Differences	Cultural Detective Blog	http://blog.culturaldetective.com/2013/12/17/new-brain-study-illustrates-gender-differences/
6	02/12/13	Male and Female Brains Wired Differently	Guardian Liberty Voice	http://guardianlv.com/2013/12/male-and-female-brains-wired-differently/
7	17/12/13	Battle of the brain's sex differences…or not really?	The Brain Bank	http://thebrainbank.scienceblog.com/2013/12/17/battle-of-the-brains-sex-differences-or-not-really/
8	03/12/13	Men and women's brains are hard wired differently, study shows	ZME Science	http://www.zmescience.com/medicine/mind-and-brain/man-women-brain-03122013/
9	30/12/13	PENN STUDY: HOW WOMEN'S BRAINS DIFFER FROM MEN'S	Brain and Spine Surgeon	http://www.brainandspinesurgeon.com/2013/12/30/penn-study-how-womens-brains-differ-from-mens-philly-com-2/
10	08/12/13	Differences in the brain wiring of men and women	Free Thought Blogs	http://freethoughtblogs.com/singham/2013/12/08/differences-in-the-brain-wiring-of-men-and-women/
11	05/12/13	men are map readers and women are intuitive, but bloggers are fast	Neurocritic	http://neurocritic.blogspot.co.uk/2013/12/men-are-map-readers-and-women-are.html

12	03/12/13	Brain wiring differently between men and women	National Turk	http://www.nationalturk.com/en/brain-wiring-differently-between-men-and-women-video-strange-news-45168
13	04/12/13	Sex Differences in the Brain	Hooking Up Smart	http://www.hookingupsmart.com/2013/12/04/politics-and-feminism/sex-differences-brain/
14	12/12/13	Gender Differences in the Brain Explain Behaviour, Truth or Delusion?	United Academics	http://www.united-academics.org/magazine/mind-brain/gender-differences-in-the-brain-explain-behaviour-truth-or-delusion/
15	03/12/13	Gender differences in brain connectivity (if any) aren’t hard-wired	New APPS: Art, Politics, Philosophy, Science	http://www.newappsblog.com/2013/12/gender-differences-in-brain-connectivity-if-any-arent-hard-wired.html
16	13/12/13	TWO STUDIES ON BRAIN WIRING: THE DIFFERENCES BETWEEN THE SEXES AND PATERNAL DEPRIVATION	Business of Life	http://www.estatevaults.com/bol/archives/2013/12/13/two_studies_on.html
17	05/12/13	The sex of the brain	The Atheist Conservative	http://theatheistconservative.com/2013/12/05/the-sex-of-the-brain/
18	12/12/13	Chicken or Egg? New Research Shows Men and Women’s Brains are Wired Differently	Brain & Behavior Research Foundation	http://bbrfoundation.org/brain-matters-discoveries/chicken-or-egg-new-research-shows-men-and-women%E2%80%99s-brains-are-wired
19	10/12/13	Brain Study Confirms Gender Stereotypes	Momma Said	http://mommasaid.net/2013/12/10/brain-gender-stereotypes/
20	03/12/13	Brain imaging study shows ‘stark difference’ in neural wiring of men and women	Raw Story	http://www.rawstory.com/rs/2013/12/03/brain-imaging-shows-stark-difference-in-neural-wiring-of-men-and-women/
21	03/12/13	Male and female brains wired differently, scans reveal	Mail & Guardian Women	http://women.mg.co.za/male-and-female-brains-wired-differently-scans-reveal/
22	02/12/13	Scans May Support Venus/Mars Divide Between Sexes	Web MD	http://www.webmd.com/sex-relationships/news/20131202/brain-scans-may-support-venusmars-divide-between-sexes

23	19/12/13	Biological Differences Between Men and Women	Natural Health Blog	http://jonbarron.org/happiness-mental-health/men-and-women-really-are-different#.UvNd7_l_vYg
24	10/12/13	Brain ‘wired differently’ in men and women	u-VIB Blog	http://bloguvib.wordpress.com/2013/12/10/brain-wired-differently-in-men-and-women/
25	03/12/13	Sex on the Brain: Are Male and Female Brains Fundamentally Different?	Extravolution	http://www.extravolution.com/2013/12/sex-on-brain-are-male-and-female-brains.html
26	19/12/13	Speak to the Person, Not the Gender	Persuasive Litigator	http://www.persuasivelitigator.com/2013/12/speak-to-the-person-not-the-gender.html
27	04/12/13	Brain wired differently in men and women	abnews 24	http://abnews24.com/en/2013/12/04/brain-wired-differently-in-men-and-women/
28	05/12/13	Gender-specific findings of new brain-connectivity study are questioned	Minn Post	http://www.minnpost.com/second-opinion/2013/12/gender-specific-findings-new-brain-connectivity-study-are-questioned
29	09/12/13	Male and Female Brains Really Are Built Differently	Pandagon	http://www.pandagon.net/2013/12/male-and-female-brains-really-are-built-differently/
30	04/12/13	Male and Female Brains	An Exercise in the Fundamentals of Orthodoxy	http://www.peter-ould.net/2013/12/04/male-and-female-brains/
31	03/12/13	THE REAL REASON WHY MEN AND WOMEN ARE DIFFERENT!	Dr. Pinna	http://drpinna.com/the-real-reason-why-men-and-women-are-different-37516
32	04/12/13	Male and female brains wired differently, scans reveal	The Truth Seeker	http://www.thetruthseeker.co.uk/?p=85666
33	10/12/13	Men and Women Brain ‘Wired differently’	Today Outlook	http://www.todayoutlook.com/men-and-women-brain-wired-differently/
34	24/12/13	Sex Differences: the Male and Female Brain	Stein Center for Social Research	http://stein-center.org/2013/12/24/sex-differences-the-male-and-female-brain/
35	05/12/13	Brains of men and women wired differently	Emax Health	http://www.emaxhealth.com/11400/study-proves-men-and-womens-brains-are-wired-differently

36	03/01/14	Good read: Sex-trapolation in the Latest Brain Science	The Economy of meaning	http://theeconomyofmeaning.com/2014/01/03/good-read-sex-trapolation-in-the-latest-brain-science/
37	02/12/13	Brain Connectivity Study Reveals Striking Differences Between Men and Women	USA News	http://www.dailynewsen.com/science-news/brain-connectivity-study-reveals-striking-differences-between-men-and-women-h1351761.html
38	15/12/13	Brain wiring and science reporting	Language Log	http://languagelog.ldc.upenn.edu/nll/?p=9043
39	12/03/13	New Study Provides Earth-Shattering Insight Into Fact That Women And Men's Brains Are Different And Actually Complement Each Other	Shepherds Piehole	http://shepherdspiehole.typepad.com/shepherds-piehole/2013/12/new-study-provides-earth-shattering-insight-into-fact-that-women-and-mens-brains-are-different-and-a.html
40	06/12/13	Gender Stereotypes Explained by Hardwired Differences Between Male and Female Brains	Discovery Fit & Health Blogs	http://blogs.discovery.com/dfh-sara-novak/2013/12/gender-stereotypes-explained-by-hardwired-differences-between-male-and-female-brains.html
41	16/12/13	Destiny and the Gendered Brain	al fin next level	http://alfinnextlevel.wordpress.com/2013/12/16/destiny-and-the-gendered-brain/
42	05/12/13	Science figuring out that men and women are different	Church of God News	http://www.cogwriter.com/news/doctrine/science-figuring-out-that-men-and-women-are-different/
43	03/12/13	Men and women's brains are wired differently	Capital FM Lifestyle Magazine	http://www.capitalfm.co.ke/lifestyle/2013/12/03/men-and-womens-brains-are-wired-differently/
44	10/12/13	Gender Differences Hard Wired	The Cagle Post	http://www.cagle.com/2013/12/gender-differences-hard-wired/
45	04/12/13	Getting in a Tangle Over Men's and Women's Brain Wiring	Wired	http://www.wired.com/wiredscience/2013/12/getting-in-a-tangle-over-men-and-womens-brain-wiring/
46	04/12/13	Accepting Sex Differences Amount to "Anti-American Crazy Thinking"	Women for Women	http://womenformen.org/2013/12/04/accepting-sex-differences-amount-to-anti-american-crazy-thinking/

47	05/12/13	Bad neuroscience and gender: reading this will change your brain	NO MEASURE OF HEALTH	http://eldan.co.uk/2013/12/bad-neuroscience-and-gender-reading-this-will-change-your-brain/
48	03/12/13	Scientists Discover Difference Between Brains of Males and Females	Black Christian news	http://www.blackchristiannews.com/news/2013/12/scientists-discover-difference-between-brains-of-males-and-females.html
49	03/12/13	Scientists Prove Men & Women Do Think Differently After All	Indie Genius Productions Blog	http://indiegeniusproductions.wordpress.com/2013/12/03/scientists-prove-men-women-do-think-differently-after-all/
50	02/12/13	Brain Connectivity Study Reveals Striking Differences Between Men and Women	Scientific Outlook	http://scientificoutlook.com.np/brain-connectivity-study-reveals-striking-differences-between-men-and-women/
51	03/12/13	The Male Brain vs. the Female Brain	Had Enough Therapy?	http://stuartschneiderman.blogspot.co.uk/2013/12/the-male-brain-vs-female-brain.html
52	05/12/13	Male/Female brain "wiring" differences	Talk Rational	http://talkrational.org/showthread.php?t=60967
53	08/12/13	Study Confirms Existence of Women's Intuition	Ricochet	http://ricochet.com/main-feed/Study-Confirms-Existence-of-Women-s-Intuition
54	03/12/13	MEN CAN'T MULTITASK, WOMEN HAVE BETTER MEMORY, STUDY LED BY INDIAN-ORIGIN SCIENTIST REVEALS	Researchers Club	http://researchersclub.org/2013/12/03/men-cant-multitask-women-have-better-memory-study-led-by-indian-origin-scientist-reveals/
55	09/12/13	The Brains Of Men And Women Are Different	Abb Takk	http://abbtakk.tv/eng/the-brains-of-men-and-women-are-different-91213/
56	03/12/13	And Now for Your Gender-Biased Brain Science Update	truthdig	http://www.truthdig.com/eartotheground/item/and_now_for_your_gender-biased_brain_science_update_20131203
57	02/12/13	Women's Brains Wired Left To Right	People's Blog	http://thepeoplescube.com/peoples-blog/women-s-brains-wired-left-to-right-t12421.html
58	03/12/13	No, Our Brains Aren't Hardwired According to Gender Stereotypes	Motherboard	http://motherboard.vice.com/blog/no-our-brains-arent-hardwired-according-to-gender-stereotypes

59	11/12/13	Breaking News: Men and Women Think Differently	OAK RIDGE NOW	http://www.oakridgenow.com/2013/12/11/breaking-news-men-and-women-think-differently/
60	07/12/13	Gender differences hard-wired	Trib LIVE	http://triblive.com/opinion/tompurcell/5202455-74/women-brain-wired#axzz2sXbaE9gH
61	15/12/13	Gender Differences Hard Wired	Right Wing News	http://www.rightwingnews.com/column-2/gender-differences-hard-wired/
62	02/01/14	SHOCK STUDY REVEALS… MEN AND WOMEN ARE DIFFERENT	The College Fix	http://www.thecollegefix.com/post/15765/
63	03/12/14	The hardwired difference between male and female brains	Above Top Secret	http://www.abovetopsecret.com/forum/thread985944/pg1
64	04/12/14	Incredible Brain-Scans Reveal Men And Women's Brains ARE Wired Differently	2 Oceans Vibe News	http://www.2oceansvibe.com/2013/12/04/incredible-brain-scans-reveal-men-and-womens-brains-are-wired-differently/
65	09/12/13	Literally Messing with their Brain. What Recent Scientific Studies Can Teach Us About Ourselves and Raising our Children	Archdiocese of Washington	http://blog.adw.org/2013/12/literally-messing-with-their-brain-what-recent-scientific-studies-can-teach-us-about-ourselves-and-raising-our-children/
66	03/12/13	Confirmed: Male And Female Brains Are Wired Differently.	Tailored Chap	http://tailoredchap.com/blog/confirmed-male-and-female-brains-are-wired-differently/
67	04/12/13	Male, Female Brains Are Wired Very Differently, Scans Show	James' World	http://jamesworld119.wordpress.com/2013/12/04/male-female-brains-are-wired-very-differently-scans-show/
68	04/12/13	PNAS: male, female brains wired along different paths	the reference frame	http://motls.blogspot.co.uk/2013/12/pnas-male-female-brains-wired-along.html
69	12/12/13	A Silly Sex Post	A View from the Beach	http://fritz-aviewfromthebeach.blogspot.co.uk/2013/12/a-silly-sex-post.html
70	04/12/13	Different Brain Wiring Is What Makes Men and Women Different	ABC News Radio Online	http://abcnewsradioonline.com/health-news/different-brain-wiring-is-what-makes-men-and-women-different.html

71	05/12/13	Men and women wired differently	longevity LIVE	http://www.longevitylive.com/a_health/men-and-women-wired-differently/
72	03/12/13	Women are hardwired to think without thinking	Godlike Productions	http://www.godlikeproductions.com/forum1/message2426619/pg1
73	04/12/13	Scientists discover difference between male and female brains	Lahore Times	http://www.lhrtimes.com/2013/12/04/scientists-discover-difference-between-male-and-female-brains-213880/
74	03/12/13	Sorry lads, girls really are better at multi-tasking: Proof brains really are different	Daily Star	http://www.dailystar.co.uk/news/latest-news/353900/Sorry-lads-girls-really-are-better-at-multi-tasking-Proof-brains-really-are-different
75	05/12/13	New Study: The hardwired ‘stark’ difference between male and female brains explains why women are more intuitive — “It’s quite striking how COMPLEMENTARY the brains of women and men really are.” | God knew what He was doing :)	ONE can happen	http://onecanhappen.wordpress.com/2013/12/05/study-the-hardwired-stark-difference-between-male-and-female-brains-explains-why-women-are-more-intuitive-its-quite-striking-how-complementary-the-brains-of-women-and-men-rea/
76	04/12/13	Men vs. Women, Nature vs. Nurture	Jesus Creed	http://www.patheos.com/blogs/jesuscreed/2013/12/04/men-vs-women-nature-vs-nurture/
77	03/12/13	Study: Men Only Using Half a Brain	Pat’s Papers	http://www.patspapers.com/story_stack/item/study_men_only_using_half_a_brain/
78	03/12/13	A quick moan about ‘male’ and ‘female’ brains	The Tangled Woof of Fact	http://tangledwoof.wordpress.com/2013/12/03/a-quick-moan-about-male-and-female-brains/
79	08/12/13	MALE & FEMALE BRAINS ARE WIRED DIFFERENTLY	Super Human	http://superhuman.ly/2013/12/08/male-female-brains-are-wired-differently/
80	03/12/13	Men and women’s brains are ‘wired differently’	In2EastAfrica	http://in2eastafrica.net/men-and-womens-brains-are-wired-differently/
81	03/12/13	WASHINGTON: Men can’t multitask and women have better	Pakistan Defence	http://defence.pk/threads/men-cant-multitask-women-have-better-memory.289759/

		memory because their brains are wired differently, a new study led by an Indian-origin scientist has found.		
82	03/12/13	Men’s and women’s brains are different	Neo-Neocon	http://neoneocon.com/2013/12/03/mens-and-womens-brains-are-different/
83	03/12/13	New DUH Moment From The World Of Academic Medical Research	The Conservative Citizen	http://constitutionclub.org/2013/12/03/new-duh-moment-from-the-world-of-academic-medical-research/
84	09/12/13	Brains of men and women are wired differently	Professional Energy Healers	http://www.professionalenergyhealers.com/brains-of-men-and-women-are-wired-differently/
85	07/12/13	Sex and brains: Vive la différence!	Gonzalo Raffo InfoNews	http://gonzaloraffoinfonews.blogspot.co.uk/2013/12/sex-and-brains-vive-la-difference.html
86	30/12/13	Great Graphic: Neural Networks in Men and Women	Marc to Market	http://www.marctomarket.com/2013/12/great-graphic-neural-networks-in-men.html
87	11/12/13	Science reporters fail to dig deep enough on male v. female brain story	Genetic Literacy Project	http://www.geneticliteracyproject.org/2013/12/11/science-reporters-fail-to-dig-deep-enough-on-male-v-female-brain-story/#.UvN_Wfl_vYg
88	03/12/13	Biden: ’I’ve Never Found’ Women in Workforce to Be ’Kinder and Gentler’	Lem’s Levity	http://comonocreerendios-lem.blogspot.co.uk/2013/12/biden-ive-never-found-women-in.html
89	03/12/13	Brains Of Men And Women ‘Wired Differently’	Kwachanji	http://www.kwachanji.com/brains-of-men-and-women-wired-differently/
90	13/12/13	Venus + Mars Strike Again: Does Gender Affect Great Customer Service Agents?	Business 2 Community	http://www.business2community.com/customer-experience/venus-mars-strike-gender-affect-great-customer-service-agents-0706411
91	28/12/13	This Week in the War on Women: December 28	Daily Kos	http://www.dailykos.com/story/2013/12/28/1265738/-This-Week-in-the-War-on-Women-December-28#
92	03/12/13	Male And Female Brains Are Planets Apart In Their Wiring	Working with God	http://workingwithgod.com/?p=289

93	12/13	Why Women Have Sharper Memory than Men	Raha Tupu Wahubwa Info Site	http://www.rahatupu.info/2013/12/why-women-have-sharper-memory-than-men.html
94	04/12/13	in brief: women predisposed to multitasking, finds study	lip THINK ABOUT IT	http://lipmag.com/news/in-brief-women-hard-wired-for-multitasking-finds-study/
95	04/12/13	Brains of Women and Men Show Strong Hard-wired Differences	Dementia Today	http://www.dementiatoday.com/brains-women-men-show-strong-hard-wired-differences/
96	03/12/13	Male and female brain connections are completely different	BELLE NEWS	http://www.bellenews.com/2013/12/03/health/male-and-female-brain-connections-are-completely-different/
97	03/12/13	Study Proves Male and Female Brains Are Wired Differently	FOX NEWS [INSIDER]	http://foxnewsinsider.com/2013/12/04/study-proves-male-and-female-brains-are-wired-differently
98	03/12/13	Male and Female “Wired” Differently According to Study	Science Learning Centres	http://www.sciencelearningcentres.org/science/male-and-female-wired-differently-according-to-study/
99	03/12/13	Men and Women Are Wired Differently, Listen to Katy Perry and John Mayer’s New Song and More!	SELF	http://www.self.com/blogs/flash/2013/12/lunch-break-how-male-and-female-brains-are-wired-differently-john-mayer-katy-perry.html
100	03/12/13	Male and Female Brains Wired Differently, Scans Reveal	Conscious Life News	http://consciouslifenews.com/male-female-brains-wired-differently-scans-reveal/#
101	26/12/13	Differences In Male/Female Brains: 3 Reasons For Fledgling Relationships	His Leadership Her Trust	http://hisleadershiphertrust.com/differences-in-malefemale-brains-3-reasons-for-fledgling-relationships/
102	03/12/13	Men and women’s brains are ‘wired differently’	Life Daily	http://lifedaily.net/men-and-womens-brains-are-wired-differently/
103	06/12/13	WOMEN BETTER LISTENERS THAN MEN	DAWNPAGES	http://dawnpages.wordpress.com/2013/12/06/women-better-listeners-than-men/
104	03/12/13	Brain scans and ‘good’ mothers…	Cedar Lounge	http://cedarlounge.wordpress.com/2013/12/03/brain-scans-and-good-mothers/

105	03/12/13	Study: Men’s And Women’s Brains Wired Differently	CBS New York	http://newyork.cbslocal.com/2013/12/03/study-mens-and-womens-brains-wired-differently/
106	03/12/13	The hardwired difference between male and female brains	I Hate Men	http://www.ihatemen.org/2013/12/03/the-hardwired-difference-between-male-and-female-brains/
107	21/12/13	Beyond Lazy Language: Hopes For Future Science Discourse	Bluestockings Magazine	http://bluestockingsmag.com/2013/12/21/beyond-lazy-language-hopes-for-future-science-discourse/
108	12/13	The brains of men and women are connected differently	World of Health	http://world-of-health.com/the-brains-of-men-and-women-are-connected-differently/
109	04/12/13	What’s For Breakfast? Fried Girl and Boy Brainz! How Men’s And Women’s Brains are Dramatically Different And What It All Means.	Echidne of the Snakes	http://echidneofthesnakes.blogspot.co.uk/2013/12/whats-for-breakfast-fried-girl-and-boy.html
110	12/12/13	Men and Women’s Brains Are ‘Wired Differently’	The Aquila Report	http://theaquilareport.com/men-and-womens-brains-are-wired-differently/
111	09/12/13	Asperger definition is like male brain	The Angry Dad	http://blog.angry-dad.com/2013/12/asperger-definition-is-like-male-brain.html
112	03/12/13	Men and women’s brains are ’wired differently’	MGTOW FORUMS	http://www.mgtowforums.com/forums/mgtow-general-discussion/20814-men-womens-brains-wired-differently.html
113	03/12/13	Study reveals men and women’s brains are wired differently	The Malaysian Times	http://www.themalaysiantimes.com.my/study-reveals-men-and-womens-brains-are-wired-differently/
114	03/12/13	The Hardwired Difference Between Male and Female Brains	iTulip	http://www.itulip.com/forums/showthread.php/26380-The-Hardwired-Difference-Between-Male-and-Female-Brains
115	19/12/13	Does size matter? Pink brains blue brains.	Bite Sized Brains	http://bitesizedbrains.com/2013/12/19/does-size-matter-pink-brains-blue-brains/
116	04/12/13	Connectivity: The Difference Between Men’s and Women’s Brains — PsyBlog	Yatin J. Patel	http://yatinjpatel.com/2013/12/04/connectivity-the-difference-between-mens-and-womens-brains-psyblog/

117	15/12/13	Differences in How Men and Women Think Are Hard-Wired; Recent Studies Raise the Possibility That Male Brains Are Wired for Focus, Female Brains for Multitasking	Bamboo Innovator	http://bambooinnovator.com/2013/12/15/differences-in-how-men-and-women-think-are-hard-wired-recent-studies-raise-the-possibility-that-male-brains-are-wired-for-focus-female-brains-for-multitasking/
118	06/12/13	The sex of a brain	The Connectivist	http://www.theconnectivist.com/2013/12/throughthewire-126/
119	03/12/13	New brain study suggests men and women are wired differently.	I Hate the Media	http://www.ihatethemedia.com/new-brain-study-suggests-men-and-women-are-wired-differently
120	03/12/13	Another Study Proves Nature is Sexist	Margery + The Man	http://margeryandtheman.wordpress.com/2013/12/03/another-study-proves-nature-is-sexist/
121	03/12/13	Men, Women, and Big PNAS Papers	Neuroskeptic	http://blogs.discovermagazine.com/neuroskeptic/2013/12/03/men-women-big-pnas-papers/#.UvOhBfl_vYg
122	12/13	We Really Are Wired Differently!	Malcolm OUT LOUD	http://www.malcolmoutloud.tv/tools/?p=4552
123	11/12/13	Science supports complementarianism! Warning: political incorrectness within– enter at your own risk!	Bereans @ The Gate	http://bereansatthegate.com/2013/12/11/science-supports-complementarianism-warning-political-incorrectness-within-enter-at-your-own-risk/
124	03/12/13	Men and women's brains are different- it's official	Health Direct	http://www.healthdirect.co.uk/2013/12/men-and-womens-brains-are-different-its-official.html
125	13/12/13	Calling All Female Brains: Stop the 'Neurosexism'	Women's eNews	http://womensenews.org/story/women-in-science/131212/calling-all-female-brains-stop-the-neurosexism#.UvOq9fl_vYg
126	03/12/13	Men and women's brains are wired differently	mySarawak	http://www.mysarawak.org/2013/12/men-and-womens-brains-are-wired-differently.html
127	03/12/13	Men and women's brains are 'wired differently'	Naija247 News	http://naija247news.com/men-womens-brains-wired-differently/#.UvOq_vl_vYg
128	11/12/13	Cordelia Fine - New Insights into Gendered Brain Wiring, or a Perfect	The Masculine Heart	http://masculineheart.blogspot.co.uk/2013/12/cordelia-fine-new-insights-into.html

		Case Study in Neurosexism?		
129	11/12/13	Gender and brains again? (Ugh)	ronnerio	http://ronnerio.com/2013/12/11/gender-and-brains-again-ugh/
130	09/12/13	Male and Female Brains… Again: Why Scientists Should Read Feminist Theory by Ángela Lavilla Cañedo	School of Languages and Cultures Postgrads	http://sheffieldslcblog.wordpress.com/2013/12/09/male-and-female-brains-again-why-scientists-should-read-feminist-theory-by-angela-lavilla-canedo/
131	04/12/13	Men and Women's Brains Are Wired Differently: Study	Medical Observer	http://medicalobserverph.com/men-and-womens-brains-are-wired-differently-study/
132	03/12/13	Men Are from Mars, Women Are from Venus-striking differences in the neural wiring of men and women	Interesting News From All Over	http://interestingnewsfromallover.blogspot.co.uk/2013/12/new-evidencemen-are-from-mars-women-are.html
133	26/12/13	Does Islam Suggest Women are Inferior in Worth and Intellect?	Citizen of Islam	http://www.citizenofislam.com/2013/12/does-islam-suggest-women-are-inferior.html
134	12/13	Men, women's brains wired differently	IBN TV	http://www.ibn-tv.com/2013/12/men-womens-brains-wired-differently/
135	12/12/13	MALE BRAIN VERSUS FEMALE BRAIN (AND VICE VERSA)	Dome of Glass	http://domeofglass.com/2013/12/12/male-brain-versus-female-brain-and-vice-versa.aspx
136	03/12/13	This just in... men and women are different	Brutally Honest	http://www.brutallyhonest.org/brutally_honest/2013/12/this-just-in-men-and-women-are-different.html
137	05/12/13	Women brains and men brains	Identifeye	http://identifeye.wordpress.com/2013/12/05/women-brains-and-men-brains/
138	28/12/13	Why it matters that men and women are different	The Matt Walsh Blog	http://themattwalshblog.com/2013/12/28/why-it-matters-that-men-and-women-are-different/
139	10/12/13	The Many Other Wonderful Words For "Brain"	Sparklife	http://community.sparknotes.com/2013/12/10/the-many-other-wonderful-words-for-brain
140	09/12/13	We Shall Overcome	deshocks	http://deshocks.com/2013/12/09/we-shall-overcome/
141	10/12/13	Brain Connectivity Study Reveals Striking	AANS Neurosurgeon	http://www.aansneurosurgeon.org/2013/12/10/brain-connectivity-study-reveals-striking-differences-between-the-sexes/

		Differences Between the Sexes		
142	31/12/13	Capitalism, Ecology And The Official Invisibility Of Women	Popular Resistance	http://www.popularresistance.org/capitalism-ecology-and-the-official-invisibility-of-women/
143	04/12/13	SHOCK! HORROR! MEN AND WOMEN ARE DIFFERENT!	Father Ed Tomlinson's Blog	http://www.tunbridgewells-ordinariate.com/blog/?p=9581
144	12/13	Sex and brains: Vive la différence!	Life science log	http://www.lifesciencelog.com/cluster2017399500/
145	11/12/13	Recent Studies Raise the Possibility That Male Brains Are Wired for Focus, Female Brains for Multitasking	San Diego Education Report Blog	http://learningboosters.blogspot.co.uk/2013/12/recent-studies-raise-possibility-that.html
146	02/12/13	Female brain is 'hard-wired' to be better at multi-tasking.	collection	http://bbhazghy.blogspot.co.uk/2013/12/female-brain-is-hard-wired-to-be-better.html
147	08/12/13	"As hardy perennials go, there is little to beat that science hacks' favourite: the hard-wiring of male and female brains."	Althouse	http://althouse.blogspot.co.uk/2013/12/as-hardy-perennials-go-there-is-little.html
148	26/12/13	Girls' brain connections may explain their faster maturity	Human Rights Observers	https://hromedia.com/2013/12/26/girls-brain-connections-may-explain-their-faster-maturity/
149	04/12/13	Brains of Males and Females Are Wired Differently	Web Pro News	http://www.webpronews.com/brains-of-males-and-females-are-wired-differently-2013-12
150	12/12/13	Brains of women, men are actually wired differently	The Columbian	http://www.columbian.com/news/2013/dec/12/brains-of-women-men-are-actually-wired-differently/
151	05/12/13	Should women run the world?	Bill Longstaff	http://blongstaff.blogspot.co.uk/2013/12/should-women-run-world.html
152	04/12/13	'Men and women's brains are wired differently'	The Morning News	http://themorningnewsaruba.com/health/men-and-womens-brains-are-wired-differently/

153	03/12/13	Male And Female Brains Are Wired Differently, Alert The Feminists	Chateau Heartiste	http://heartiste.wordpress.com/2013/12/03/male-and-female-brains-are-wired-differently-alert-the-feminists/
154	03/12/13	The Science Is Settled: Feminism Is Wrong	The Other McCain	http://theothermccain.com/2013/12/03/the-science-is-settled-feminism-is-wrong/
155	04/12/13	People Are Still Saying This??	Feminist Philosophers	http://feministphilosophers.wordpress.com/2013/12/04/people-are-still-saying-this/
156	12/04/13	Diffusion tensor imaging: girls, boys brains are different at all development stages	California’s Children	http://californiaschildren.typepad.com/californias-children/2013/12/diffusion-tensor-imaging-girls-boys-brains-are-different-at-all-development-stages.html#.UvO56fl_vYg
157	04/12/13	Are men’s and women’s brains truly wired differently?	NEWSFIX	http://newsfixnow.com/2013/12/04/are-mens-and-womens-brains-truly-wired-differently/
158	06/12/13	BRAIN ANALYSIS STUDY FINDS SIGNIFICANT DIFFERENCES BETWEEN THE GENDERS	rb.trends	http://www.richardbanks.com/trends/2013/12/06/brain-analysis-study-finds-significant-differences-between-the-genders-brains/
159	03/12/13	A neurological explanation for gender stereotypes	current, medicine, ethics and my experiences	http://medblog.medlink-uk.net/haider302/2013/12/03/a-neurological-explanation-for-gender-stereotypes/
160	03/12/13	The Real Difference Between The Male And Female Brain	FOOYOH	http://fooyoh.com/iamchiq_living_lifestyle/9370035/the-real-difference-between-the-male-and-female-brain
161	03/12/13	New study just out - men’s brains and women’s brains really are wired up differently! Good or bad?	Lucy Melford	http://lucymelford.blogspot.co.uk/2013/12/new-study-just-out-mens-brains-and.html
162	23/12/13	Your at-a-glance guide to psychology in 2013 - Part 2	BPS Research Digest	http://bps-research-digest.blogspot.co.uk/2013/12/your-at-glance-guide-to-psychology-in_23.html
