# Supplementary material for: Gender on the Brain: A Case Study of Science Communication in the New Media Environment
Source: PLoS One. 2014 Oct 29;9(10):e110830. doi: 10.1371/journal.pone.0110830 (PMC4212998; doi:10.1371/journal.pone.0110830)
Supplement: File S3 — Comments included in the analysis. (PDF) [file pone.0110830.s003.pdf]

Comments included in the analysis

This document contains all comments that were included in the analysis. The usernames of the individuals who contributed the comments have been removed as some contain identifying information. Comments are grouped according to the online article under which they appeared. Within the main manuscript, quoted comments are identified first in terms of the source article and then by their order within the set of comments selected from that article. For example, 'comment 15:2' indicates the second comment extracted from article 15 ('Whose brain is best?', The Sun, 09/12/13).

1	30/12/13	Sex-trapolation in the Latest Brain Science	Huffington Post

1
"It begins in the PNAS paper itself, whose authors depict male-female differences in the most extreme way -- a wiring diagram showing only the differences, but hiding the considerable overlap in the data that other studies do depict."

The overlap isn't interesting, though. We're the same species, do all the same basic functions, so of course most of it will be the same. Sex differences are explained by differences between the sexes, not the similarities between the sexes.

"Next comes the World Wide spin cycle, where an international cadre of science journalists somehow decides that these adolescent differences are "hardwired," meaning, fixed from birth. Remember, the differences were not even observed in younger children. […] What about all those countless hours boys spend playing action-packed video games while girls are busy gossiping on Facebook?"

1) Hardwired means biologically encoded, not fixed from birth (conception, really). Are developed breasts not hardwired just because they don't appear until later in development?
2) You suggest that the observed differences are the result of sexually dimorphic activities, yet what causes that divergence? It's not simply cultural exposure. And even if it was, where, evolutionarily, did the cultural sexual dimorphism come from, except from differences in biology?

6	13/12/13	**Let's talk about the gender differences that matter - in mental health**	**The Guardian (London)**

1
"So, given the scale of the additional distress these figures imply for women, why aren't we talking about it?"
Surely the inequality of Women in society is talked about a great deal, at great length on every available media stream, including mental health. Nothing wrong with that of course, but the idea that its a niche subject or somehow overlooked over is laughable. What's not so laughable is that men's problems are typically (and in the article also) downplayed. Men commit suicide in greater numbers he observes, but this is not such a problem compared with the numbers of women **attempting**suicide, as if this is a zero sum game. As someone with a son with mental health issues I find that the emphasis is predominantly on female and children's health when it coms to discussion in the media.

2
A lot of people (the majority are women) who are saddled with that label Borderline Personality Disorder are living with the legacy of abuse. It's a lazy "diagnosis"; as long as doctors slap a label like that on someone, they don't have to face the ugly fact that abuse happens, usually in families. Much easier to suggest that there is an inate fault within an individual person.
Your claim about women with this diagnosis being supported by the courts is debatable, to say the least. I am not an expert but women I have known who *were* severely disordered (for whatever reason) lost custody of their children. Because it could be demonstrated to the courts that their ongoing chaos, their repeated admissions to hospital, their repeated self-mutilation, etc etc were not conducive to decent parenting.
Someone very ill with bipolar (esp if not benefitting from medication) is also likely to be a poor parent and to "wreak havoc", gender notwithstanding. Ditto with someone who has PTSD, gender notwithstanding.

3
If you define criminal conduct as a mental disorder (and why not?), then men are about ten times more.
Autism is also four times more common in males, and milder autism ten times more common in males, but then that doesn't fit the usual guardian gender-agenda!
Reason for autism imbalance is explained in the paper at third link at the top of this page here:
www.autismcauses.info

4
It is my opinion that equality in the workplace is still some way off.
It is also my opinion that paying their way in restaurants and bars is also still some way of

7	13/12/13	Do Men And Women Have Different Brains?	National Public Radio

1
or both...
who was it that said "nature is nurture"?

2
no, silly bugger...
i’m thinking jane goodall... or maybe helen fisher? i just can’t recall...

3
Personally I believe it’s environmental and thus psychological. Social gender differences have an effect on, well not necessarily brain development, but our thought processes and how we perceive ourselves and others in the world around us.
I think a woman’s ability to create and nurture a human life frightens both men AND women. It’s a pretty serious responsiblity, a blessing and a curse really.

4
Consider a tree. (I prefer Maple)
It is coded to grow in a certain way - straight, tall trunk, leaves, branches, pancake toppings, etc.
Environmental conditions can affect its ability to develop, or you can even force it to grow in unusual shapes, but as soon as you stop it will return to its natural patterns.
Or even better, think of how severe a trauma must be to prevent a childs arm from growing.

5
I see those arguing that neural differences don’t a make a difference as thinking with their genitals. Gender as purely a social construct is a social construct. Feminist and LGBT activist academics need to get over It and maybe apologize to all those who have been harmed by the continued promotion of that myth. They have used and abused both the intersexed and transsexed for long enough.

9	12/12/13	Men's and women's brains: the truth!; As research proves the sexes' brains ARE wired differently, why women's are cleverer ounce for ounce - and men can't read female feelings	Irish Daily Mail

1
This explains why my boyfriends never get it but when I tell my girlfriends they agree with me 100%.

2
I think it has a lot more to do with upbringing and the pushing of gender roles on children from an early age. If it were caused by something as rigid and factual as brain structure, why would there be so many exceptions to these rules?

3
Women think too much. Saying "It’s sunny today" makes your woman wonder if you’re really thinking "I wonder if there are sexy women in mini skirts out today?". Years ago, I started a period of work as part of my degree, my girlfriend started seeing someone else. Dumped! What about PMS, when a woman can become a complete and utter 2@?

4
What a pile of crap! Grown up women don’t get "overemotional", they’re used to stress and don’t waste time on "emotions". I, for one, am an excellent map reader and can compete for 1st price in parallel parking competition. Although it is true that women are more intuitive, memories for words and faces cannot be generalized. Bottom line, this article wants to place people into cookie-cutters and it does not work. I coincide with another reader herein that wonders just how much was spent in this idiotic study...

5
" men can’t read female feelings" - Not when the TV is on.

6
As an American woman, living in London I have to say, there is no surprise here. We have known it all along, haven’t we girls? I am a successful lawyer, and am often told by my boss that I have the best legal mind he has even known. Nearly all the other partners are men and I have a much greater success rate and am in much more demand than them. - janeyBB, London-Boston, United Kingdom, 05/12/2013 10:20 Your boss knows compliments work. He probably wants to sleep with you....

7
’emotional intelligence’ I’m not feeling it.

8
That is the whole idea, men and women were supposed to complement each other. And together, create an union. I know, I am married and experience this on a daily basis. Read the book ’men are from Mars and women are from Venus’. Helps you understand why and how to accept the differences.

9
"WHY WOMEN LOVE FUNNY MEN" because they are so rare, like diamonds.

10
This is why it’s takes three men, and three meetings to start what one women can accomplish on her own. That said, men and woman who work together and leave their egos at home, can accomplish wonders. Blue, Manchester.

11
But the media and PC lefties would have us believe we are exactly the same, because gender is something made up to oppress women

12
Winning a losing battle? Oh how you wish. As for pathetic and sexist comments you are a master at them. Nothing interesting about you boring and whinging feminists......... MUST truly suck to be that.

13
Women are just plain weird. That’s a scientific fact.

14
My bus driving pal experiences the more intelligent females everyday at his job, driving their car right up to the bus when cars are parked on their side in a narrow street and expecting him to reverse the bus back. Everyday he says.

15
Blegh. Hate all this male female rubbish. Just pushes the sexes further apart then they have to be. People are so funny believing all they read in pop psychology. Even real psychological studies have their flaws. Again though, lets just believe everything we read in the media and let it define the way they think!

16
It might make more sense to spend some time testing each gender with control groups according to "handed-ness" viz. ambidextrous, left- and right-handed. I’m an ambidexter and can multitask etc competently. I had no trouble hearing our children cry, and I can can read maps , charts and schematics extremely well. My wife can read maps, reverse park well enough to put most men to shame. I suspect there’s a fair degree of overlap in any event.

17
If this articles heading was the reverse, it would be seen as sex discrimination. Oh by the way, I’m writing on an instrument invented by a man and you are looking at something through an instrument made by a man. And when you brushed your teeth in the morning, you used an instrument invented by a man. If you drive? And your father is a ?

18
Apart from Mde. Curie when are the women going to Invent / Discover anything worthwhile????????

19
Actually, no. To everything in this article. What the imaging really tells us is that a statistically significant proportion of men tends to show activity confined within a hemisphere when performing certain complex tasks and a statiscally significant proportion of women tends to show activation spanning both hemispheres when performing the same tasks. What these tasks were was not explained (rushing to aid a crying baby or staring into somebody’s eyes, let alone repairing a car, while inside a MRI unit, was probably not among them) but I would hazard a guess and say they involved simple maths, reading a map, reading some sentences and maybe looking at some pictures. Does this really tell us that men are useless at looking after their children? I find that extremely insulting to those millions of single dads that do extremely good job and are just as good as ’reading emotions’. Every neuroscientist knows the differences between indivduals are bigger than between any given group.

20
Yeah, yeah, yeah...another "man bad/woman good" story. It’s all baloney. If women were truly better wired, mentally speaking, or "efficient", then how come all the great inventions over the past 2000 years come from the brains of men? How come almost all of the great medical breakthroughs have come from men? How come all of the knowledge gained from the cosmos to this point has come to us through men? Other than a Madame Curie here and there most everything known in existence to modern humanity came about through the brains of men, so quit publishing these moronic bs stories where women are supposedly superior or more special ’cause quite simply we ain’t buyin’ it. We never have and we never will.

21
My wife has insists I’m emotionally unstable for getting so angry when I get hurt working around the house. So, I tone it down when she’s home. One day, while she was at work, I was home working on

our car. A wrench slipped and skinned my knuckles and it hurt. I let loose an angry, profanity laced rant only to realize at the end, a neighbor kid had been standing behind me the whole time. We looked at each other for a long moment and the kid said, "You’re crazy." and walked away. I’ve never told my wife and doubt I ever will.

22
Wow this explains a lot lot lot!!!!

23
Men are more logical...who do you want making decisions?

24
It’s because only women have ’alien’ DNA. These new studies prove what we men always suspected that they’re clones of their mothers.....

15	09/12/13	WHOSE BRAIN IS BEST?	The Sun (England)

1
Of course husbands of high-powered moms working on Wall St don't cook & don't clean house. Why on earth would they? They have people to take care of those tasks because they can afford it.

Wives of high-powered dads working on Wall St aren't exactly on their knees scrubbing toilets.

2
This is bunk. We split the doggycare and my husband does ALL the cleaning. He is still a manly man. He is much better at dishes and vacuuming than I am. I cover all the things he hates to do but that make our lives better - cooking, gardening, sending cards and gifts, planning parties, budgeting, and calling ailing family members. It works well for us.

3
My husband and I have no "housework gap," but then again, we don't share Ms. Wente's Stepford wiring. Ours is more knob-and-tube: we're gay.

4
"My husband likes to leave his cereal bowl on the kitchen counter, where it can develop a nice, hard crust..."
That has nothing to do with him being a man and everything to do with him being a lazy slob.

5
Oddly enough my wife doesn't seem to recognize dust bunnies and is not great on neat and tidy. I've been doing the dishes since I was old enough to reach the sink. Madame Went has never outgrown the 1950s.

6
As a Father or 3 and a House Spouse I do everything, but it is not as difficult as it is traditionally portrayed.

7
Funny thing......I JUST finished what appears to be every cook pot and piece of silverware in the house......(thanks to my child bride spending all morning cooking)............................

8
First of all, I do all of the dishes in our house and at my son's and daughter's house when we visit and have a meal. I actually enjoy doing dishes, I find it relaxing (while I'm sipping a glass of my favorite wine).

9
my wife is happy that i cook and clean up after.
she does everything else.

10
You MEN, the OPPRESSORS!!!!!!!

It is now time for the sisterhood to put these white males in their proper place.

Please join us for some consciousness raising at the corner of Bathurst and Bloor. Guerrilla theatre will be performed by the womYn will bring their djembes so that we can rise up against the scourge of white male oppression and privilege.

11
The study Ms Wente refers to did not claim people were 'hardwired' for anything. It observed differences in male and female neural pathways and didn't pass comment on whether these are

predetermined or not. Neural plasticity means they could be formed by behaviour reinforced by cultural norms as well as genes.

12
Be careful trying to extract your experience as a baby boomer to the next generation (who are now late 20s to 30s!)
Many many many men do dishes, cook, clean the bathroom and dust. Many many many women do none of these . Lazy people exist in both sexes. But your generation doesn’t do the same as the next any more than your generation is the same as your parents.

**18**	**08/12/13**	**Study: The Brains Of Men And Women Are Different...Very Different**	**Forbes Magazine**

1
I would like to see a longitudinal study up to great old age, myself; if puberty changes brain wiring expression, wouldn’t a shift in hormones in later years do the same? (Ambitious grad students–surely there’s a doctorate in there for someone with a study like that).

**19**	**08/12/13**	**It's time for brain science finally to ditch the 'Venus and Mars' cliche: BACK TO FRONT?: SIDE TO SIDE?: Robin McKie, who was named science writer of the year at the British Journalism awards last week, explains why he is deeply sceptical of the latest reports on basic differences in the hard wiring of male and female brains: WHAT THE NEW STUDY SHOWS**	**The Observer (England)**

1
Has any work been done on pre and post pubescent transgendered adolescents? To see how much *(if any) neurolgical differences, to see how much of a factor sex hormones have in brain development.

2
"Could", except there’s not much reason to believe it.
There have been biological boys raised as girls, like as David Reimer. Things didn’t turn out well for them. His peers had no idea he was a boy, yet he found himself playing with trucks over Barbie dolls. He eventually committed suicide following a severe depression.

3
Or compare the brains of male and female artists, chess players, mathematicians, chefs, teachers, social workers, writers, engineers...
I can be creative and logical. So where do people like me fit in with the stereotypes? As was said in the article, the interesting thing is why people seem to want to reinforce their prejudices.

4
"There is little evidence to suggest differences between male and female brains are caused by anything other than cultural factors.." to voice my educated opinion: the big difference is due to hormonal levels, phases, and cycles

5
Why bother having your sex in your head when you can have a brain controlling your sex?
Gender trumps sex in any case: mind over matter (apart from those who can’t control themselves, of course) and deconstructing gender stereotypes allows us to understand that gender is no more than a political instrument to control the behaviour of those who are convinced by the need of it.

6
I am curious: If male and female brains are the same at birth, and then only shaped only by environment, how do you explain transsexuals? Here we have people who have been brought up and seen as one sex by society and parents, yet they have developed the brain of the opposite sex.
All environmental factors point to them developing personalities connected to the sex they are seen to have yet the brain refuses. Do you claim that all trans sexual are delusional? Or maybe the environment they have been brought up hasn’t been sexist enough? Well then why would they want to change sex? Wouldn’t they not care about their physical sex?
I’m sorry, but transsexuals, are to control group that disproves your theory.
The argument that we are all the same is futile, and loses sight of the real point: that we treat others well disregarding how they think and what they look like. Respect for others should not be dependent on other reaching some bar of being enough like us, it should be implicit for everyone.

7
While the PNAS authors’ conclusions might lack direct causative evidence, it’s hardly a huge leap to propose that these differences in brain-wiring are natural rather than the result of nurture.
We accept that males are far more likely to get autism and females are much more likely to suffer from anxiety: is this all nurture too? What about big muscles or breasts, did society foster these for us too? There are sound biological and evolutionary reasons to suppose sexual differentiation for psychological traits, why do some media outlets find this so hard to swallow?

I think I know the answer: some people think that everyone has to be created psychologically identical otherwise it’s sexist. Not so: just because males and females have different psychological tendencies doesn’t make anyone better.
We can acknowledge trends without having to say, ’all women are X, all men are Y’.
We can be equal without having to be identical.

8
Basing only on gender to conclude the difference of brain wiring is over simplistic.
Sure, there will be difference based on gender. However, the study does not seem to take into account the professions of the samples.
How about the brain of women who work in male dominant areas or activities?
How about men who work in women dominant areas or activities?
I suspect this also affect a lot the way one’s brain is wired.

9
I disagree. Of course there are innate differences in male and female brains. We have fulfilled different roles for millenia. The evolved brain is bound to reflect that.

10
What’s really weird is these neo-maoist brains that are so bent on the ideology of equality that they really must believe that there is no substantial difference between men and women, whatever the evidence.
Anyone with a bean of good sense is not going to dismiss human anthropolgy and evolution to satisfy a few fanatics and their ivory tower wishful thinking.
And there is a lot given up with it: the complementarity of the sexes should be a joy to each other.
Vive la difference!

11
We don’t ’let’ men think they’re in charge. They think it whether we want them to or not - or at least some of them do.
Plus, all these men saying ’we love the way you women are different to us’ makes me suspect that they get annoyed about the ways we are similar to them. I sometimes suspect misogyny (which I’m NOT accusing you of) is just men’s projected self hatred towards women (’bad’ women) that remind men of themselves.

12
She also sets herself up as this heroic defender of the abilities of women by creating an entirely false perception of what conclusions have been reached by "male chauvinist pig" scientists She does this by projecting false words into their mouths and ignoring the general consensus within the scientific community about the interaction betweeen nature and nurture. She tries to lend support to one extreme position by creating this false demon at the opposite extreme, and attacking, while providing no basis of support for her own extreme position. It’s exactly like arguing "If Chewbacca lives on Endor, you must acquit".

13
Actually it is the author of this article, and not the study itself, that is guilty of exaggerating what science says about gender differences. The reason why articles suggesting that science says gender differences are huge get more air time is because articles are often written by irresponsible reporters, like the author of the present article, who use those studies as a basis to engage in sensationalism. The general consensus amongst scientists is that there is an interaction of nature and nurture. The fault lies with people like this very article’s author projecting false statements into the mouths of scientists. And if papers instead chose to emphasise only those studies pointing towards the role of nurture, that would create an equally false impression of the curent state of scientific knowledge and opinion.

14
Be very careful with genetics, biochemistry, neurology and molecular biology. It takes 10 years of grass roots scientific training in this field to understand the very basic principles of these things.
I hold a PhD in medicinal chemistry, worked in science for a few years and I don’t feel competent to judge the findings discussed in this article.

I've tried to find a CV of the author of that article, in vein. He covers a lot of themes from space travel to environment. This biased article is just entertainment.

15
If I remember reading Ian Sample's original Guardian post which sparked Robin McKie's reply, Verma's study sampled about 1,000 individuals aged between 8 and 22 years of age. The study found that up until the onset of puberty, there were no significant neurological differences between the brains of males and females, and differences became apparent only after puberty.
There is literature that states that late-maturing teenagers are superior to early-maturing teenagers in spatial reasoning. Late-maturing boys are superior to early-maturing boys and late-maturing girls are superior to early-maturing girls in spatial reasoning - and on average, girls reach puberty earlier than boys do. So apparent neurological differences between men and women may have more to do with the ages at which individuals start to undergo puberty. Puberty can be seen as a process which stops or delays certain forms of mental development in certain directions. In many Western countries, the age of menarche (when a girl begins menstruation) has been decreasing since the late 1800s; in some countries, menarche may occur as early as 8 years of age. There have been some news stories about girls experiencing menarche as early as the age of 5 years. Some suppose this is due to children's exposure to chemicals in modern environments.

16
Uh oh, scientific research in political incorrectness alert! Man the keyboards!
There are clearly differences between male and female, and it is quite reasonable for scientists to study these differences. To be fair, 99% of the sensationalist headlines are written by journalists, "opinion" writes and their editors, so you can look to your colleagues for that spin, not scientists.
If understanding of the brain leads to better treatment for depression, autism etc, that is a good thing. Even if research shows differences between men and women, that has no bearing on the moral dimension of their rights in society. If people think it does, they are daft.

17
Intelligent, well informed, skeptical and committed - Guardian writing at its best. Thank you Robin for dispelling the hype.

18
For a science writer to write some nonsense like this is really, really depressing (so depressing in fact that I think I am going to have to down half a bottle of Jack Daniels.)
People have known for donkey's years about the effects of sex hormones on neural development e.g in the amygdala. This is just a *fact of life* (not necessarily taking about this study as there are a couple of problems with it).
The whole nature vs nurture thing (as a few people have referenced here) needs to be thrown of the window, it's old and a *gross* over-implication.
You have genetic, through to epigenetic effects on the foetal brain (which also probably is the mechanism in homosexuality), epigenetic effects in childhood culture etc all playing a role in the neural growth and function. Nobody is denying that there is neural/synaptic plasticity that occurs throughout life, from the moment we're born to the day we die (pretty much), however subtle.
But it is undeniable that women's brains will be different from men's in many respects....and it is down to evolution...evolution doesn't give a shit about gender studies.
The application of values to this is not partially relevant...these are findings (whether you accept these findings is another matter) and observations...and people should just deal with it.)
Finally, people who still have a problem with this should do a little basic reading on the subject and them move onto a book like Molecular Mechanisms of Hormone Actions on Behavior.
Now...where's my goddam drink.
NB: The climate change denier analogy made by a previous poster is entirely appropriate.

19
I have always been of the opinion that we are equal but different. The notion that our brains have differences is no more controversial to me than the notion that our bodies have differences. A large part of the function of the brain is to manage the operation of our bodies, it's not just for thinking with, so it would be rather surprising if we were actually wired the same.
The behavioural consequence of that difference are far more difficult to specify though.
As for the article, this bit made me laugh:

Males develop improved spatial skills not because of an innate superiority but because they are expected and encouraged to be strong at sport, which requires expertise at catching and throwing.

I never was any good at sport at school, and could not catch to save my life. Sometime in my mid 20’s, I discovered by accident that my technique was wrong. Instead of trying to track an object as it moves, I found that if I just keep still, my brain knows automatically what the trajectory is, and where it is going. Suddenly I could catch very well after all, so it seems that the requisite wiring and the ability were there all along, I just had to let it work. However I aquired my spatial skills it was certainly not anythng to do with sport at school, perhaps I learned them some other way, perhaps they are just innate.

20
I still take issue with the rubbishing the ’valuable data’ of this study received. I am just curious and not setting an agenda. However the simplification and perhaps downright erroneous claims by the scientists have achieved what they wanted, I guess, which is publicity for their research. The Press’ willingness to blindly and automatically reach for the lowest common denominator of understanding when it comes to scientific research is pitiful. While funding for science becomes ever more tenuous. Why now the "putting it in context" article after the glib and dismissive columnist piece, other than the click bait cycle of hysteria this paper and the rest of the media seem to operate as their lifeblood. Maybe plasticity is the likely cause? No one quite knows yet and techniques like this potentially opens more doors than just gender research. Just stop columnists writing about throwing the baby out with the bath water and fund science properly.

21
I don’t disagree that men and women are different, but I see little evidence of them complementing each other in general. They complement each other in certain contexts, perhaps - but equally they clash in others. Just like two people of the same sex with different mental qualities both complement each other in some contexts and clash in others.
The fact that we’ve survived as a species with these differences means that we complement each other just enough to get round to mating and ensuring enough offspring survive, nothing more. We could clash in every other aspect of life and from an evolutionary point of view, it wouldn’t matter.
I’m not saying that men an women clash in every aspect of life apart from mating, because that would be too much of a generalisation and provably wrong. The assertion that men and women complement each other perfectly is also too much of a generalisation and provably wrong.

22
Love women, even those ones are clever. It’s not important!

23
Of one thing I am certain. People’s brains seem to be wired so that they have the least problem believing whatever they want to believe.

24
Wait so women are not more suited to child rearing then men? Breast milk? Whether it is social or biological it does appear this dynamic is the most persistent in most ’cultures’.

25
I will cut to the point.
The truth is, the world is a tough place. If you are not fit, you will die. Mother’s who undergo stress while pregnant, pass on this stress epigenetically (increased risk of heart attack, obesity etc.) It is an extreme evolutionary advantage for a brain to be developed partially in a male vs a female direction. There is a pre-pubertal testosterone jump that allows the testes to drop, this also helps to laterallize and pattern the brain in a male fashion.
Like it or not, there is a difference between male and female brains prior to birth. This is the way mother nature made it.
Natural selection does not give a "hoot" about liberal equality. I’m very sorry to have to break this reality to those who do not accept that.

26
Interesting what you write about stress. Way back when I was a medical student, I was privy to the research of two neuroscientists in that field. The results of their studies were unequivocal: hormones released under the effect of stress on pregnant women inhibit the production of neurones in feotuses. Naturally these findings were kept under secrecy at the time because of their political incorrectness. They put an end to the myth that we are all born equal. Granted, brain plasticity plays an important role in adapting to such differences but our initial potential at birth, in terms of number of neurones, is directly influenced by the effects of stress.

27
Women and men who are ’famous for’ and who are ’good at’ some of the activities you have mentioned are 2 different calculations. I work in the creation process of fashion and have always been surrounded by more successful women than men in the same field. Whether they are famous or not does not detract from their ability. I equally know many philosophical women who will sit and talk philosophy as good as any male. I suggest that in history it was harder for women to be taken seriously in philosophy? To be a great chef is admirable, and many of the top chef’s are indeed men, but to be a great cook is a fairly normal activity carried out very often by the female at home. In my family it is certainly the women who get more creative in the kitchen, but I also know a handful of brilliant chefs/cooks who are male. When I was at art college the ceramics course and sculpting classes had more female attendees than male. As for architecture and software design, I agree that more men are involved in and famous for these activities.
So some areas are stronger led by males and others by females, but relying on who has become famous in *some of* those fields may be more to do with ego and drive, and public acceptance than ability.

28
One would expect more from a "science writer of the year" than a polemic like this one. It is possible to write a criticism of a study or a position without being snarky and shrill. This reminds me of Frank Turek’s writing in "I Don’t Have Enough Faith to Be An Atheist . . . minus the snark.
"As I have said, I have read this sort of thing before. I didn’t believe it then and I don’t believe it now. It is biological determinism at its silly, trivial worst. Yes, men and women probably do have differently wired brains, but there is little convincing evidence to suggest these variations are caused by anything other than cultural factors." In other words, don’t confuse me with facts, my mind’s made up.
Scientists are in the business of challenging assumptions with the express intent of identifying the limits of of our knowledge . . . or how badly wrong we are. Pseudoscientists do research to find data that confirm their beliefs. If Mr. McKie is truly interested in taking a scientific approach and look for data that challenge his beliefs, I recommend the evolutionary psychological, neuroscience, behavioral psychological, social psychological and sociological literatures on the phylogenetic and ontogenetic bases for the differences between male and female neurophysiology and behavior.

29
The evidence of previous research show we use some parts of our brain and not others and this depends on need. So a doctor will use different parts of the brain to a F1 racing driver and in each case these areas become prominent. Later in life some parts of the brain are then switched of permanently depending upon need.
This research seems to back up that theory as as the author says our culture does expect different things from the genders which would create different patterns in boys and girls as the part of the brain being used would respond more strongly to the expected norm.
Example girls given dolls, boys given computer and computer games. Even if girls and boys are given computers girls get given soft games (Sims, Disney etc) and boys hard games (Warcraft, battlefront etc)

30
Oh Dear. Robin loves labels - and heavy ones at that. Biological determinism. Waw. How does she possibly know without researching? Why should she be propping up once again the "Venus and Mars cliché" - even if to deny it - which nobody in this story but herself ressurected is for me a mistery!
The question should be whether the results of the research she is conjuring back from the "Proceedings..." of something are true or false. If they are true we have to work out why and in Science that is made with cleverly thought experiments

31
I just don't understand how the "science writer of the year" is still framing the debate in the obsolete "nature vs culture" paradigm.
Neither "hard wired" nor fully set by cultural factors, the brain is first and foremost characterized by its adaptability to biological factors. See for instance Deacon Terrence W. "The Symbolic Species: The Co-evolution of Language and the Brain".

32
hang on a minute !
one set of us can be in the kitchen making food, whilst on the phone, whilst
nudging the dog's water bowl back to its proper place, whilst listening
to the little one in the bath , whilst wondering about what to write for tomorrow's brief
and the other set of us , when in the kitchen and asked a question
will say
' oh can you just wait - i am trying to fry and egg !!"
I think there's a bit of brain difference there and not all down to
the boys wore blue and girls wore pink theory.
mind you we can not forget our beloved hormoans.
they have certainly turned women into raging lunatics and
raging lunatics into kitchen whizzes.

33
my suspicion with the verma paper is:
900 people were scanned. That's minimally 900 hours of scanner time. It's a vast undertaking.
Presumably the subject pool is being used for other things, but unfortunately a technologically much better, freely available data set comprised of more subjects is presently being gathered and already available (the stuff so far).
She'd have started about 5 years ago or more before that other thing existed.
So, she's under pressure to get something before her expensive data is obsolete. Maybe what she was going for originally didn't pan out. She mined the data, found a pattern and spun it.
It probably does show **something** is different between the two groups, just not what she is claiming.
So, it was the hunger games. She needed something from this grant, or she might not get the next.
Just conjecture. Wholly made up. Never met her or heard of her before this.

34
I dare say that in such complex system as a human being (male and female) there is unlikely to be a simple answer (unlike in the Hitch-hiker's guide to the galaxy).

35
So, you're saying that the fact that male Nobel Prize winners for science and economics outnumber females by something like 100 to 1, is due to sociological factors.

36
There is little evidence to suggest differences between male and female brains are caused by anything other than cultural factors
The triumph of political dogma over scientific reality.

37
If this mother nature person existed as an actual entity, I would spit in her coffee for many, many reasons. Thankfully nature is not a god-like entity and humans have evolved because some mutations were better for reproduction that others, not because it was planned that way.

38
This article and an earlier opinion piece in the Guardian worry me. There seems to be a clash of science and culture rather than good science and bad science or good culture and bad culture.
The 'male/female brain wiring' report lives or dies on whether the published results are accurate and repeatable, not on whether it promotes or frustrates the promulgation of sexual equality.
The report should be published (if only as an example of good graphics) because it is interesting. The arguments for sexual equality should be published because they are equally interesting and ultimately more important. But science is not culture and culture is not science.

39
I'm willing to wager that the author of this claim is not trained in any of the life sciences:
"I didn't believe it then and I don't believe it now. It is biological determinism at its silly, trivial worst. Yes, men and women probably do have differently wired brains, but there is little convincing evidence to suggest these variations are caused by anything other than cultural factors."
Actually, no, there is increasing evidence that the male and female are genetically programmed to be quite different, as we would expect. In terms of sheer robustness in the face of brain trauma, for example, the female brain has long been known to be significantly superior.
In fact, it's hard to imagine that any scientist - let alone the "Science and Technology Journalist of the Year" - could write such, well - let's call it what it is: nonsensical opinion. It reeks of unconscious prejudices and half-thoughts of the following illogical form: "Women and men should be equal (socially), therefore they must be the same in all significant respects" or some even less articulable, non-discursive "gut feeling" to that general effect. Neither science nor the cause of social justice for women are advanced one iota by dogmatism of this sort.
I almost wonder if we'll be reading in a future column from Robin McKie how the apparent differences in male and female genital anatomy are also due mostly to cultural expectations.
I accept that there might be different positions on the science of this issue, but the following of McKie's assertions descends to a completely unacceptable level of specious malice:
"A male brain was depicted with its main connections – coloured blue, ***needless to say*** *..."*
Yes, it was Robin, rather a nice blue, too - not that it matters in any way at all, or would EVEN IF the corresponding main connections in the diagram of the female brain had actually been shown in PINK. Which they were not, were they, Robin?
Pathetic.

40
Why do some people try to deny that men and women are different? It is supported by scientific research, and it is supported by what most people experience in their day to day lives.
There is a massive difference between a "scientific fact", for example, scans prove that men and women's brains are wired differently, and a conclusion or opinion derived from that premise.
Men and women's brains are "wired" differently. That can be proven via specific analysis. That does not however mean that either sex is better than the other one. It does not conclusively prove whether these physical differences are created by nature or nurture. It just states a fact.
Don't deny the "scientific fact" just because it may lead to conclusions that you don't like.

41
…there is little convincing evidence to suggest these variations are caused by anything other than cultural factors. Males develop improved spatial skills not because of an innate superiority but because they are expected and encouraged to be strong at sport…
Why is it so important to explain the genesis of the innate in a naturalistic sense (as a genetic endowment) rather than as an assimilated 'second nature'? Given that human nature is imitative (that it must construct itself due to an instinctual deficit and its intrinsic cognitive complexity), what counts as innate must depends at least as much on one's conceptuality than on the result of playing off nature against nurture, or demoting one and favoring the other.
The point is that predispositions evince a kind of automaticity and necessity that assimilate them to the category of involuntary endowments regardless of their process of origination. And that they then come to exercise a constraint on behavior similar to genetic (or congenital) determinants.
The genesis of a predisposition is of secondary importance as it pertains to function. Once it has been assimilated any skill from the most primitive to the most culturally complex functions to determine behavior as a necessary condition and/or end. Our imitative nature has consequences for the notion of innateness and skews the attempt to reduce predispositions to their genetic or epigenetic precursors. In an important sense we make our nature and once made are predisposed to exercising functions in ways that become automatic, autonomous, even involuntary. There is learning within the innate (predisposition) and innateness within learning (environmental triggers of genetic dispositions). This idea of predisposition straddling the different universes of discourse validates both the notion of the epigenetic qua interaction between endowment and environment and the over-determination of human behavior that proscribes monocausal explanations. The problem is that that proscription precludes the search for quantifiable causes, and therewith the scientific explanation of behavior, which is necessarily reductive.

The obvious explanation of any behavior is cultural-anthropological or psychological. It is only in an age of scientism that explanations in terms of ends and reasons need to be defended against reductive naturalistic explanations. That should not preclude our appreciation of the fact that the latter have a contribution to make to the explanation of human behavior as seen from the outside (geographically). After all, over-determination means that our diverse perspectives (explanatory paradigms) are complementary. Assuming that the theoretical work of critical-evaluative conceptualization integrating perspectives (co-determinations) is in fact being performed. Cashing-out of the blank check of complementarity in a higher synthesis is a challenge that cannot be met in natural-scientific terms alone.

42
It is pretty simple really, they are different. The male and female brains are different. Different body chemistries, and where does the brain reside, yes, in a body. A human body.
It is all in the chemistries. Deal with it somehow!

43
One more note. My daughter is a urologist. Urology is a male-dominated field, and many male patients are nervous with a woman doctor. So she has definitely taken sexism in the medical world. She has been extremely competitive in a very male society. Anyone would think her a feminist. A few months after her first child was born, she stated the obvious. Mother Nature is sexist.

44
Biological determinism is a branch of eugenics and racist Science, the differences between the **fluid** structures in Male and Female brains is also influence by environment change the environment and you change the pattens.
Equality and social justice can not be set aside by the trivialities of selectively used Darwinist molding to underpin socila policy.
Survival of the fittest and Greed is good as an underpinning argument for economic policy is a case in point. It does not work and is subject to a constant string of destructive episodes. Essentially biological determinism is used to create barbaric social and self destructive structures that support abhorrent human behavior, shame really

45
More dumb nonsense about male/female brains. It seems to me these so called scientist of the 21st century know less than Jose Delgado did back in 1965, or so they would have us believe.
Just another heap of BS to have the sexes pitied against each other.

46
Epigentics, simple as dismantles any idea of ’tabula rasa’.
Positive publication bias is a nightmare, granted, especially in medicine, what with that being a serious area of public concern.
It’s good they don’t require blank slates, as they don’t exist in the real world of actual biology. This just shows the circular and pointless nature of most of those whole groups of ideas.
It would seem that 100% are a mixture of both.

47
You’re correct: The prevailing consensus amongst scientists is that there is an interaction between nature and nurture when it comes to brain development and personality. No self-respecting scientist would actually engage in the sort of extremist thinking this author attempts to project into their mouths. By attempting to address a serious issue in such an irresponsible and false manner, this article unfortunately adds to the stereotype about the extremes of feminist thought. Some feminists are quite capable of noting the harmful influence negative stereotypes and socialisation can play without engaging in such nonsense as completely dismissing the roles of biology, or the existence of differences between males and females.

48
Do you not think that pregnancy, birth and essential female nurture (the feeding of the offspring) demonstrate the compelling need for nature to ’design’ (maybe by the hit-and-miss approach of blind suck-it-an-see evolution) the male as defender / hunter / gatherer whilst the female could concentrate on her allotted role for as long as it takes?

That’s not ’social delegation’. Yes, in the modern world there is great opportunity for overlapping roles and whereas in a few thousand years that may change the natural abilities designated us, we’ve hardly taken the first step down that road. Evolution strolls and never runs, not matter how much of a hurry we may be in.

49
I sometimes think that science wastes so much time in trying to prove or disprove the blatantly obvious. Take 10 average female drivers and 10 average male - is there any serious doubt as to which of the two groups would excel in reverse parking into a limited space?
We humans have one particular aspect common to all life and that is that mother nature gave us a sole purpose: reproduction. As far as she was concerned anything else living matter did was irrelevant, but she did reward satisfactory and frequent reproduction with an occasional tweak of DNA, genes or whatever to ensure that survival of the fittest kept the strong moving forward and the weak sliding into oblivion.
She determined that we would reach the golden goal of quality reproduction by splitting the function down the middle - males and females. Of course she knew that for her reproduction formula to really work she would need to tweak a bit more by adding ’a sexual imperative’ because surely, who would put themselves up to that degree of commitment and suffering without there being a powerful and almost uncontrollable force to make you blind to it? And no less, having embarked on that route, she knew she would need to focus some distinct and special abilities on both male and females to get them through to the final stage of nurture. She therefore made them a bit different, each with their own but equal qualities.
Now tell me, isn’t that blatantly obvious?

50
I have been reading this same sort of nonsense since my teens (I’m 70). It was used as a reason for choosing boys first for ‘hard’ science and math classes in my high school (imagine doing that now - so progress); it was used as a reason for rationing places in university course with a quota being imposed on female applications (medicine, JOURNALISM, and, of course engineering etc); it was used as a reason, in fact, to maintain women’s supplicant position in society.
I might have been interested in a study such as this if it actually followed the individuals for a period of some years and watched the development of the brains correlated to life experiences. Surely these folks know about brain plasticity. Right? Or was the point to publish because funding was at stake?

51
This is an astonishingly ignorant article. There is in fact a wealth of well supported evidence to suggest that male and female brains are wired differently from birth, and this evidence far outweighs that pertaining to cultural explanations. These differences are also consistently observed across cultures with completely different stereotypes of men and women, and are easily identifiable from birth for even the most disinterested observers. Conveniently, these differences are also nicely explained by another well-known scientific discovery: EVOLUTION.

52
The problem with saying that the differences between male and female brains are purely due to cultural conditioning is that it doesn’t explain gender dysphoria. Transgender people have a very strong sense of being a male or female "person" inside a female or male body, and the only rational explanation for that which can justify the hugely invasive surgery needed to "correct" the problem is that there really is an objective difference between the way that male and female brains develop and these people genuinely have grown up with a mismatch between their external physical development and their internal mental development. And that can’t be a matter of cultural conditioning.

53
I think the point that needs expressing in this needlessly emotional debate is that we are shaped by both biology and culture. Culture and language have to some extent freed humans from their biology but on many levels we are still apes. Nobody would get upset if a study showed that male gorillas have different brains to females, so why this reaction to humans? Like our ape cousins, our brains have been shaped by evolution and it would be absurd to suggest that evolution would create the same brains for both sexes given that each sex faced very different selective pressures (e.g. parental investment).

If people like Robin Mckie suffer this involuntary reaction to any suggestion that male and female brains are structured differently then they are in for a frustrating time as future brain research, due to accelerate in coming years, will doubtlessly reveal many more sex differences which cannot be explained by "culture" alone.

54
OK, so now what do we do ? It would seem fairly obvious (to most) how he-males & fe-males are a trifle different. Not too many scientists would argue with that assumtion (especially when walking along St.Tropez beach). Why therefore is it so controversial to assume that mind architectures are also slightly different ? Let’s assume they ARE indeed different, without entering into the mine(d)-field of ’better’, ’superior’ etc. He-males have been dealing with somewhat different issues since they got off the trees, and no doubt even prior to walking across the planet. It would therefore seem reasonable to assume their mental geometry also developed according to specific tasks that needed their attention, and likewise for fe-males. So what’s the big deal ? Where’s the issue ?? Where’s the nearest pub ???

**23**	**07/12/13**	**Scientists are from Mars, journalists are from Venus**	**The Irish Times**

1
Beware of ANYTHING you read that confirms your beliefs. We find it hard to believe that which does not suit us and easy to believe things that suit us. "Tell me your circumstances and I will tell you what you believe". T.N.

**27**	**06/12/13**	**Toy story; THE BIG READ Why should what children play with be segregated along gender lines? CLARE DWYER HOGG speaks to the parents fighting back**	**The Independent (London)**

1
The point is- why do girls want pink lego? because they are told pink is for girls and blue is for boys, not because they actually like pink. If your group is below the age when they know better than to pick something because they are surrounded by it then you would probably get a different response.

2
How many times does it have to be said, The toy industry does not "gender" its products to enforce stereotypes, it conducts market research to determine to whom the toy in question appeals to and then market it as such. Think about it, why would they cut off half of their possible revenue for no reason?

3
I'm sure you were good at maths and all power to you.
Fact is, the really excellent Nobel Prize winning mathematicians were mostly men and not because they were men, but because they were doing better maths. This is something which has a good deal of research backing it up. Doesn't stop you from doing it, because it's one of those subjects where people can be judged on the merits of their work alone.

**35**	**05/12/13**	**Fix the TV and take out the garbage**	**National Post (f/k/a The Financial Post) (Canada)**

1
A lot of money in mindless research into the obvious could be saved by acknowledging it.

**37**	**05/12/13**	**Juliet Dunlop: Men and women driven differently**	**Scotland On Sunday**

1
This is all fine and dandy, and, as the article states, something that has long been suspected. The real problem is, however, that the male traits have always been lauded (by men, of course) as superior and, indeed, in the more extreme cases throughout history, as the norm (Adam's rib and all that guff). In order for humans to achieve the best results in life, a balanced combination of the two sides of the equation appears to be preferable. This has yet to occur in our history. Perhaps some university somewhere in the world could carry out a study of how to achieve that goal? Millions of women will remain second-class citizens until they do. Surely, human rights supersede all other considerations? Again, who has the courage to state this openly as the much-respected Mandela did against apartheit based on the colour of a person's skin? Having the two hemispheres of your brain linked, in tandem with female genitalia and breasts ought not to be the basis for placing women on a lower rung of the species ladder, ought it?

**41**	**05/12/13**	**It's official: Men, women are wired differently**	**The Times of India (TOI)**

1
In universe it is quite natural for female multi tasking skills in Maternal dominant societies who bear children,bring up them,save them and train them skills of living before aligning from mother to live independently?!

**44**	**04/12/13**	**Women wired for success..; ...but men best at throwing**	**Daily Mirror**

1
That’s strange because I just looked up the top 10 companies in the world and every single one of the CEO’s are men. And what a strange coincidence that this article was written by a woman...

46	04/12/13	In brief: It is pop psychology, not science, that di-vides the sexes	The Guardian (London)

1
A lot of connectivity is determined in early childhood, so you can’t necessarily say this is genetic. Personally, I want to concentrate on those sideways connections!

2
This is the headline from the university’s website:
Penn Medicine: Brain Connectivity Study Reveals Striking Differences Between Men and Women
It would be interesting to find out whether authors Ruben and Raquel Gur object to the headline. Or perhaps they will disagree, given their brains may be wired differently?

3
I am a woman. I like to read maps and I do. not. multitask.
Oh, and I despise pink

4
The previous article didn’t mention anything about mental illness, only the last sentence, still not clear about the research itself, the differences justify the different types of mental illness? How is that correlation explained, both articles fail to explain that.

5
Instead of over-reacting about the backlash on females (eg "voting") perhaps it would be good to acknowledge the differences, and that males act like (uh, well) males and it would be a good idea if we didn’t allow them to vote or be a politician until they became (uh, well) less male.
The world would be a safer place, don’t ya think?

6
It seems it’s OK to say that women are different in a better way (they would make boardrooms less macho and all that guff) but not to say they are different in a worse or neutral way.

7
What this article ignores is the fact that the connectivity between the hemispheres occurs during childhood and can be greatly affected by the conditions a child grows up in. This, combined with the general plasticity of the mind, means that our brains are a product of our upbringing. Men and women show differences in their brains because they are raised differently, so to speak of those differences as somehow ’natural’ is misleading to say the least.

8
No I mean that you have demonstrated in your posts that you do not understand the article.
This article is pointing out that many people are criticising the research for things that the research does not even touch on. You have demonstrated this error in every post you have made on the subject.
Your comments show that you have predetermined your response before you digested the information. You object to the possibility that men and women may have a different brain structure because your idiology demands that this is not so. You offer no reason to doubt the research other than it reaches unacceptable conclusions. What is worse the conclusions you are afraid of aren’t covered by the research and are not implied by it.

9
It is no coincidence that this kind of ’research’ is finaced and carried out in the USA where women’s rights are quickly rolled back, faster and faster.
It is a pity that people in the UK, who should know better, are so quick to agree in this determinist madness.

10
You could argue that women are incapable of focusing on the job at hand---multi tasking often being a euphemism for never being able to complete anything.

11
Sure, don’t oversimplify science, but this does suggest strongly that social constructivism in its simplest forms is junk (or, you might say, riotously oversimplified). We knew that anyway, but good to have a bit of evidence.

12
Evidence that differences are physical is evidence that they are not socially constructed. So on the most recent available evidence a view that places everything onto social construction is wrong. It’s still quite possible that social construction plays an important role, although not necessarily an exclusive or dominant one.
To give you your dues, sometimes the absolutely bleeding obvious is, unfortunately, wrong. If that’s the case here, then please explain.

13
Yes, I should concede on the first claim. There shouldn’t be any fear in a subtle argument that acknowledges the role of physical and social factors and that tries to see how they interact. However, remember that social construction arguments have followed and do follow through to political campaigning; campaigning does have real world effects on real people (discrimination by me or against me has legal consequences); and the theory and evidence underlying these real-life consequences is often very thin indeed. If you don’t believe me, go on a run-of-the mill diversity course.
So, yes, I totally support a more informed and refined debate and accept your criticisms. However, first, this may blunt the political force and utility of the arguments in this area. Secondly, it may even suggest that the legal and social pressures that we are subject to in respect of gender discrimination are based on an overly crude representation of human life. Obviously, that can’t be a blanket excuse for sexism, but perhaps on all sides there is a need for humility on a question that seems to be even more complex than we thought it was.

14
"It is difficult for a woman to define her feelings in language which is chiefly made by men to express theirs."
Thomas Hardy

15
"we are talking about averages"
Yeah that’s your first problem, most people don’t really do averages.

16
Multi-tasking? That cross-brain activity?
" ... which Shakespeare possessed so enormously - I mean Negative Capability, that is, when a man is capable of being in uncertainties, mysteries, doubts, without any irritable reaching after fact and reason".
John Keats

17
This is all very depressing - I’ve already been the silent and unfortunate witness to a couple of blokes reading just the headline of an article about this subject (not this article) and chortling over how they never understood women but this gives them the legitimate right not to even try.

18
These "studies" have reared their ugly heads for many years. Small-scale studies like this one pop up and tell us women should be stuck in the kitchen or low-paid jobs while the men go out there and do the REAL work.
The problem is that these types of study have been comprehensively discredited more than once. Indeed when meta-studies are introduced it would appear that there are very few differences between men and women’s brains. The problem is that some academics need to raise research funding, and research funding generally comes from people with money, and money is always held by those who benefit from the status quo. In this case the status quo is the patriarchy which wishes to put women in their place as a source of cheap or free labour.

So it doesn’t take much imagination to understand where the publishers of this "research" are coming from. Doubtless this "research" will be discredited in due course but not before the researchers have got a grant from the Gates, the Carnegie or the Walton Family...
In any case, if women really are better at multitasking than men, then we should immediately replace all males in manegerial positions with women and prohibit men from all management or administrative posts, as an economic imperative. Their lack of multitasking skills in these jobs must be a serious drag on economic competitativeness.

19
Seriously? This article does not denegrate women in any way. It does not say that women should not be in good jobs, nor does it say that men are in any way better than women. In fact this article is fighting against those who have misinterpreted this study to suggest that the research’s findings support such ugly theories. Really, you should read it again.

20
I’m just not sure why your ire is directed solely at Guardian feminists. I’ve read some vitriolic comments on here by both men and women alike. (No differences there then!)
That’s simple, this paper will criticise any research which claims to show sex difference between the sexes, but will accept research that shows the opposite as gospel.
The Guardian feminists are a prime example of a group who abuse stats and scientific research.

21
I was never in doubt as to the difference between men and women’s brains and general outlook on life so would not argue with this survey, but in my experience it has been evident that women are better at multi-tasking, perhaps this is due to role models and requirements in the family.
Is it essential that we are given all of these irrelevant facts, are there not more important subjects that could be investigated ?

22
There is a difference, too, between how our brains are wired and how we feel and behave.
I’m not aware of that work; care to furnish the references and the link to the acceptance speech of the Nobel Laureate?
These things often fall into the territory of psychoanalysis, which is much maligned by those who’d rather lump all of humanity into categories
Well it’s much maligned for a reason; it’s nonsense.The problem with psychoanalysis is that it is difficult to be wrong as anything can be explained, and there is no evidence with which it assesses any idea or claim as Lewis Wolpert correctly observed.

23
Very welcome sanity in your article Oscar. Glad a lot more of this kind of attitude is finally getting some much needed press.
Esp liked, "It’s not about saying that geezers like to read maps and birds like to have touchy-feely chats. It’s not, as Gur was at pains to point out, about dividing men and women."
Thanks so much :-)

24
You don’t have to look at feral children. Normal children’s brains showed no sex differentiation in the study. Depending on your preference, the finding is consistent with either the effect of hormones at puberty or of societal conditioning through childhood and beyond. Or both.
I’ve not seen the detailed data, but I would guess the spreads for both genders are large. Certainly there are plenty of people of both sexes who don’t conform to the stereotype, and presumably their brain structure must reflect this.

25
What many people seem to be forgetting is that this research has nothing to do with male and female behaviour. It’s about how neuron connectivity in our brains gives us a greater tendency towards certain things - a tendency, not an absolute certainty.
It does say that the brain connectivity in the two sexes seems to back up stereotypes about innate skills: coordination and empathy for example. (Other article on Guardian) But that doesn’t mean that

only one sex has a particular skill, just a greater tendency towards it. How much a particular skill is used will either strengthen it or weaken it.
Skills and behaviour aren’t the same thing. Though they can both be taught and reinforced (or the opposite - inhibited and repressed, to a certain extent).
Role playing is an important part of socialisation. If you give a young boy dolls, he will happily play make-believe games with them, just as a little girl will do with a train set. But young children also pick up on how men and women around them behave and behave accordingly. We all want to fit in which is why our behaviour is determined by our environment.
If you teach your daughter to play football and orienteering, she will strengthen her motor and coordination skills. If you encourage your son to talk to people and care for a pet, he will strengthen his social and nurturing skills. We all have these skills in varying degrees, but this research suggests that there is a physiological reason (differing neuron connections in the brain) why men and women find some skills come more naturally than others.
We tend to like and do the things we find easy. Which means we could be constantly reinforcing gender based tendencies towards certain skills, making those skills stronger because, through repetitive use, we’re keeping particular neuron pathways open.
This does NOT mean that only men can perform spatial awareness tasks, react objectively or take on powerful jobs; nor that only women can be nurses, teachers or take care of the house and children. But by understanding how our brains work, we may find ways to enhance those skills we would like to improve. Or more importantly, how to treat those suffering from mental illness.
Why is this such a scary concept?

26
Agree with the article. I don’t know why people get so hysterical and irrational about this kind of thing, it doesn’t prove much. If everything that could be misinterpreted by goons was covered up then we wouldn’t be getting any nearer the truth would we?

27
In reply to Arabrab, one of the scientifiic findings (see for a review for example Su, Rounds, & Armstrong, 2009, in Psychological Bulletin) is that men and women **choose** different careers. That implies that even in a socially just society, you will get fewer female engineers and computer programmers, and fewer male nurses and kindergarten teachers. But we often hear that the underrepresentation has nothing to do with biology, but that it is the result of an unfair society alone. If biology is involved, then this has major implications for the societal debate about equal representation in jobs, etc.

28
This research proves what we *really* knew all along - that in the real world it’s got to be "horses for courses", not absolute equality.
You only need to look at the sporting world to see that.

29
Traically the scientists are still desparately trying to attribute mental illness to brain wiring

**47**	**04/12/13**	**Multi-tasking: Women have better memory, attention**	**Hindustan Times**

1
another typical useless biased research in favor of women this time conducted my a indian female professor who somehow landed as an associate professor in an american university. Don’t know how? maybe to conduct such a useless research. No matter how many research you conduct mrs/miss ragini verma, the fact is that men are performing better than women in each and every field in reality especially in India inspite of the fact that women get all sorts of facilities and reservations in India. So men are much superior to women whether it is single- tasking or multi- tasking.These research do not mean anything in reality. they are just for time pass to make women feel good and proud. You should conduct those research in India then you will get different results and will clearly show how men outperform women in every fields including multi-tasking.

49	04/12/13	**With motor skills legendary around several DVLA test centres, I'm a stereotypical woman. And no worse for it**	**The Independent (London)**

1
I’ve noticed that men don’t have the ability to brag about multitasking, probably because in the male brain it doesn’t come up as being an impressive sounding technical term that will make other people think you are clever. The male brain would identify any man using it as a tosser or a female.

2
"Men are often taller and broader than women, but what does this say about their relative abilities?"

Men are generaly more powerful and stronger than women

The scientists go out of their way to say one sex is not better than the other but the differences compliment each other. They also point out men and women suffer different mental illnesses which they think is due to these differences. They don’t say there is no overlap, they mention the differences at the extreme.

I don’t think I am the only one who thinks men and women generally have different artistic sensibilities, I think you can certainly see it in writing and art. That is not to say one sex is more talented or better than the other, just different and that is what the scientists are saying.

We shouldn’t allow political correctness to be a reason to reject knowledge by claiming the scientists are saying something they clearly aren’t saying.

3
"In fact, perhaps the findings put a whole new slant on gay and lesbian marriage, which, instead of being merely equal to straight marriage, could now be seen as the top tier of harmonious coupling. Men with men, women with women. All your yin none of your yang. Less wiring, fewer differences, a decrease in angry sparks. And that’s just science." - That’s called clutching at straws.

51	04/12/13	SARAH VINE: Our batty brains will help women takeover the world	Daily Mail

1
It’s not about taking over the world is it? Gender equality is not about suppressing men or belittling them. We need both genders. I am a woman with two boys who are entitled to a place in this world. If we were reading something about men taking over the world, then there would be accusations of sexism.

2
vive la difference!

3
C’mon Ladies, much as I love you all lets face facts. Men invented piratically everything you use and enjoy. The Telephone, The Computer, The Jet Engine, The Train, the Motor Car, Etc Etc the list is endless. Without us you would still be scratching around in caves so lets have no more of this nonsense and concentrate on your hand bags

4
Women’s greatest inhibitor to success is women. An office full of women is hell for a woman to work in, so several women inform me. Women generally prefer to work for a man than a woman, so I’m told. However women’s biggest problem is their love of favour, which they like to call chivalry. Sadly you can’t have equality andthe licence of chivalry that gets you preferential treatment. Now you’ve got equal pay, you must pay your way socially. Yes I know a "real" man has to buy you everything and put you on a pedestal or he’s "tight".. But if a man does that, you’re not equal, are you? Equality or chivalry, take your pick ladies.

5
Of course, wasn’t for the male specie ancestors of Homo sapiens we’d still be living in trees

6
Good point Southdownlad, I worked in a school which was riddled with females who could not seem to detach their emotions from their ’professionalism’. When they mixed, the reaction was like adding Potassium to water. Those women are ’running’ these schools and this is a major part of the reason why the schools aren’t doing so well at the moment, it is so sad.

7
Reply to Susie - Not only are there more women but the only men that can tolerate the environment are softies in comparison with what young lads require. Hardly any male teachers from an earlier era, would last two seconds in today’s schools, because they couldn’t tolerate the PC clap-trap and the important bit that’s totally misunderstood is that it was these teachers the the lads respected. NOT the tolerant easy-going ones !

8
Those silly men are so useless they are only fit to be MP’s.

53	04/12/13	**Men's brains go back to front, women's go side to side**	**National Post (f/k/a The Financial Post) (Canada)**

1
There is considerable variability among individuals within a population, i.e. not all men exhibit typically male characteristics.

2
There is a superb book, 21 years old now, on this topic that had the feminazts in a state of uncontrollable apoplexy with its well documented case for what this recent research has "uncovered". It was called Brain Sex by Anne Moir and David Jessel; I have a copy still on my bookshelf and refer to it from time to time. It points out, as does this more recent research, that the two sexes are indeed very much different in how their brains work. The metastasizing forces of hardcore militant feminazm (which I juxtapose justifiably to the legions of legitimate feminism who seek not to enslave men but merely wish to be able to use the photocopier without some creep pinching their posterior ...) will rail against such heresy but ultimately it will be to no avail. The neurosciences are essentially unequivocal in their findings. So perhaps we can avail ourselves of these marvellous differences to advance the state of humankind.

3
Sure thing Cain. That's why everything you lay your eyes upon anywhere in the world was 99.9% of the time created by a man. Neither is superior - just better at different things. This must be hard to swallow for deluded feminists like yourself.

4
The oppression of women was a requirement for the fact that a man (Mr. Gutenberg) invented the printing press and a woman did not have the chance to.

5
"Being great with things, requiring focus rather than multi-tasking, makes them better inventors. If women were in charge for the last 10,000 years we'd still be living in grass huts."
--
As the article says: "females outperforming males on attention."
Attention is a good trait that can lead to focus as well.
The last part of your statement is questionable. You presume that, if women had been in charge, that they would have oppressed men and other demographic groups - the same way men have done to others. Chances are likely that if women had been in charge - men would have still been able to go to school and get an education. Even today, humans are held back from progress - because of the fact that a very large chunk of the world's population does not want women to go to school or work outside the home. You ignore the fact that human advancement has been held back due to large-scale wars: for example, the World Wars and Cold War set Europe back by quite a few generations. Violence has consistently held human society back - we continue to see this even now in many parts of the world. Due to the fact that women are lesser inclined towards violence - there is the possibility that if women had been in charge for the last 10,000 years - that the world may be a better place now than it currently is.
There is also the possibility that if "women had been in charge for the last 10,000 years" - we would see that an equal number of women and men would have been allowed to be in charge today, because women would have allowed both men and women the opportunity to advance society.
The study provides some evidence of this. Quote: women "...are more collaborative in terms of arriving at a solution that works for everybody."

6
Well that's the first time I have ever heard someone believe that women could be oppressed in Scandinavia. If that's the case maybe oppression is just a natural state, kind of like the heart of darkness is ever present.

7
"What we do know is that we can override those sorts of potential evolutionary mechanisms."

So you admit that the modern notion of "civilization" requires over-riding our instincts. Our instincts tell us that it is insane to dig into our pockets to give foreign aid to people who hate us. It is purely because of ignoring our instincts and conforming to some written principle that we do the things we do.
In other words, our instincts are as I said they are - warlike.
"Some men constantly make the claim that men
fight wars for women, etc - and we know that this is not true"
We do NOT "know" that this is not true. You must be very naive if you do not know of cases where a woman has dumped her bird-chested boyfriend when asked out by the captain of the football team, or that you don’t know of marriages where the wife nags the husband because he doesn’t make enough money, and eventually leaves him for some shady "businessman" in an expensive convertible.
Men, on their own, don’t care about luxuries, fine clothes and furniture, etc. They acquire these things because it makes them more attractive to women.
Sociology and physical anthropology is rife with academics who are cherry-picking data that is politically correct, while studiously avoiding *factually* correct info that doesn’t fit their make-believe world view, and these fields are not considered "hard sciences" by serious thinkers.
see more

8
It’s true that men compete for fun - play poker, tennis, etc., and team sports casually - but I’m talking about serious competition. Men don’t care to hear men going, "Ooo, aaah", they just want to walk off with the trophy and all the perqs of being "top dog" in that field. They don’t think about "why", any more than they think about sex as "making babies".
Fact is, if the choicest women were consistently more attracted to beta males, bird-chested geeks, and low-income peaceniks, there wouldn’t be this competition for wealth, territory, power and higher standard of living, and there’d be no need for wars.
The rest of your theorizing is too bizarre to comment on.

9
Right because saying men create things is a racist statement. What logical gymnastics you are capable of. Look through the history books and I don’t care what colour or race the person is... what sex... what gender has done the bulk of invention? I guess if you’re losing an argument you have to change the argument. Is saying that men are more inventive also an attack on gays and lesbians? Mayans and Egyptians as well as Romans and the Greeks pioneered mathematics - which gender within these groups do you think was responsible?

10
Well we would all like to believe that, most of these studies are as equally questionable as the studies which find women are more often the victims of abuse (without even asking men).
There are so many variables that are difficult to impossible to account for that you can write the science up to tell whichever story you want... and they do unfortunately. Taking scientific data to far through over interpretation is a common problem unfortunately.

11
Yeah, but gender is a societal construct, like herpes...

12
What is next a scientific study that shows there are differences between the body types of men and women?
The scientific evidence has been around for decades.
The anecdotal evidence has been around for thousands of years.

13
As usual, this article fails to note that there is considerable variability within each population and that some individuals do not exhibit the characteristics that are typical of their sex.

14
What is found, and how it is interpreted, AND how it is written to the public IS bias. I could tell this was written by a woman after a few paragraphs.

15
I also don’t believe that women should be combat soldiers, nor do I believe that female prison guards should work in male prisons (and vice versa, male prison guards shouldn’t work in female prisons). Reason why I’m not considered a "feminist" - since I clearly don’t believe in "equal" rights - and I think many feminists might be dismayed if people attribute my beliefs to feminism.

16
True, what? That men should be banned from working with children?
I think men should largely be banned from working with children - in an educator role such as teachers or coaches - but not in scientific research positions (ie, child psychology) and professional positions (pediatricians, child psychiatrists, etc), I think single men and gay men should be banned from adopting children, and I think men should be banned from becoming criminal court judges. However, those are opinions. My opinions on that actually bear no relevance to articles like this.

17
Lol. Good point. If mom’s not happy, nobody’s going to be happy.

18
There are always statistical outliers, that’s a given. No need to state the obvious.

54	04/12/13	**Women crap at parking: Official**	**The Register**

1
A more useful measurement would be to express that as a percentage of the total number of drivers. As it stands, the figure is fairly meaningless, as we have no context.
(Not saying that male drivers are the epitome of safe and careful drivers - but I have also witnessed female drivers doing equally stupid and dangerous things as men do).

2
..."a whopping 80 per cent of crashes ... involved male drivers"
No. Men drive more miles, is all. This is why insurance forms ask for mileage. The average number of crashes *per mile* driven is almost equal between the sexes overall, but actually slightly higher for women.
All arguments about "men are better at X" or "women are better at Y" are rubbish. The sexes' abilities are exactly equal IMO, but their interests differ. Women could do engineering, they just choose not to. Men could be empathetic homemakers, but they have no interest. It makes them a good team.
The "multitasking" comment is particularly daft. It is an typical piece of manufactured opinion designed for consumption by the unintelligent, using an impressive-sounding word borrowed from computer studies to make it sound vaguely scientific.

3
I think what he means is that, from experience and what is said in this article, men are more likely to be doing the 'big drives' so while a women driver in a year could have a bump on the school run, back into a wall while parking or take out a wing mirror in a car park by not paying attention to her surroundings, her husband is more likely to flip the car at 70mph after clipping someone else.
The husband might be a better driver, but as the amount of mile covered goes up, and the average speed increases, the same mistake made by the wife 3 times only needs to be made once by the husband for a major accident.

4
Also, as the article itself says, men generally do the driving and do drive more, so of course the percentage is higher, you have to consider the total numbers of both sexes driving for it to make any sense. Without this info it could well just be stating the obvious.

5
I ask this for no other reason than to be an awkward sod: how does the whole transgender thing fit in this? It's all good and well to confirm a stereotype, but a theory is tested by its edge cases.
And there's nothing more fun than poking at stereotypes (evil grin).

6
Interesting question.
To which I'd add the observations that there are plenty of men who are naturally cooperative and social, and plenty of aggressive target-oriented women : what do their brains show?

7
This is not a new conclusion, less tech was used in the past but came up with the same conclusions. One interesting point made previously was that male nurses and female engineers displayed opposite results to those of their gender. It would appear that the gender of the body is less relevant than the 'gender' of the brain.

8
Ah, yes, multitasking -- or, as I like to call it, "not paying attention".

9
"greater significant differences within each sex than between them"
I've seen this point raised in many places following this kind of study, as if it somehow discredits or invalidates the findings. This seems bogus to me. So what if the within-group variation in a metric is larger than the between-group variation? The between-group variation is still a real, measured fact of

the world. For example, it is perfectly true, and in some contexts useful, to say that men are taller than women. The fact that some women are taller than some men, or that the range of heights in either group is larger than the difference in the means, is irrelevant.
People seem to hate generalizations, especially about gender. Don't panic! Generalizations are fine, and essential in many areas of life. What's NOT ok is assuming that a particular generalization will be true for all members of the population. While this should be obvious, many people don't seem to realise it. So, maybe women are worse drivers on average, but one really can't assume anything about any individual woman based on that. Still, at the population level, it's worth knowing, and is essential for calculating insurance premiums fairly, etc.

10
Not in my case. My wife is a better driver than me - probably because she's done a number of driving jobs in the past (including a London bus for 3 years). I navigate though.

Also gives me the opportunity to drink....

11
>What should have been said is that a woman's brain is subject to a different environmental pressure system than a man, and thus creates synaptic connections in a different way.
Possibly. The experiments to settle the nature vs nurture debate re gender characteristics in children up to the age of five are easy to design.... but they would be unethical in the extreme and very expensive to boot.
In the meantime, there was a radio programme about transgender people, and their experiences in early school life which would suggest there is more going on than a gender-neutral baby brain being shaped by the societal norms around it. Also, there are observable structural changes in a mother-to-be's brain during pregnancy, to help her better deal with the new life tasks ahead of her. These changes are biological, not societal in cause.
Is is possible that your view is informed by a very laudable idealism?

12
The researchers, as they are normally do, made at least one unjustified claim. Namely, the reasoning behind why men drive and women are passengers.
It has nothing at all to do with the stated reasons. Rather, women ride because they are far more manipulative then men are and are much better at getting others to do things for them. A man prefers to take "control". A women prefers to retain control while letting the man believe he has any at all. Notice how women have historically been in control of family finances while staying at home letting the man do the work.

58	03/12/13	**The Difference Between Men and Women's Brains Revealed?**	**The Blaze**

1
It said men are better at parking, not driving. That said, I'm much better at map reading than my husband though I know the old adage, that men are, because they comprehend an inch equaling a mile lol.

2
@thekuligs: On the contrary, most women I know are busy multitasking while driving, but instead of focusing on all of the different tasks on the road, they are on the phone, doing makeup, chatting away, thinking about work, etc, etc. I am afraid to drive with my wife in the driver seat because her mind is always on other things. Then she get's annoyed I'm not listening to her ramble on while I'm focused on the road.

3
funny my husband couldn't parallel park anything to save his life EVER. Meanwhile I could parallel park a big grand caravan with 4 kids in the back without thinking about it. And I can carry on a phone conversation, load a dishwasher and have 2 or 3 IM chats going on the lap top. Fold laundry and change a diaper…pretty much all at the same time.

4
OH and when it came to putting toys together at Christmas .. I win hands down. We bought 3 of each so they didn't fight. I would be starting on the 2nd toy and he would still be reading Put part A into Slot B.. Then he would get pissed because I was better at it than him and all I had to do was look at the picture. The difference is Right Brain vs Left Bran learners.

5
No, liberals don't have a brain, so they couldn't use those for the survey. Sorry guys.

6
Hunters and Gatherers, it's in the gene at the cortex. How much research money do I get now?

7
So, my wife should be able to cook AND clean at the same time.
I told her, so.

8
This explains why my husband can't breathe and wash dishes at the same time.

9
In all seriousness. Is anyone surprised at at the results of this survey. For any right thinking human being that has faith in God, did we honestly doubt the differences between men and women. I guess knowledge isn't common. The women in my family will tell you there are some things their husbands are better at than they are, and vice versa.

10
Men are the fighter pilots. Women are the AWACs. We got your six, now go and conquer.

11
Way to go with this "announcement"…. I'm sure it makes some people happy to know but I wish they had kept it to themselves.. Now I have to try to deflate my teen daughter's continued feeling, especially with this new evidence, that just because she is better at doing multipile tasks at once it does not mean she will not be distracted by her phone in the car and cause an accident. thanks a lot.

12
Finally! – an answer to the ago-old question: If a man were marooned on a deserted island somewhere in the middle of the Pacific ocean and there wasn't a woman within a thousand miles of him… Would he still be wrong?

13
The study used ages 8 to 22. Why? That makes little sense for a brain study. The brain is obviously still developing in 8 year old children. As for 22 year old young adults, the front lobe has just reached development for reasoning and problem solving — and that age is still being challenged.
I can’t imagine why parents would give their consent to a brain scan for their children.

14
This data has been around for 30 years. However, it’s been suppressed by the Leftist-Bolshevik Media because it does not fit their social-re-engineering agenda that men and women are “identical” and that all “perceived differences between the sexes” are “learned”.
Dr. Shad Helmstetter is but one honest brain scientist who has been publishing these data since the 1980s. Men and women are equal, but not identical. Equality is God-given and can not be granted by the State–only God–and therefore the atheistic leftists can not deal with true equality.
Like most Marxist theory, all outcomes can only be measured in terms that the State can control and measure, such as economic, or physical, etc … True equality is not “achieved”, but rather is God-given, genetically-determined, hardwired and created. Therefore, the atheistic Leftists can not control it any more than a bank can participate in zero percent financing from a car manufacturer.
Basically, women are equal to men because God created them equal–not because the State says so. Which explains a lot about why the atheistic State fawns all over issues like “gay-marriage” and abortion (which places the State in the position of determining a false “equality”, on one hand, and a false “unworthy to live”, on the other). It also explains a lot about why the Leftist State hates Christians and Jews.

**59**	**03/12/13**	**Turns out men and women really do think differently, study finds; Findings suggest there are major differences in the wiring of male and female brains; researchers surprised how much the findings con-firmed stereotypes**	**The Globe and Mail**

1
While it is logical to conclude men and women think differently, from the article, it looks like the focus of the study was on the wiring in the brain. That is, comparing the neural connections between the left half of the brain and right half of the brain and between the front of the brain and the back of the brain.

Based on the results of the study as stated in the article, all you can scientifically conclude based on the research is that men's brains and women's brains are wired differently.

As to men's brains and women's brains processing verbal/social and spatial information differently, that's not exactly new. There has been research supporting that kicking around for some time now.

2
This seems to support the observation that has been around for a long time that men seem to be generally better at focused, sustained attention to tasks and thinking - or specialization. Women are generally better at managing disparate, unconnected tasks and "dot-connecting" in their thinking.

As always, there are exceptions to these rules. However, having both styles of thinking around enhances the whole.

3
"it must be assumed then that the men and women in this study were socialized, by and large, to be like their stereotype (girl or boy)"

Men were men and women were women long before the influence of popular culture, media or advertising. They were this way because they needed to be in order to survive as a species - and these traits evolved over millions of years. They are still with us today.

Of course, social influence can accentuate certain characteristics (or attempt to suppress them) but gender differences are not the product of socialization.

4
It is now well established that the brain can re-wire itself - research on stroke victims has furthered this investigation to a large degree. Look up neuroplasticity on Google and tons of articles will spring up.

As one example, it used to be that children who showed an inclination to write with their left hand would be made to learn to write with the right. They were able to learn to use their right hand to write in most cases, however, it doesn't get around the fact that the default wiring was to write with the left hand and it probably would have been easier for them to do so.

5
So learned are thee in the ways of "gender", for it is a mere social construction and there is no such thing as "sex" except for on a spectrum that only you may define arbitrarily and with constantly moving goalposts to suit your own needs. Having studied the canon of the greatest and brightest minds in the hallowed halls of Gender Studies and yay, Women's Studies, we, the Globe and Mail commenters, shall defer all questions of gender and sex to thee.

Now, come unwashed commenters!

Stand behind Life1973 and disregard all common sense. Your eyes have betrayed you. Your life

experiences have been an illusion.

The TRUTH will be brought to us by Life9173, for thou hast thought about this for more than ten minutes in thine lifetime. Unlike you peasant commenters, who have not had this knowledge bestowed upon them by intellectual greats such as... Such as. You know, the famous .. gender ... studies ......

Aw, damn it. Painted into a corner now.

6
Which is why most women will never be good electrical or computer engineers.

7
Yet another false narrative that’s been shoved down our throats by the Left bites the dust. Let’s call this study ’progress.’

**60**	**03/12/13**	**Proof men and women are poles apart**	**The Daily Telegraph (London)**

1
Yes!!! Someone who understands averages and population distribution! Thanks, Forest; most commenters read it as each gender being exactly so and start whining that somebody like you means they don't know what they're doing.

61	03/12/13	SORRY CHAPS, YOUR BRAINS AREN'T MADE FOR MULTI-TASKING	DAILY MAIL (London)

1
On average there are very few differences between male and female brains among young people. The differences emerge as the brain forms neuronal connections over time. Since we seem to live in a savagely gender stereotyped world (evinced by the majority of comments in this discussion) the differences measured are unsurprising. Most of us cannot be objective about gender because we have been indoctrinated on the matter since first we wore blue or pink booties. We come from a time and place in which the maleness of the male, and the femaleness of the female, and the importance of their differences, are matters of almost total preoccupation. It hasn’t always been this way and it will not always be this way.

2
This is why we used to have women on fiddly production lines, because they could assemble components to a high standard and talk away to their hearts content!

3
Generally speaking, men act a certain way and women another. There are sometimes exceptions/abnormalities.

4
Please stop using the word ’designed’! We evolved!

5
Of course, duh! They need to be able to clean and cooke while they look after the children, otherwise they’re no good!

6
The DM ran a science article a while ago saying that there was no such thing as multi-tasking. Make your mind up!

7
It’s been proven that humans cannot actually multitask.

8
Imagine all the feminists fuming right now at the news that they are fundamentally different than men.

9
They found a woman’s brain?

10
What do you mean men can’t multi task? I’ve been lying on the sofa,smoking,drinking beer,watching footy ,eating sandwiches and shouting at the kids to be quiet for years.It’s a wonder I haven’t overstrained myself.

11
Interestingly this article could have been entitled ’Sorry girls but men are more focused on individual tasks’ Why is it that the DM always puts things in favour of women? Could it be that they are deliberately trying to provoke animosity between the sexes? There is a need for both in society. I can do a lot of things simultaneously but it takes longer because I am doing several things at once. My husband csn do the same things individually but complete each seperate job quicker because he is doing one at a time. Net result is that we do the same things in a similar timeframe but get there in different ways. All roads lead to Rome... What many men DO have a problem with is prioritising things that need to be done. Changing a dirty nappy should ALWAYS come before watching the end of a footie game...

12
well multitasking would not serve well hunting or being a warrior you had to be concentrating on the task at hand the distraction would make the difference between success and death while the female

tended the homestead children and often the parents so keeping track of more things was necessary. this is why marriage between men and women is the natural way the paring complemented each other for the good of the family and tribe and then society as a whole

13
Multi Tasking means never finishing one job properly before attempting to do just as badly at another one, men are more focused and have a longer attention span.

14
If this is true....why are men MUCH better fighter pilots than women? You have to be an expert "multi-tasker" for that job.

15
Multitask and achieve nothing

16
949 is not statistically significant.

17
An example is when women and men boil the kettle for a cuppa. The women will do various things while they’re waiting whereas the men will just stand there staring at it lol. Oooo I know!

18
Instead of one ’Good job, well done!’, we have three ’That ’ll do!’ so that the latter will all need doing again in two days’ time., and probably by a bloke.

19
So what they’re saying is men do one complex thing at a time well whilst, women can perform a number of uncomplex tasks simultaneously... why then when my Mrs is ironing and making a cup of tea for me are they both delivered to a less than acceptable standard? Surely both tasks are simple enough...just sayin!

20
Women are the center of attention in many aspects of life like attracting, pregnancy, nursing, listening/caring for people feelings, teaching, home care, working, and so forth. They absorb life. Men just conquer, giving out life and placing their banner everywhere they go.

21
And the fact is men’s brains are larger.. End of story.

22
Women move between topics quickly, men focus on one single thing - common knowledge. Neither is greater than or less than. It’s just how we work. Doing many things poorly is no better than focusing on one thing and missing the bigger picture.

23
The female brain is designed to notice stuff, analyse it and depending on situation act or do nothing. Mum hears and sees everything, (like baby waddling off towards side of the river, or saber tooth tiger approaching) while to an outsider it looks like she is forraging for wild berries. Blokes brains are optimized to be in motion and act. Limbs litarally co-ordinate themselves without you needing to analyse stuff in detail. You can spend hours chasing stuff and trying to kill it (or parking cars lol) without being distracted by the fact that the neighbohr is wearing a fuscia jumper that clashes with her pea green skirt.

24
No s@@t Sherlock!. There’s something that the human population didn’t already know or need proof of!.

25
dont forget we also hold the prize for killing people and the planet ;)

26
Oh Bulltwinkies! So why do men win the vast vast vast majority of Nobel Prizes for discovering everything! Guess all that doesn’t involve "Multi Tasking!" Bunk!

27
Multi task. HAH. My missus cannot drink beer AND watch football; and our dogs best friend us me. So much for research OOL

28
Women multitask whilst men just do one thing at a time. Properly.

29
Hyper - are you serious? All this requires is for the people to be in the same place - obviously the kitchen - at the same time, which is entirely possible. The woman is cooking at the same time as doing the washing up, child at the kitchen table doing homework, baby in a high chair watching proceedings and man telling her what went on at work that day. This is, in my experience, just pretty basic and normal for most women!

30
Multi tasking ? which dress, shoes ,handbag , lippy & mascara to wear there the only 5 thing’s most woman think about ,any thing else would be dangerous ..lol

31
I wouldn’t employ, or even consider employing someone who multi tasks. It is wasteful, disorganised and nothing ever gets satisfactorily completed.

32
Do ten jobs at once, or do one job properly, its your choice.

62	03/12/13	**Is Equal Opportunity Threatened By New Findings That Female And Male Brains Are Different?**	**Forbes Magazine**

1
Women definitely have been given free rein over the past 20 years or so. The Government now promotes women far more then males in obtaining college education and also promotes. Quotas on corporate boards or in Government positions. Affirmative action has been in play for many years but it seems that reverse discrimination is now in full play. Women also receive financial assistance from the Government for being single mothers while males are not given the same benefits even at reduced rates basically receiving nothing. Look at our homeless population it's mostly males although some women are also homeless while the prison population here in the USA is still mostly male but that does make since if the society give women a foundation financially and educationally basically catering to them while the males are shut out of the equation then of course more will break the laws and end up in prison. Watch commercials here in the states and see the reverse discrimination such as "How will you pay for your daughter's education" while one never sees commercials promoting the son to attend college. Why? Or commercials geared towards making the males usually "idiots". Why? You are correct to say men and women both have strengths and weakness but I'm guessing women on average are much more agreeable to power because they don't cause as much trouble to the power establishment as males have in the past such as Union members demanding higher wages or benefits or working conditions for example. Women may voice their concerns and protest to a degree but usually will settle for what the power structure gives them.

64	03/12/13	**Women really are better at multitasking, study of brain wiring confirms**	**The Guardian (London)**

1
From everything I’ve read its almost certainly a mixture of hormonal (and other internal) factors along with external influences.
Some key changes in the brain during adolescence are:
1. A spurt of synapse growth just before puberty
2. Pruning away of many existing synapses
3. Growth of white matter, which increases the speed and efficiency of remaining synapses (by insulating them with myelin)
Though it might seem strange, the "pruning" of synapses is really critical. There is definitely truth to the adage "use it or lose it". Connections that are often used get reinforced, while connections that aren’t used are likely to be pruned away. This leaves enormous room for external influences, even though hormones drive these changes.
To put another way, the hormones are pushing the brain to re-organize into a more adult form - becoming less flexible and more specialized - but a person’s experiences and use of their brain shapes what their adult brain is specialized for.

2
Well may I direct you towards neuroscientist Lise Eliot’s book, ’pink brain blue brain’ (how small differences grow into troublesome gaps and what we can do about it).

3
I don’t think you can say that it is more nature or more nurture. It is both, and in each individual it may be more nature or more nurture.
For individuals born with the same degree of ambiguous genitalia due to partial androgen insensitivity syndrome there is a 25% gender dysphoria rate regardless of sex of rearing - that is, you could raise the same child male or female and they would identify with that gender (and gender role) 75% of the time. This suggests that it is mostly nurture for these children.
For individuals with childhood onset gender identity disorder/gender dysphoria/transsexualism there is a fixed identification with the opposite sex from a very early age, and twin studies show a genetic association of up to 40-60%. This suggests that it is likely a combination of nature and nurture for these children.

4
Not necessarily - if you look at Nakor’s post the adolescent changes are very much a case of pruning away unused connections. Why are these unused? It is certainly possible, even likely, that the answers lie in early childhood.
As I understand it the study looks at connections, not the extent to which those connections are used.

5
Brain adapts pretty well after strokes. Takes a lot to fuck it over.
Adult brains do remain resilient and adaptable, but children’s brains are*more* plastic. Recovery after strokes is actually an excellent demonstration of the difference - if you look at outcomes from pediatric strokes, babies make total recovery far more often than adults.
Fun story: one of my old professors did a lot of fMRI research, and in the course of scanning an undergrad, she discovered that a perfectly normal, healthy, intelligent young undergrad was missing almost half of her brain. Turns out the woman had a stroke as a newborn, but developed normally, so everyone had just assumed there was minimal damage (this was back in the days before imaging was common). In fact, she had severe damage of almost an entire hemisphere, yet the plasticity of the infant brain meant she was able to grow up with zero noticeable impairments.
I’ve never heard of that level of recovery in an adult.

6
I’d be interested in seeing what it is that leads to this change between 14 to 17 yr olds.
I have an answer that should please everybody: it’s s-e-x.
The study needs to be done across cultures to isolate the differences - eg. macho locker room vs arty intellectual. If they’re still there, it might be hormones (which is still s-e-x).

7
I don’t think you can say that it is more nature or more nurture. It is both, and in each individual it may be more nature or more nurture.
For individuals born with the same degree of ambiguous genitalia due to partial androgen insensitivity syndrome there is a 25% gender dysphoria rate regardless of sex of rearing - that is, you could raise the same child male or female and they would identify with that gender (and gender role) 75% of the time. This suggests that it is mostly nurture for these children.
For individuals with childhood onset gender identity disorder/gender dysphoria/transsexualism there is a fixed identification with the opposite sex from a very early age, and twin studies show a genetic association of up to 40-60%. This suggests that it is likely a combination of nature and nurture for these children.

8
Hormones are the driving force behind changes in the body, which can be produced by external or genetic factors. With this in mind i’m guessing your actual question would be: are the changes brought about by predetermined factors such as genetics or do they have an external (i.e. environmental) influence.
The answer would be both, take the onset of puberty for example. Puberty is driven by the amount of energy reserve a person has available. The body’s way of feeding this information back is by a hormone called ’Leptin’, this hormone is released by adipocytes (fat cells). Therefore the amount of leptin fed back to the brain increases as the amount of adipocytes an individual has increases. Once a threshold of leptin is met, the brain begins to pulse a hormone called gonadotrophin, through a complicated subsystem of hormones. Gonadotrophins circulate the body to drive the bodily changes associated with puberty e.g. steroid release, bone growth, hair growth, maturity etc.
Obviously theres many different factors that can lead to changes in the amount of fat cells i.e. malnourishment (environmental) and the ability of our body to store fat (genetic). So unfortunately there is no black an white answer to your question.
To the people discussing the ability of a baby to recover from brain damage, this is also a complicated question, yes the babies brain probably has higher plasticity, however its weaker immune system also has an effect. When the body is injured you can imagine a hypothetical fight between regenerative growth and the immune system. The immune system wants to stave of any threat of invasion so quickly repairs the damaged site, however, this has a consequence as true regeneration takes time due to its genetic influence (genes act slowly). The quick repair leaves damaged ’useless’ tissue behind. An adult has a stronger immune response than a baby, which therefore increases the chance of damage leading to irrepairable tissue.
Anyway, i’d take this article with a pinch of sal unfortunatley a lot of neuroscientific articles refer to brain areas having set functions. Some do, but its impossible to map a road and know the drivers intent. For example its like looking at the east lancs and assuming everybody is heading to Anfield or Old Trafford. Some will be but there’s millions of destinations for the cars to go and billions of destinations for the people in those cars to go. The road is the neural circuitry, the cars are neurons and the people synapses.

9
Yes.
So, these ’brain differences’, what caused them? Are they the product of nurture or were they there all along?
Nurture influences start in utero (see Piontelli), so it would be very tricky to analyse how much pure nature is involved. It starts with the first question: boy or girl, then expands, to the extent that behaviours that don’t fit with parental/societal expectations are frowned upon. Why wouldn’t psychological pressure have epigenetic influences, like any other environmental factor?
Having known about even numbers of men and women with great spatial awareness (and about even numbers without - which-way-is-up blankness affects boys as well as girls) I’m still to be convinced about its innateness according to sex.

10
if that is true, "the differences between men and women" would be mostly constructed and the article above would be only part of some fucked up self-fulfilling prophecy.
No way.

That’s not what I’m saying at all. Brain development is a mindbogglingly complex process of mutually-interacting influences. Genes are powerful, but so are sensory inputs, social constructions, and habits.
Asking whether brain differences are down to nature or nurture (or are constructed vs innate) is a bit like asking whether a song is made up of the notes or the silences between them. You simply cannot have one without the other.

11
The patterns for men lack frontal lobe activity - where thinking and planning occur, but women seem to be extraordinarily rich in such activity. Men’s brain patterns look rather sparse and "reactive" rather than planned. If this is a true representation, then I’d say men’s brains show much more primitive functional patterns than do womens’. I’d also suggest the authors turn their judgment’s around and stop coming at the issue from a male perspective. The man’s glass appears half empty, while that of the woman is apparently more than half full!

12
Most pruning occurs before age 12-13, much at 4-5, and again at 6-7.

13
To believe in equality and equal opportunities, you don’t have to insist that there are no differences between one another. This study simply is showing what a typical scan of a male brain vs a female brain looks like when you compare it’s connectivity, based on a sample group of about 1000 people. There’s a lot more research to do in terms of what causes those differences which could be extremely illuminating in a direction we may not expect.
Even if when we have learned a great deal more about our brains, we find there are simply biological differences between ’typical’ males and females, and that we are fundamentally different, that wouldn’t have to mean anything in terms of the way we act towards one another. It wouldn’t mean that women like Marie Curie weren’t as brilliant as they were. It wouldn’t mean we have to suddenly start judging each other and what we can do on the basis of sex rather than on the basis of our merits.
As for how some people seem to be worrying here about how it may change attitudes; even when racists, homophobes, and misogynists have all the evidence in the world against their position, do you think that will really stop every one of them from thinking the way they do? No of course not. Genetics has demonstrated that there is actually no such thing as a race. We still have white supremacists.
As far as I can tell, there never seemed to be any way in which homosexuals were ever able to threaten heterosexual (or even the closet homosexual) homophobes that still exist all around the world today, in any meaningful way whatsoever, yet homophobia is still one of the biggest social issues in the world, hence the homophobic insistence that there must be some kind of ’Gay Agenda’, or that gay people are attempting to recruit people. There’s plenty of evidence in other species that engage in homosexual behavior yet many wish still to insist that it is ’unnatural’. As well as being flat out wrong, even if it being unnatural were possible, should that justify hatred, abuse, or even violence towards homosexuals?
The worry about what the science may show or imply is not the issue. The issue is and has always been, how small minded people want to treat people who are different from them. The origins of bigotry seem to me to be a more complex issue, more related to their own psychology than one fact showing that two different people may be different from each other. Ask yourself the hypothetical question, even if men and women, or black and white people were found with conclusive scientific evidence to be different in some terrible way, even if the evidence showed something abhorrent to your belief in equality, do you think that we should treat those that were different as though they were worth less? Or that we should arrange our society so that we offer less opportunities to those people? Would that be a society you would want to live in? Would you also engage in that prejudice against those who’d been marginalized? See how far you could actually push the hypothetical difference to the extremes and reevaluate and ask yourself what would it take on a fundamental level, what inherent quality in one or another group would it take to inspire hatred in you for that group. I think this partly illuminates why the bigoted mindset is so despicable. It’s not the science. What matters is how you think.

14
That bogeyword, sexism.
Stands back and waits for explosion

15
As much as that's not what the article says, you can't completely eliminate the possibility from the analysis - cast your mind back to your own teenage years, were you ever so painfully aware of society and what was apparently expected of you as then?
If I've learnt anything through studying psychology, it's that the interpretation of results by researchers is often biased by preconceptions and entirely up to individual opinion. Whilst they have suggested these differences are hardwired, there is absolutely no evidence to suggest so. We need cross-cultural comparisons before drawing that conclusion.

16
That's as may be, but have they got any evidence to back that up? If not, it's very poor form to claim that your results prove something that they simply don't. (Albeit they wouldn't be the first to do so and they wouldn't be the last.)

17
I am happy to have brightened your day. The concept of *male* and *female*brains breaks down when you factor in those born intersex. The dichotomous description of the human condition, and commentator's determinist interpretation of this poorly written article, only serve to diminish us all.
Cordelia Fine's book title may be emotive to you, but there it is. Read it if you dare. If you want something *really* analytical, written by a medical scientist who then try Rebecca M Jordan-Young's book

18
No, because if societal expectation were having an effect you would expect to see a divergence between male and female brains before the onset of puberty.

19
So
I know someone who certainly does have such a thing, and has told me, and certainly doesn't just think they have a slightly enlarged clitoris.
Good luck with that one. They certainly saw you coming.
I think what you might mean is that you, personally, have both ovaries and testes. Or some other definition of intersex.
Keep guessing, it's all you seem to have at your disposal.
It would help your case if you read up about intersex issues before displaying your misunderstandings for all to see. It cheered me up no end tho'. Ta very much.

20
men's brains apparently wired more for perception and co-ordinated actions, and women's for social skills and memory
So it's true. Men are better drivers and women gossip a lot.

21
What does it mean for the men who just can't shut up?

22
And men have more trouble remembering the driving route.

23
Still on the subject of spatial ability and anticipation, I would like to provide another (too typical) crash example. Naturally an example is just that and shouldn't be construed as a generality. My intention is merely to highlight the fact that many accidents are caused by lack of abstraction rather than poor driving skills.
Back in the 70's, my bike instructor was riding along a highspeed section in Paris, similar to that tunnel where Lady Diana met her end, except on the opposite side of the river. He was riding uphill and could only see the green light, not the intersection he was about to cross.
Unbeknownst to him, a woman driving a car had just come from a perpendicular street through a red light. She had slammed on her brakes belatedly, instead of continuing through the intersection, and stalled her engine, leaving her in the middle of said intersection.

Naturally when the biker came in sight of her vehicle, it was much too late to stop and he swerved to avoid her, crashed in a lamppost and ended in hospital for months.
Road stats would read that a man had crashed on his own while riding a bike, as that woman’s car wasn’t hit, but in essence it was her inability to foresee the danger of her actions that caused the crash. She had only reacted to a red light without realising that stopping in the middle of a poorly visible intersection could represent a danger to others and perhaps even to herself.

24
Okay, I have. I’ve observed an induction from a general trend that occurs in male and female sexed brains. So, where do we go from here? We don’t treat these mental illnesses any differently between gender, and we already know that there are differences in the numbers of men and women that suffer from them. So what is the point of this study?

25
"I mean, apparently if we treat two internally pluralistic groups of people separately and homogenously from even before they’re born, that means that their brains often end up different? Who knew?"
Where to start. Firstly, how would you explain the fact that the brains are apparently the same up until the height of puberty? Your argument would make sense if children are treated the same until they are 14, and then suddenly bombarded with gender propaganda for a few months, at which point, no further change takes place. But you have said yourself that it starts before they are born.
Then, you have fallen for the *post hoc ergo propter hoc* fallacy. The fact that we treat boys and girls differently, and they end up different, is not proof of causality. You might as well argue that giving boys toy guns causes their voices to break.
Human society didn’t wake up one day and decide to treat men and women as different. Men and women were different to start with.
Frankly, I think it’s time we were able to accept that their are averagely valid, biologically determined differences between men and women, without having to undo the advances in equality we have seen in the last 50 years.
On the other hand, it seems like this study might help us to understand teenage girls and boys better, and be better prepared to help them all reach their maximum potential. Why on earth would you be so desperate to kill something like that so quickly, unless you care more about your own agenda?

26
"Because it is just looking at a lot of brain scans and making subjective statements."
’Subjective would mean looking at one’s own brain and drawing conclusions.
Looking at **a lot** of brain scans and drawing conclusions is the definition of ’objective.’

27
It prevents me from swearing and getting angry about issues I feel strongly about, which would just be rude (and would also get my comment censored), so I think sarcasm is preferable.
No, not quite what I’m suggesting (although I think we should be more accepting of "male motherhood"). I’m suggesting that many women have many skills that aren’t connected simply to motherhood, so I’m very unsure as to the purpose of this study. It’s not like there’s minor exceptions. There are billions of them.

28
Radical feminists
Is there any other sort?

29
Thirty years ago in a physiological psychology unit at university we learned that the corpus callosum has richer connections between brain hemispheres in women.
And now as a relative late-comer to jazz guitar I understand why it has been so bluddy difficult:
"Musical training has shown to increase plasticity of the corpus callosum during a sensitive period of time in development. The implications are an increased coordination of hands, differences in white matter structure, and amplification of plasticity in motor and auditory scaffolding which would serve to aid in future musical training. The study found that children who had begun musical training before the age of six (minimum 15 months of training) had an increased volume of their corpus callosum."

30
I too was a bit surprised by her very unscientific spin of the results in the last paragraph.
It strikes me that if you were a feminist separatist you might summarise the results as being proof that men and women were far too different to get along.
If you were a heterosexual woman wishing to validate your own need for a man, you might argue that these differences were "complemetary" (as she does).
A scientist should do neither. A scientist has a duty not to spin results in any direction, but to be very aware that others may attempt to do so.
A scientist should state something along the lines of:
In general men have a tendency to be better at x; in general women have a tendency to be better at y; though the distributions overlap considerably and these general results cannot be used to determine how good any one individual might be at x or y, nor should they be used to dictate "appropriate behaviour" of either gender.

31
I'm not playing devil's advocate, but I thought neural connections were 'furrowed out', if you like, through repetition - as in, that's how we learn.
So is the change in male/female brains around puberty likely to be a result of biological determination (in which hormonal changes transform the brain, perhaps), or is it because of the way we're living? If young adults are conforming to these stereotypes will they then develop more neural connections in the parts of the brain that are used the most due to the behaviour they have already been engaging in?
Also, is this a spectral difference or a definite one? Are there grey areas? Can you, for instance, get a woman who has a male brain? Most people's experience in the world would suggest that you can, as some women excel at those 'stereotypically male' activities and are weak at traditionally female ones, and vice versa.
Hoping a scientist will pop along and explain!

32
Biology can be destiny cogenital heart defect for example.
A cross cultural study would be good. Find a teibe where woman do the hunting etc and scan their brains. If the females show the same neuromap as a western male it shows brain develops through society.
It could be that our biology tries to develop brain in certain way but our upbringing can alter it. Makes sense our neuropathways are created everytime we learn to do something the can also atrophy if not used I believe.

33
Its efficacy in explaining human behaviour, however, is by no means guaranteed. Quite the contrary, in fact - despite the chest-beating protestations of macho science fans, there are times when science does not have all the answers
Well, that depends on the questions, and whether or not they are amenable to data-based enquiry, i.e. whether or not evidence can be collected. If it can, then "macho" science is the only thing that is going to likely to give you meaningful answers. Of course, some systems are so complex that the best tools we have available right now can give us only statistical answers and suppositions. Indeed, in general science will never give you guaranteed facts, only best-tested theories. But if you're looking to literature or psychoanalysis to give you guaranteed facts, best of luck with that.

34
Very good.
But science is woeful at explaining human behaviours. Neuroscience is the latest iteration of a tradition of failed scientific explanations of human behaviour that started with the four humors and has subsequently come to include phrenology, eugenics, and evolutionary psychology. (And all of these had men arguing strongly for them in their time, remember.)
You don't have to like Freud, but to claim that wiring is the only way to explain the differences between the sexes and all other cultural, social, developmental and psychological influences are irrelevant - as you are doing here - is just plain wrong. Not least because it presupposes a biological determination that women have been proving wrong for years - if its all about wiring, then, for example, the number of women choosing not to use their wombs in the 50 years since contraception became widely available would not be increasing year on year. But it is - as soon as women had a

choice, they took it, and are doing so in ever-increasing numbers. But by your logic that wouldn't happen because women are hard-wired to want children.
As I said, science has a role to play in the world, and an important one at that. But when it comes to explaining human behaviour, the idea of wiring falls very short indeed.
And more importantly, the misguided belief that behaviours have biological causes leads to all sorts of blind prejudice, as I said earlier. In this regard, you might find this article, on the Bad Science website, of interest: http://www.badscience.net/2010/10/pride-and-prejudice/#more-1801

35
If you put 10 girls and 10 boys in the garden and have treated them identically since birth and given them both dungarees to wear, the same hairstyle etc the boys and girls would not behave the same. Most parents that have both boys and girls know that boys play more aggressively etc. Social changes certainly influence the genders but to think that young boys and girls think the same way is nonsense. They have different hormones in their bodies so why would they behave the same way?

36
What we need is for science to tell us why this changes after 14.
There's something that goes on in the human body at around the age of 13 or 14, but I can't quite remember what it is...

37
"Men and women are different". Homogenously, apparently. Science like this *does* determine life chances.
The science is poor because it provides no explanation for why; it observes a trend and makes an induction. "Because it's biology!" is not an explanation.

38
This is something that will come as no surprise whatsoever to 99% of the population.

39
I think people are reading to much into what the article is trying to say. The study is just a study. If you want to know what conclusions they found read it for yourself.
I agree there is variation but so what...I have done studies where I have to cut off sexes in to groups. I found that certain physical differences correlate to certain behavior between sexes. I didn't try and argue causality just that there was a very strong correlation between a physical attribute and a behavior. As far as I am aware there is no suggestion of any society/genetic origins for the differences. Just that there is evidence that male and females brains work differently after 14.

Of course there is variation within the group. Averages are useful; for example on average men are taller than women. But guess what there are some tall men and some small men. How can we determine anything if certain attributes are not separated? It just gets too complicated.
I really don't see how this is controversial to some people. The evidence does not have to relate to equal rights or go against any agenda (feminist or otherwise). I always say skepticism or advocation in science is acceptable but not when it is born from political ideology.

40
Who's been saying we're the same? Equal doesn't mean same.

41
holy shit, you;'re telling us something that we've known for millions of yrs. that men and women are different. suck eggs fool

42
This does not mean I will listen respectfully to any man (under 60 - polite deference to age) explain to me how to put up an Ikea bookshelf.

43
This can't possibly be correct.Whilst commenting on this article.I am warming up a currry,smoking,drinking white wine,wondering whether to drop Sigurdsson from my fantasy football team,scratching my back and listening to the Smiths.That's multitasking right there .Beat that ladies!

44
I can roll a joint, make crucially important political posts on facebook, read the guardian and drink tea while listening to free jazz music all at the same time.

45
I read the publication and whilst the statistical significance is high (something like p < 0.00001 for many of the results), I am not too sure how great the differences really are between the brains.
For example, there is the t-statistic, a measure of the transitivity in a network, I don't know if when the t-statistic is higher for some networks in male brains compared to females and vice versa for other networks, whether it is so high we would expect to see cognitive differences. The paper does not have a conclusion on that. Is a t-statistic of say, 5, when also statistically significant, a strong figure? Maybe it is not known, I don't know anything about neuroscience, so it would be nice if the Guardian article maybe illuminated points such as these, i.e. what does it all mean, can we quantify in real terms what the differences in brains are?

46
women's brains are designed for social skills and memory, men's for perception and co-ordination
No they weren't. Designed I mean. They evolved. Basic science fail, I'm afraid.

47
For a second I thought this might be true. Then I realised how unpalatable this study must be to my fellow readers so I'm going to pretend they made it all up.

48
it is illegal to state that there is any difference between people,
it is unbelievable that the police have not arrested the researchers

49
On that test, I scored closer to that of a man (50). People are probably picturing me as a butch-looking female, but I'm actually petite and very feminine-looking.

50
It seems almost certain that this is a chemical altering of the brain at puberty, given that the wiring is the same during childhood.
That said, lots of gays seem to know they're gay (and have feminine characteristics) at a very early age. So it's all rather confusing still...

51
Lise Eliot wrote on this. If it's a study done with adult men and women, then due to social and nurturing elements, their brains will become wired in different ways. It isn't like that naturally.
Ah.. perpetuating gender binaries.

52
Reading books is not the only way to acquire knowledge, you discount experiential learning. It is one of the weaknesses of science that this often happens, in branches of science where linear observation in the longer term is seen as either impractical or is discounted altogether. By this I do not mean mere observation of data, but observation of behaviour/activity, whether human or otherwise. It is equally wrong to expect that one source of information will be the only authority, in neuroscience this is now becoming recognised, after a period in which science/medicine/research was all about competitiveness and superiority , there is a general acknowledgement that the brain is immensely more complex than was thought, and we are only on the outer edges of understanding. To journey further will require the interaction of observer and observed in a way that science is presently not fully comfortable with, it will take time for this to happen. When it does then there will be real breakthrough, until then we just have to remember all such studies are a start point, not an end point in understanding.

53
I don't think anyone denies that individual variation exists. However, there are widely observed group differences in the behaviour of men and women that need to be explained. Even social constructivists

accept that these group differences exist: they simply contend that it’s caused by environmental rather than innate factors.

54
Alas, poor social constructivism! I knew it, Horatio: a theory of infinite jest, of most right-on fancy. It hath ruined my social science reading lists a thousand times, and now, how abhorred in my imagination it is! My jimmies rustle at it. Here hung those post hoc fallacies I have questioned I know not how oft. Where be your trendiness now? Your catchy slogans? Your empirical assumptions, which were wont to set the whole STEM faculty on a roar?

55
I was just wondering whether the researcher tried to determine beforehand whether the men and women taking part in the experiment were heterosexual and whether fiuture experiments might be carried out on homosexuals to see if they differ.from the rest.I hope this doesn’t stir up a hornets’ nest, it’s not meant to

56
I think its disgusting that you seek to medicalize normal behaviors as ’ridiculous autistic spectrum behavior’.
What’s disgusting about equating autism with normal behaviour? I think we should be recognising that people with autism and aspergers can achieve great things, and that they are "normal" people too. Many great scientists and inventors (but by no means all) have exhibited autism spectrum behaviours, and I’m certain that some great scientists from the past, if alive today would be diagnosed as such. Look at the likes the Nobel laureate Vernon Smith, who has asperger’s. He’s achieved great things and happens to have aperger’s. It’s been suggested that many historical figures have had conditions of this kind- from Newton and Einstein, to Lincoln, Russell, Jane Austen and Tesla...these people have driven the human race on. They are the most exception among us, and yet some may have had these conditions. If anything acknowledging this can destigmatise labels such as "autism" or "asperger’s".
I’ve spent many years working with people with special needs. There is nothing disgusting or abnormal about them.

57
Love the way the article writes it up - women better at this, women better at that.

58
Here we go again, the Guardians obsession with all things gender

59
Sure it is my conclusion. I’m not talking about the research per se, but the interpretation.
My point (probably not clear enough!) is IF you want to treat this research as demonstrating conclusively that the differences between men and women are neuronal, THEN you have to accept that there is no evidence that the differences before the age of 14 are neuronal (unless you bring some other evidence to the party).
To the degree that you say "well its kind of interesting but not conclusive", then you can say the same about social influences before 14.
What I’m unhappy with is a cherry picking shift in criteria, with a starting point that assumes a neuronal cause for gender differences. So any piece of neuronal difference is given the kind of fanfare we have in this piece. And the fact that the same research could find no evidence of gender differences is unexplored and ignored.
So, nn8275, I’m not saying that it is all social factors, I’m saying the interpretation of the research in this article is simplistic, misleading. If you can prove genetic determinism for behavioural gender differences, good luck to you. This article doesn’t do it.

60
Shame on you Guardian for such poor poor science. No reporting of the actual differences.
Specifically, is the within-sex variance bigger or smaller as the between-sex variance, and if so, by how much ? Or the same ? Range ?
And what of the pitfalls of unwarranted extrapolation from structure to function ?

"On average, men are about 10% larger than women. So their brains are about 10% larger. So they are 10% smarter, right ?"
Remember Guardian, correlations are not automatically causes....

61
My wife can complain about eight different things in one breath.

62
"I was surprised that it matched a lot of the stereotypes that we think we have in our heads. If I wanted to go to a chef or a hairstylist, they are mainly men."
No but seriously an academic said this? An academic? It makes no sense. Did you quote her correctly?

63
Maps of neural circuitry showed that on average women’s brains were highly connected across the left and right hemispheres, in contrast to men’s brains, where the connections were typically stronger between the front and back regions.
Granted, society will be richer if women and men are encouraged to thrive in areas in which they have comparative advantages over each other. But No! -- Says the ideology-driven "gender equality" brigade.

64
On the whole nature vs. nurture debate:
We have plenty of evidence from different cultures and different time periods about gender roles. If it is nurture as opposed to nature, wouldn’t the worldwide and time spanning consistency of gender roles seem to indicate that the nurture is so pervasive, it might as well be nature anyway? How do you possibly explain the world, for all history, consistently deciding what gender roles are supposed to be?
The idea that people are "restricted" or "oppressed" by this is utter nonsense -- show me the guarantee you got at birth that you’d live an adverse free life...
I get that you don’t like gender roles, but I also get that reality doesn’t rearrange itself to suit anyone’s sensibilities.

65
Precisely why kitchens are better off with women rather than men

66
This is hogwash, this sort of thing has been discredited multiple times by Rebecca Jordan-Young and Cordelia Fine.

67
Show gender differences in newborn brains. Then I will take them seriously.

68
so much time wasted fighting for equality when you could have been, instead, defending women superiority.

69
No. This is bad science because of the ridiculous conclusions.

70
Although I’m not in the camp that wants to immediately reject the findings, I couldn’t help but wonder if the researchers took note of personality differences during the study or made sure to have a large variance in that area. Especially when they uses the term "healthy" to describe the people in the study. How do they determine a healthy male/female brain? It can either be very scientific, or very subjective.
Are outgoing, social, or non-athletic males used as often as males they would deem "typical"? What about athletic, socially awkward, or science/math oriented females? (These are only examples... I know it doesn’t cover all stereotypes) or would some of these groups qualify as unhealthy to the researcher?

The stereotypes will always hold if you don't make sure to include people who don't fit those stereotypes. If the results show the same thing with the variances accounted for, THEN I'll believe it.

71
Does to be wired differently make it likely that men and women be inclined to excel in different skills and express themselves in different areas of interest?
Lawrence Summers was once crucified and had to resign for saying something that to me seems tantamount to what this research has found.

72
Some of the posters are right when they say that the average doesn't provide much information but variance only improves the information if the samples are drawn from a Gaussian (normal) distribution (since the mean and variance are sufficient statistics for this distribution). In many of these data sets the distributions are usually heavy tailed ones which of course have infinite variance.
Let's say someone wants to prove women are smarter than men or the other way around. Some researchers compute averages of male and female iq's from a large sample and then compare averages. This is laughably incorrect. One way to proving the hypothesis could be to statistically infer the distribution of the iq's of the two groups and test conditions of stochastic dominance. I have not any rigorous analysis of statistics in this field but I can think of a simple counterexample. A female theoretical physicist (ex: Lisa Randall) would probably be smarter than more than 99% of males so I doubt whether men are smarter than women or the other way around. The answer is somewhere in the middle.
I am a mathematician and as you all know the field attracts people with higher iq's as it is the most rigorous academic discipline. There are disproportionately more men than women in the field although the male to female ratio is much lower than those in fields like theoretical physics, computer science and engineering. More than 25% of Fields medal winners (highest prize in mathematics) are Ashkenzai Jews. But that doesn't make me whine about supposed bias and invent conspiracy theories. They win those awards because they are really good at what they do. Maybe that's what some women should do as well. Instead of complaining about sexual discrimination in male dominated fields, they should work hard and prove stereotypes wrong.

73
GPSs should only be sold to women, and men shouldn't stand for parliament because they're bad communicators.

74
"Women are better at intuitive thinking. Women are better at remembering things. When you talk, women are more emotionally involved – they will listen more."
women are better at remember things?
what are men better at?
could you point out a few of these things please?
sounds like another vauge justification for guardian male bashing.

75
"but it is only when it is rewarded financially and through social recognition that men dominate."
Personally, I think it's more the case that when men dominate it is rewarded financially and through social recognition. Women cooked for thousands of years without inventing or requiring the chef's hat or the Michelin star.

76
Wow ! Men and women have subtle differences in their make-up. Can I have a research grant?

77
Sigh...not this again.
I wouldn't place too much reliance on this, just. This idea flows in and out of fashion with alarming regularity.

78
What about neural plasticity?

"Male and female brains showed few differences in connectivity up to the age of 13, but became more differentiated in 14- to 17-year-olds"
So just how much of this is cultural plasticity?

79
This still doesn’t answer why Mrs Atavism singularly fails to appreciate a good Zombie movie, and why I don’t care why anyone on Downton Abbey is crying.

80
Has there been a similar study to show that people from different ethnicities are or are not fundamentally different?

81
As are people from different ethnicities.
That’s surprising, given that if I looked at your DNA I would be able to identify what sex you were, but I wouldn’t have a clue about your ethnicity.

82
But I suppose the priesthood in various religions sets out to subvert normal male behaviour, it demands celibacy and inner control, silence and mindfulness and pacifism. It is a niche occupation of self-selecting individuals and is usually a lifelong effort for the people who join it.

83
This is the worst piece of pseudo-science journalism I’ve read in a long time. Since socialisation rewires our brains, all this shows us is that the participant’s brains were influenced by social gender roles. The article would have been mure accurately titled "New research shows men and women are born with remarkably similar brains".

84
We’ve know this since the dawn of man. Only in guardian land do you need a scientific study to understand this.

85
I’d be really interested to see the percentage of men who have "female" brains, and vice versa. Could this be a discussion more of "masculine" and "feminine" brains, than male and female...?

86
My neurology only extends to John Ratey’s book (who is an acclaimed neurologist), but I am aware of concepts such as bidirectionality and neuroplasticity that indicate that our brains are very significantly shaped by the environment, and moreover, can be consciously shaped, as is evidence by contemporary treatments for dyspraxia.
So given the above, and the fact that teenagers have powerful environmental pressures to conform to gender stereotype, mixed in with hormonal changes, suggests a far more complex picture that those on the political right like to paint, i.e. that women and men are fundamentally different.

87
I’d like to ask the authors where the hell their supplementary methods text is because as a specialist in diffusion imaging I find you’ve not presented adequate methods text for me to assess the validity of your results.
You used an FSL bayes model. But you mention it nowhere. I infer from the tracking method and default parameters.
Folks it’s a solid paper but diffusion stuff needs a big grain of salt.
They do have a very (very) substantial subject pool and very rigorous analysis. Exemplary really. But it’s still just diffusion imaging. Diffusion is diffusion. not "wires".

88
All that this sort of study shows is how very little we know about the brain and how it functions (or malfunctions).
What is the cause and effect here? What is the significance?
We don’t know.

What is or sure is that small amounts of knowledge can be dangerous, as they are all too easily used for drawing conclusions about general behaviour.
For example, I am NOT good at multi-tasking. Never have been, never will be. I like to do one thing at once, almost to the point of obsession. However, I have a female brain. And deeply resent the fact that a statement could be made that I can cope more easily with work, collecting the kids, feeding the baby (not that I have one any more) doing the shopping, the laundry, and taking the car to the garage. All at once. It’s bollocks. And dangerous bollocks.
Or are we just looking at pretty pictures and not really knowing what we are seeing, beyond our own assumptions. Apart from a few stabs in the dark.

89
“And deeply resent the fact that a statement could be made that I can cope more easily with work, collecting the kids, feeding the baby (not that I have one any more) doing the shopping, the laundry, and taking the car to the garage. All at once. It’s bollocks. And dangerous bollocks.”
Odd, how people can read the same general statement and interpret it differently. You read better at multi-tasking and you think that. I read better at multi-tasking and thought better managers, CEOs, etc

90
Male and female brains showed few differences in connectivity up to the age of 13, but became more differentiated in 14- to 17-year-olds.
So basically male and female brains start out the same, but social conditioning of behaviours leads to differences in the brain - because learning something changes the brain.
So what’s actually new?
It would be interesting to see this study repeated using subjects from cultures with different expectations of gender roles. Would men’s brains from societies where men are expected to be better communicators than women show greater connectivity across the corpus callosum?

91
I’m glad to see that science is catching up with why men and women do have diffferent priorities in the world. it helps me to understand myself better, and why I am not all that great in detailed memory nor at social skilling, whilst on the upside I am better at conceptualising problems and then on dealing with them in a planned way.
What’s so great about multi-tasking? who wants to bake a cake whilst gabbing on the telephone to a friend?

92
I am intrigued. Why is it bad to use science to establish facts? Once we have facts then we can adjust how we do things in order to create the end result we want. So lets say we want to create a society where everybody has an equal chance to achieve in whatever field they like. If this study does indeed identify a fact (I am note assuming it does) then surely this is useful in planning education and developmental activities for BOTH biological sexes?
Why is this possibly bad? It’s an opportunity to move forward our understanding and make changes... You cannot plan how to get somewhere if you do not know your start point...

93
This is an example of a hugely overblown story. There is a great deal of controversy about neuro-imaging and what it really reveals. But even if we assume it is indeed reflecting something real and substantial – that men and women are using different parts of the brain to respond to some stimulus or other – it does not imply a different conceptual or emotional result. Men and women can reach the same results in terms of thought and behaviour using different neural pathways, and this may relate, in part, to the size of our brains. So, the pictures from brain scans showing differences between men and women, which still are so widely used, as “proof” of male focus and female multi-tasking, in fact prove nothing of the sort. After all, left-handed and right-handed people also use different brain circuitry but, as yet, we don’t propose different ways of teaching them, or get them to wear different coloured clothing.

94
"this kind of researches is very dangerous!... Because they don’t reinforce my rigidly defined view of gender".

95
It’s difficult to know what to believe. Wasn’t there an article last week telling us that no one is right or left brained?

96
Those maps explain a lot about women’s concern to save face, where males tend not to be as bothered. Most of the activity is up front - not much going on behind...
On the positive side though, you can see why women are better at multitasking - all those frontal connections keep the balls up in the air when you’re trying to juggle 6 tasks at once.
I have an each-way bet. Men should also learn to multi-task, but I do believe there are clear gender differences that need to be respected - and celebrated.

97
Yeah. Science is such a pain for the orthodox views on gender in academia, epecially in the humanities departments who as far removed from science as they are from hard work.

98
This cognitive issue is reflected in neural circuitry.
You assume that difference will automatically be assumed to connote superiority or inferiority.
Geneticists might conclude that the difference in wiring results from the environment hence the wiring may be more appropriate to that of the individual since it seems to evolve with age.
Male and female brains showed few differences in connectivity up to the age of 13, but became more differentiated in 14- to 17-year-olds.
This differentiation may reflect the gender roles we assign the different sexes. Do some individuals defy the pattern?

99
Not sure whether measuring anatomical connectivity can say much about how ’wiring’ affects cognitive functioning and it certainly does not support the claims made by the journalist - I think this would be a question better posed for functional connectivity analysis whereby you look at the temporal correlation between (’active’) spatially seperated brain regions using functional data (fMRI, MEG or EEG).

100
Compare and contrast... began so many assignments in school. Doing so with gender is rarely useful because the subject is perhaps a little too close to home.
My own bias is that of a man, lucky as a widows son, now old, still curious, a father of daughters in a world, as I see it, increasingly distressed.
Perhaps we would do better to compare and contrast our specie to hyenas or lemmings first, just to get a feel for the exercise.
Humor, famously difficult to chart or do with power point is essential.
ie., If all the debutantes that came out at the Capital City Club were laid end to end, I wouldn’t be surprised. - Dorothy Parker. Or the book by her friend, "Is Sex Necessary?"
Male superiority is chiefly a male delusion. Edwardian’s smugly settled the problem with "Men propose and women dispose" and invented corsets. See how easy patent idiocy is?
Some have noticed, especially in times of social and economic distress, that ’boys will be be boys.. and girls too.’ How much is cranial imaging really going to help sort all this out?
My surmise, to answer Benchly is Yes, sex is necessary, but we, people maybe, not so much.
Unwilling and perhaps unable to face problems that threaten to extinguish our specie, a new calculus is called for, a manner of rationalizing our sexual and other variety. - Newton and Leibnitz formulated calculus to manage relations becoming evident but not explicate by mathematics of their day.
I guess is that what I am hoping for is a like advance in language and ethics to formalize how we think and even feel about ourselves, our relations with each other, our world, which in fact may not be ours at all. Certainly not ours alone.

101
The take away here for me is that the male brain is not "better" over all than the female brain and neither is the reverse true.
Rather they have different approaches to the same problems and would do best working together as a team rather than apart and against each other.

102
I think you’ll find you’ve just contradicted yourself there - just a bit!
All men and women, all cultures and races are exactly the same. - So far, so good. It makes sense, even if you don’t agree with it (which I do).
But then you state that rich, white men hate other people and do everything to bring them down. So are all people the same, except the rich, white men?
Finally, you hit upon the clever idea that anyone who does not agree with you automatically declares themselves to be a fascist and hateful sociopath. Does that include the philanthropic, rich, white men?

103
You may be right, to a certain extent. I certainly believe that culture, environment and socialisation have a major effect on child development. Boys and girls are just as capable of learning new skills and

104
Intriguing that the brain structures start to diverge from similarity from the time in development the most intense gender socialisation kicks in. We know the brain changes itself: I would not read this as the brain gendering behaviour so much as society, it seems, is gendering the brain.

105
In no way have I suggested that intuitive thoughts are not logical. If they admit that intuitive thoughts *are* logical, then the study may start to make more sense. Yet they are left with intuitive thought as a mere subset of thought as such. So they have nothing but *all other types of thinking* to compare it to, since logical thought *is* thought, despite the specifics of**actual content**. If we’re going to deal with thinking and intuition as juxtaposed with logic, then intuition cannot be counted as thought since nothing qualifies as thought unless it is logical. It is no different than saying nothing counting as a square *unless* it has four sides of equal length, four right angles and so on.

106
Why are we do desperate to assume that men and women are born the same, and that physical differences differences are all that separate us? Not everything can be explained by patriarchy and conditioning. It had to be acknowledged that there is an element of nature as well as nurture. Then the question is, how do we get the most out of each other, not how do we transform society into a gender homogenous mulch.

107
Has nobody here actually bothered to read the full scientific report? This article has just taken snippets, which, predictably, focus on the complimentary aspects of male and female brain connectivity (with a slight emphasis on females for fear of appearing sexist).
The study reports that the observed connectivity ’supports the notion’ that females show superior memory for faces and words, along with social cognition, while males show superior spatial and motor skills. However, it also states (which this and every other article does not mention) that males show superior modularity and transitivity, which essentially means that males are superior at specialising at any given task, and that these specialised modules show enhanced communication with one another. This is perhaps the most important and interesting finding of the study in relation to peoples’ everyday lives. But of course, there is no trace of this in any article reporting on this study.. Who would dare say such a thing!?

108
The only way idiots can misconstrue this into "pop-psychology" is by ignoring the fact that
A) This article reported on the "average" population.
B) This article speaks nothing of contributing factors of the results (i.e. genetic influence/sociocultural influence)

109
This is a short article on a complicated research study and its main purpose seems to have got lost in the sound-bites. It is unfortunate that Verma used the term ’hard-wired’ which suggests that the connections within a brain are fixed and new skills cannot be aquired. Perhaps she needs some PR guidance more than anything.

This should have been the main thrust of the article:
"they hope to learn more about whether abnormalities in brain connectivity affect brain disorders such as schizophrenia and depression"
Whether the scientists initially aimed to look at differences in neural connectivity between the sexes or if this aspect only became apparent during analysis is unimportant. Learning more about the brain so that the pain of mental illness can one day be diminished for both sufferers and their families alike, no matter their gender, is important.

110
I'm sorry but your writing seems to highlight a deficiency in logical thought.. And I don't mean that to sound offensive but you're making convoluted arguments based on a straight forward report.
The study lends support to the notion that women, for evolutionary (survival and parenting) reasons, make use of more intuitive thoughts during social interactions - they make inferences based on emotion, body language, tone of voice etc.. There may be an argument to suggest that this intuitive thinking can cloud logical thinking (maybe that's one reason why so many more women believe in the paranormal), but this is only speculative..

111
Who said anything about weaker? Men are more logical thinkers while women are more emotional. On average of course and it doesn't mean that women are incapable of using logic nor does it mean men are incapable of thinking with their emotions. It just means that women tend to sway towards how they "feel" when it comes to decision-making and handling situations while guys tend to take a more fact-based approach. Get it?

112
It doesn't say anywhere in this article that men are more logical thinkers, although it does say that women are more intuitive. I don't think you can draw from that generalisation that men must therefore be more logical. You are attributing an opinion to a scientist that she has not stated.
There is nothing in this article to give the idea that men are superior to women. You are reading into this study what you want to, rather than what is actually there.
Unless, of course, you have read the published scientific paper. In which case, I apologise - one feminist to another.

113
In other words, you dislike these findings and so you choose to reject them. Typical liberal. Accept what feels good and reject what feels bad whether it's true or not.
By the way you don't seem to understand what they mean by logical thought. They are saying that women are more emotional thinkers on average and men tend to be more fact-based thinkers. This is only one of many studies that suggest this is true but liberals like you will never accept it because it doesn't feel good to you.

67	03/12/13	**Turns out men and women are different**	**Human Events Online**

1
Well said. Common sense should always trump the corrosive and toxic twaddle of nihilistic social experimenters and engineers. And chivalry should always reign...should.
Now fetch me my pipe. I'm kidding! Well, not really...it's all the way across the room.

2
Men and woman together produce children. Lefty men with lefty men produce aids. You shouldn't be so mad that you weren't born a man.

3
Testosterone-toxicity the great hallucinogenic.

4
This is racist....homophobic.anti union...anti-obamacar...anti gay...anti-man-made-global warming....or something like that.....Like we need comments on stupid studies that go full circle back to natural law. We knew it then, we know it now. Government must stop funding junk science research. Britain and the USA are filled with this kind of make work for otherwise unemployable Ph.D's....It doesn't lead to a therapy or a drug or increased production. It is make work for the educationally institutionalized.

**74**	**03/12/13**	**Men and women's brains are wired differently, study finds**	**PBS NewsHour**

1
Of course our brains are wired differently, we’re socialized and enculturated differently. I’d be more interested in this if they’d used brain scans from infants instead of adults. As it is, there’s a major confounding factor from the fact that our brains wire and rewire themselves in response to stimuli and they’re trying to make a comparison between two groups of brains that were exposed to different stimuli.

**76**	**03/12/13**	**'Men, women's brains wired differently'**	**Press TV - Iran**

1
There are so many exceptions to this that they become the rule rather than the exception.And I will tell you this for men and women, where there is a will there is a way... whether your nerves connect from left to right or front to back. You are a spirit and you are in charge.

78	03/12/13	**Brains Of Men And Women 'Wired Differently'**	**Sky News**

1
"[Women] are likely to be better "mind readers""

Why does she keep asking me what I am thinking then?

2
"If we are attracted to the "opposite" brain and that is the only thing that shapes our sexuality then straight men would be attracted to gay men. And two gay men wouldn’t be attracted to each other."
--

Good point Abe, but kindly you also highlighted the limitation in your own response...No one but you said it was the only thing which shaped sexuality.

3
These are facts already known. Do some psychological reading. Two good books, ’Men are from Mars. Women are from Venus’ The other one is ’Why men don’t listen and women can’t read maps’ They couldn’t have written them without some facts to go on.

The map of the brain picture is interesting and backs up the reading of Men are from Mars.............’

It is not sexism. It is the differences

**80**	**03/12/13**	**Brains of men and women are poles apart; Differences in the way the brains of men and women are wired helps to explain why men are better at navigating while women can multi-task**	**The Daily Telegraph (London)**

1
I’m waiting for an explanation as to why men won’t ask for directions when they’re lost ;)

**82**	**03/12/13**	**Why Men and Women's Brains Work Differently: It's All About the Wiring**	**Time (USA)**

1
As a male/intuitive-analytical/problem-solver, I was rather puzzled by the comment that socialization might improve intuitive-analytical skills. Indeed, it might be true, but I find that many somewhat socially awkward persons of either gender can also be good intuitive-analytical problem-solvers. Either way, it’s a mystery.

**87**	**02/12/13**	**The hardwired difference between male and female brains could ex-plain why men are 'better at map reading'; And why women are 'better at remembering a conversation'**	**The Independent (London)**

1
@Lee Whensley - I couldn’t find the thread you started that I wanted to post this on.

So here is my theory. Stats are from an estimate of Jungian-based personality types and are listed (Women vs Men estimated % of the population). With the caveat that I already know the souce information is estimated and could be flawed, if you have a better way let me know. http://www.mypersonality.info/personality-types/population-gender/

We need protectors and we need creators. Women and Men are roughly equally suited to be a creator (32 vs 34%). Women are slightly more suited to be protectors (43.5 women vs 37.5% men). These are the bread and butter of society and survival, the overseer, defender, craftsman, entertainer, persuader, artist, examiner etc. and make up the vast majority of the male and female population.

In the category of intellectuals and visionaries, where I would argue higher mathematics, engineers, scientists, and perhaps even doctors, CEO’s, and lawyers fall... only 12.5% of the entire population are best suited to be an intellectual, and only 14% are suited to be a visionary. These are the engineers, strategist, chief, advocate, confidant, mentor, dreamer...

Men are more likely to be intellectuals (7 vs 17%), and women are more likely to be visionaries (17.5% vs 10.5%). But both the male and female numbers together only represent 26.5% of our population.

This idea that the career of engineer or computer scientist is better suited to man because of this "yet another visualization of the differences in neuron mapping" continues to not hold water for me. At most 7% of women and 18% of men are best suited to be intellectuals. The vast majority of men AND women should not be pushed into these careers.

According to: http://www.census.gov/prod/cen2010/briefs/c2010br-03.pdf
Female Population of the US in 2010 is 156,964,212
Male Population of the US in 2010 is 151,781,326

7% of Female = 10,987,494
18% of Males = 27,320,638
Total = 38,308,132

So females should make up 28% of mathematicians, engineers, computer scientists, inventors, CEO’s etc. and men should make up 72%.

I would argue that the difference between the reality and 28% IS DUE TO SOCIALIZATION. And since it is skewed that means that are all sorts of imposter intellectual men who are not really suited to that running around leading companies and engineering things.

2
The way male and female brains are designed and built by the evolutionary process is based on their different execution strategies, as explained here http://www.whatismind.com/MFRIA.aspx

3
This is a quite spectacularly inaccurate representation of a supposedly homogenous feminist narrative of the past 40 years. Large parts of feminist critique in the humanities and sciences has been to draw attention to the remarkable diversity in understandings, representations and experiences of gender across time and culture, to draw attention to the differing values attached to supposedly ’masculine’ and ’feminine’ characteristics. Part of the difficulty with this study is that it starts from an assumed dichotomy of masculine and feminine and operates with minimal attention to cultural influences. It

notes that these differences only show post-adolescence thereby ignoring the impact of 15 or 20 years of extra-biological habitat, coming to the conclusions that, one might suspect, were pre-formed.

4
HUGE ASSUMPTION. This article assumes there are only two kinds of brains.

Everyone is different.

5
It always amazes me how offended some people can become at the idea of generalizations... until they want to generalize themselves.

6
It didn't. Every journalist that has interpreted this research has listed half a dozen functions that point to female superiority but only one masculine function, map-reading.

Multi-tasking, an item on that list, is a myth (just divided attention, a distraction that degrades each of the conflicting tasks) and the claim to superior analytical skills is just unsubstantiated bunk. Where they've grudgingly acknowledged the masculine specialities, it's with the subtext that those are merely the skills a competent grunt need - brawny muscular stuff.

7
Actually; most of us (probably including you) use the conventional order of "men and women" when speaking of both. I doesn't mean we think less of bees than we do birds. It only means we conform to time saving conventions and prefer not having to remember which oder we used last time to preserve fairness.

8
Nope, you're wrong. When kids are little, if they are given the same playthings, the same opportunities, they develop generally at the same rate. And right now, I earn more money than most of my male friends because men are not as adaptable as women. We do what we have to, even if it's traditionally considered a man's role, whereas men do without. The bigger issue is not whether we are ABLE as individuals, it's about the opportunities that are out there and the way people react. Take a look at this
http://www.upworthy.com/some-creepy-dudes-wrote-some-creepy-things-to-this-scientist-so-she-is-calling-them-out-in-public?c=ufb1

9
I find it interesting that most of the people trashing this study, or more precisely trashing the people who tout this study, are in fact men.

It would be interesting to examine the brains of these various suckups and panderers and see how low self esteem causes people to be so pathetic.

10
I thought that the "right brain, left brain" theories had been debunked. No?

11
Sexist pigs! Off with their heads!

12
But, if we put Ms. Noonan up against some brilliant male columnist in a Dishwasher Loading competition, I am sure she would win. The researchers probably didn't think to include tasks such as loading dishwashers or determining if an article of clothing makes one's derriere look big in their tests.

13
You've never met a woman before. They are people, just like men are. Did you go to an all-boy school?

14
An aquaintance of mine recently had a sex change operation (male to female). Said most painful part of the surgery was having half his brain removed.

15
There is no denying the differences between sexes, particularly physically and emotionally, but I don't buy that the sexes are intrinsically predisposed to certain types of intellectual activities. Maybe the physiological brain differences are a result of differing physical activities of the sexes. I spent untold hours as a kid playing with with lincoln logs, tinker toys and legos - was my brothers made cars for a demolition derby and I made houses. I can read a map as well as any man and can figure out how to fix most things just fine. I also am a lawyer who chose to stay home to raise my four kids. I'm not so sure our innate mental abilities are so different as much as our interests are different. My idea of fun is decorating, my husband, not so much. You tend to be good at the things you do often, and you tend to do often those things that interest you.

16
You're confusing cause, effect, & correlation.
Believe what you want, but let the science go forward and see what it says.
It will be interesting to see the same brain mappings in a larger population, cross cultures.

17
Tahni, that can be applied to more than 90% of the population, both women and men. But as far as i can tell, from my experience, women tend to be more emotional. ESPECIALLY when they don't get what they want.

18
There you have it: the liberal war on men.

19
And this is being reported as new evidence why? One quick google and you can find a report from 2008 saying almost exactly the same thing. Why not report it as supporting the previously postulated position!

20
This for the most part is not new information. It has been established for over 20 years that the female corpus callosum, bundle of neural tissue with over 200 million axons which facilitates communication between the two sides of the brain, is thicker than the male's corpus callosum.

21
'Tis interesting the rage coming off some commenters here.
Women and men wanting equal rights is the desire to both be treated as human beings deserving of equal respect, entitlements and treatment.
Obviously we are all different, identical twins are bloomin' different

22
Most of the founders intended for every adult to have the right to vote, but left it at "property owners" lest they lose the support of several of the states.

Not to mention the fact that the majority of families (not every family, though) tend to vote en bloc, so a family with lots of adults could just have their head of household's vote amplified with everyone voting.

23
We are not reading research here, we are reading an journalists opinion based on their reading of a research report. I agree this is just a new "picture" to tell an ages old story, not any groundbreaking research to be sure. By far the most interesting item I see in this article is pointing out that before adolescence these differences were not exhibited, so what gives on the vastly different treatment we give based on gender from 0-13? Why even bother with giving a 3 year old a barbie or a truck, wearing pastels or not, its all going to change at 13 anyway based on this scientific evidence.

24
Personally, I wish every so-called feminist on the planet would go site down somewhere and SHUT UP. Life was a lot saner before they started the war on men.

25
main difference: female brain has been used.

26
Next step is to find out whether this comes naturally or if we force them to grow this way through social conditioning.

27
No no no. It is not social conditioning. You must not have lived very long or you would know this.

28
According to the article, the research showed none of these differences before adolescence (starting to image at age 8), so any differences 8 to age 13 or so must be social conditioning... Right?

29
"The research was carried out on 949 individuals - 521 females and 428 males - aged between 8 and 22."

Ah yes, because 949 individuals are totally indicative of 7.1 billion human beings worldwide.

30
It’s a good sized group for research of this kind.

31
Note that " the way the nerve connections in the brain are “hardwired” occurs during adolescence ". An interesting and probably more useful study would be one comparing women who have different experiences in adolescence. My bet would be that women educated at all-girls schools, with no brothers and a dominant mother* would come out more ’masculine’ on the brain stakes. This of course has nothing to do with sexuality. (* PS I know one such woman - me. And on a pop psychology test some years ago on the telly I came out with a brain 50% male and 50% female. I was delighted.)

32
Where does race come into this?

33
No! No! It is okay to say women are better than men at remembering conversations (or at anything else where they are demonstrably better), but it is very, very sexist to say that men are better at map reading (or anything else).

34
Yes, with the new double standard, only women are allowed to have superior abilities, not men. You see this constantly in films, television, the press, everywhere. It’s really quite old and boring by now.

35
So there is a difference between the male and female brain, perhaps there is also a difference between the brain of a white man and the brain of a black man? Remember that anti-racism ad with the four brains, the smallest brain of the four was that of the racist. I’ve always had a good laugh at that ad. Those who made it are in effect saying that Winston Churchill’s brain, or Dostoevsky’s brain or Robert E Lee’s brain was somehow smaller than the brain of Russell Brand or Mick Jagger, or a slew of celeb whiners and fakes.

No, race and gender, and civilization as a result, do matter. The left are simply too stupid to understand it.

36
Ha ha! I'm male and rubbish at reading maps, but my girlfriend is really good at it. We should start a club ;)

37
The research does not present results that are as absolute as you seem to believe. You appear to have seized on the term 'hard-wired' which is probably only a journalistic introduction in any case. Characteristics and traits are all variable but remain valid in general terms.

38
What a stupid comment, it's an average. Some women will be better at golf, than men. I doubt you could beat the female world champion.

Women may be more inclined to one path, men another, but that doesn't mean there should be 94% women social workers, or 80% men in the board room. To say certain task such as memory are done better is one thing, to attribute career requiring multiple and diverse skills to this is quiet another.

39
Everybody knows we are different as soon as they are born.

Now that it is indisputable, could so-called progressives stop telling us we are all the same with lifestyle choices to make?

40
Hasn't this been known for years?

41
er.....this is The Independent.. a British newspaper. If you are religious it's a private matter in the UK (unless you are Muslim of course). Also "libera' is not synonymous with 'Leftist' as you Americans so strangely think.

42
Well, a few hundred years ago the best minds in science were telling us the earth was flat and the sun revolved around the earth, then the best minds in science were telling us according to their highly scientific calculations done in their highly scientific minds,creation just.......happened,.....you know, Boom! kind of like .....magic, but intelligent design? how unscientific!, and now after only a few short thousands of years of scientific study and observation, These charlatans want us to believe the minds of men and women function differently?Hey C'mon! You've done it now! Hillary and NOW are gonna be so upset they're probably gonna yell, scream, an cry, then scratch your eyes out!

43
But he's right, genius. The Left will be upset about this. Remember when the Harvard Dean had to leave only because of suggesting it could be possible that there were differences between the female and male brain?

44
Are you kidding me? Men and women are different....who knew.

45
well of course women and men are think different.,. now let me know when they do research with participants who have some age on them such as the 30-60 year old ones who have LEARNED to use more than a small portion of their brain for survival,, and are not thinking with their hormones..

46
The world hasn't always been capitalist, and even now there are a few small non-capitalist societies. If people want to talk about "the brain" as if all; human brains must fit that pattern, their research must be genuinely cross-cultural

47
Geniuses! This is how your pinheaded government spends millions of dollars! Congratulations, big spending liberals!

48
So women’s brains are hardwired to make them better mothers. Whooda thunkit? Meanwhile, it is clear that we need to suspend the 19th Amendment and disqualify Hillary Clinton from further political activity--at least until we figure all of this out.

49
One problem with many studies of this kind, and especially with American studies, is that the random sample of people studies is anything but random.
For example I have read data suggesting that generally about 90% of subjects in American psychological studies are white, middle class, American psychology students

50
Women are superior to men in that they are less violent.
Men are superior to women in that they are more analytical.
Overall, women are superior. They can do anything as well as a man ... except VOTE and DRIVE A CAR.

51
Dude, in this world, superior means the one who has more power, wealth, and influence, the rest is academic

52
It is just me, or does anyone else see the "connections" are aligned with a male head that nods Yes, while the female head shakes No?

53
Well Gordon Brown certainly had a mental problem. His brain wasn’t connected to anything.

54
Men and women are *different*? Who knew?

55
"Hell hath no fury like a woman scorned"... Women now-days are very violent. Maybe because socially they have lost their identity? I do not know the reason why, but I have seen senseless female violence jump in leaps and bounds over the last 10 years...

56
What’s new is finding a physical "brain" cause for the "mind" phenomenon already observed.
I believe it was Heisenberg? Dirac? who said "There are 2 kinds of science: Physics & Stamp Collecting".
By that he meant that physics seeks elementary explanations of observed phenomena, whereas most other sciences were still trying to collect, classify & group the phenomena.
This study moves psychology along the path toward explaining phenomena by physical causes.
Genetics is also moving biology in that direction.
This is good. It is also easy to see why Physics is a more advanced science, ala Heisenberg. Atoms are simpler subjects than brains. Newton’s laws & Maxwell’s equations, E=mCC, or even Heisenberg’s Uncertainty Principle are so much simpler than what I anticipate might be equations expressing human behavior may turn out to be.

56
This is actually old news. I read this same finding several years ago. Basically, women are scatter brained, in that they use more of their brain than men do. Men have a much more dominate side. I do appreciate the use of the word "complementarity." It shows once again that the sexes are in complementary relations, which includes sexual complementarity. That is why homosexuality is contrary to nature.

57
Women come in various models! A: Most desirable!

Model A: Can carry case of beer on flat spot on top of head!
Model B: Can carry 12 pack beer on head!
Model C: Can carry 6 pack on head!
Model D: Can carry 1 bottle beer on head! (Most difficult to deal with: No purposeful direction!

58
All that rant, simply because you don’t understand the difference between "identical" and "equal".
As far as I know nobody has ever said men and women are "exactly the same", i.e. identical. If they were, there would be no point in getting more women into boardrooms, for instance, since the results would be identical. What has been the common view is that:
1. Although there are population gender differences, for any given characteristic there is no absolute difference in performance between every man and every woman (e.g. most men could never possibly be combat pilots, but some women can.)
2. Such differences as exist, statistically, do not allow anyone to state that the population of women as a whole (or for that matter black people as a whole, or any other population group) is therefore inferior to any other population group. Value judgements about the "superiority" of certain characteristics carry an intrinsic bias, since they are invariably made by the people who have those characteristics (e.g. I am a rich banker, capitalism is the result of male brain dominance, therefore men are better than women).

59
Actually, most scientist didn’t believe the world was flat. That idea went out long before columbus ever took to the seas. As for the sun revolving around the moon, most scientist had debunked that but because of pressure from the church, it was difficult to write about or support. The big bang theory goes well beyond a magic boom, we’re here, but that would require reading up a bit on physics to understand.

As to this study, it might be reasonable, it might be rubbish, I’m not the one to say. But just making baseless claims then mocking women and NOW is no way to get a point across.

60
I wonder if the faculty of Harvard University will now apologize to Lawrence Summers, their former president, whom they forced out of his office after he made a comment about the different abilities of men and women.
